# Crown Ether Supported Alkali Metal Phosphides: Synthesis, Structures and Bonding

**DOI:** 10.1002/chem.202502127

**Published:** 2025-08-03

**Authors:** Felix Krämer, Michelle H. Crabbe, Alan R. Kennedy, Catherine E. Weetman, Israel Fernández, Robert E. Mulvey

**Affiliations:** ^1^ Department of Pure and Applied Chemistry University of Strathclyde Glasgow G1 1XL UK; ^2^ Departamento de Química Orgánica I Facultad de Ciencias Químicas and Centro de Innovación en Química Avanzada (ORFEO‐CINQA) Universidad Complutense de Madrid Madrid 28040 Spain

**Keywords:** alkali metals, bonding, phosphides, structures, synthesis

## Abstract

Herein we present the synthesis and full characterization of the previously unpresented lithium diphenyl phosphides Li(15‐crown‐5)PPh_2_ (**1^Li^**) and Li(12‐crown‐4)PPh_2_ (**2^Li^**) as well as the heavy homologues Rb(18‐crown‐6)PPh_2_ (**1^Rb^**) and Cs(18‐crown‐6)PPh_2_ (**1^Cs^**). We thus complete the series of crown ether coordinated alkali metal diphenyl phosphides and reveal that the structural diversity increases upon descending the group. Including the previously reported Na and K derivatives, we performed a detailed study of the title compounds in solution that strongly indicated all of them are monomers with an alkali metal phosphide AM–P interaction. We further investigated the influence of the phosphorus‐bound substituents on the bond between the Cs(18‐crown‐6) and the phosphide fragment. For this purpose, we synthesized and characterized two new cesium phosphides, alkyl‐aryl Cs(18‐crown‐6)P*^t^* BuPh (**4^Cs^**) and bis‐alkyl Cs(18‐crown‐6)P*^t^* Bu_2_ (**5^Cs^**). We observed here that the heavy alkali metals Rb and Cs favor coordination via *π‐*arene interactions over the P donor dative interactions. Finally, we used state‐of‐the‐art quantum chemical methods to analyze the bonding situation in the title series of compounds.

## Graphical abstract

**Crowned!** Herein we describe a complete series of crown ether coordinated alkali metal phosphides from lithium to cesium, including solid state structures, detailed investigations in solution and extensive quantum chemical investigation of the bonding situation.

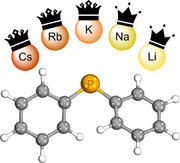

## 1 Introduction

**Scheme 1:**
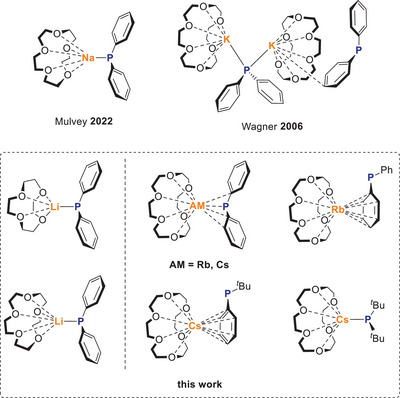
Known crown ether coordinated sodium and potassium phosphides and isolated structures of this work.

Alkali metal organic compounds, especially those of lithium and to a lesser extent of sodium and potassium, have long been among the most widely used classes of substances in the history of synthetic organometallic chemistry. Interestingly, organosodium and organopotassium compounds were discovered first, by Wanklyn^[^ ^1^ ^]^ in 1858, whereas organolithium compounds followed about 20 years later, by Schlenk and Holtz in 1917.^[^ ^2^ ^]^ The phenomenally diverse applications of *s*‐block complexes have been the subject of several review articles emphasizing the importance and indispensability of these utility reagents.^[^ ^3^ ^]^ Until recently, almost all of these applications took place in reactions within the limits of stoichiometric transformations. In contrast, *d‐*block metal complexes, especially of precious, extortionately expensive metal species (e.g., the platinum group metals,^[^ ^4^ ^]^ Ru,^[^ ^5^ ^]^ Rh, and Ir^[^ ^6^ ^]^), have dominated the world of catalysis for many decades. With the advantage of high natural abundance (especially sodium and potassium, the sixth and eighth most abundant elements in the Earth's crust, respectively),^[^ ^7^ ^]^ low cost, environmental innocuousness, and relatively low toxicity compared to some of their *d‐*block counterparts, alkali metal‐based compounds would be a fantastic addition to the toolbox of catalysts for homogeneous reactions. For these reasons, the development of compounds of sodium for various chemical transformations is currently an area of growing interest.^[^ ^3^, ^8^ ^]^ Our group has published over 70 papers in organosodium chemistry, with highlights including the discovery of the NaTMP (with M. F. Lappert)^[^ ^9^ ^]^ and NaCH_2_SiMe_3_ reagents,^[^ ^10^ ^]^ and the pioneering of inverse crown compounds.^[^ ^3b^ ^]^ However, until recently, these works have mainly focused on stoichiometric advances in both homometallic and heterobimetallic compounds. In our latest work, we developed Lewis base donor‐supported sodium phosphides such as Na(15‐crown‐5)Ph_2_P (**1^Na^**) and a mixed TMEDA ligated Lithium‐Potassium phosphide [(TMEDA)Li(μ‐PPh_2_)_2_K(TMEDA)(THF)] as efficient and fast catalysts for the hydrophosphination of alkynes and 1,1‐diphenylethylene respectively (Scheme 1).^[^ ^11^ ^]^ Key advantages of sodium are its much greater abundance in the Earth's crust compared to lithium (24 ppm compared to 16 ppm)^[^ ^7^ ^]^ and the increased reactivity range of organosodium compounds compared to that of their organolithium counterparts. Though there can be exceptions, it is generally accepted that organosodium compounds are more reactive than their lithium counterparts.

The question now arises whether the (nonradioactive) heavier homologues of lithium and sodium (especially in the case of potassium) might also be sustainable alternatives to precious transition metals in important catalytic reactions. Our group has recently provided good evidence for this thesis by showing that the addition of AM(HMDS) (AM = K, Rb, Cs) in the Mg(HMDS)_2_‐catalysed transfer hydrogenation of styrene resulted in orders of magnitude higher activity (HMDS is 1,1,1,3,3,3‐hexamethyldisilazide). The addition of these much less studied heavier alkali metal amides gave yields of 98% or more, whereas lithium and sodium amides gave much lower yields.^[^ ^12^ ^]^ Potassium and especially rubidium and cesium have received little attention in alkali metal‐mediated or alkali metal‐catalyzed reactions. However, their propensity for *π*‐arene interactions strongly suggests that they could have a significant impact on applications such as homogeneous catalysis and the chemistry of low‐valence metal compounds.^[^ ^3a^ ^]^ Potassium is abundant in nature (23 ppm), rubidium, and cesium are rarer (90 and 3 ppm respectively) but equally abundant as lithium, and their rich ores are by‐products of lithium mining.^[^ ^7^, ^13^ ^]^ Recently, Walsh's group has shown that LiHMDS or NaHMDS as additives have a major impact on the selectivity and yield of palladium‐catalyzed cross‐coupling reactions.^[^ ^14^ ^]^ Furthermore, alternative Cs_2_CO_3_ or CsF additives were shown to significantly increase yields in both palladium‐catalyzed cross‐coupling and alkali metal‐mediated reactions where in situ formation of CsHMDS was postulated.^[^ ^15^ ^]^ Subsequently, they found that CsHMDS alone catalyzes the aminobenzylation of aldehydes with toluene derivatives.^[^ ^16^ ^]^

The role of cesium in the above‐mentioned reactions is not described in detail, as it is used here in situ as an additive. Defined, structurally characterized cesium compounds and their application in synthetic chemistry and catalysis have been less studied. We were particularly interested in the heavy alkali metal phosphides, which are clearly underrepresented in the literature compared to the ‐amides. A simple search for corresponding alkali metal nitrogen and phosphorus bonds in the CCDC database makes this clear (Table 1).

**Table 1:** Number of hits in the search for alkali metal nitrogen and phosphorus bonds in the CCDC database.

	N	P
Li	5878	455
Na	2775	115
K	3848	185
Rb	302	21
Cs	277	32

Herein we present the synthesis of crown ether coordinated alkali metal phosphides of the general formula AM(crown)PR_2_ (**1^AM^**) with AM = Li, Rb, Cs; crown = crown ether, and R = aryl or alkyl (Scheme 1). All newly synthesized compounds were structurally characterized using SC‐XRD methods and compared with the known structures for sodium^[^ ^11^ ^]^ and potassium,^[^ ^17^ ^]^ which provides interesting insights into the coordination behavior of the different alkali metals. Furthermore, we investigated the behavior of the whole series of Li‐Cs in solution and analyzed the bonds between the crown ether alkali metal fragments to the respective PR_2_ fragments using state‐of‐the‐art quantum chemical methods.

## 2 Results and Discussion

### 2.1 Synthesis

**Scheme 2:**
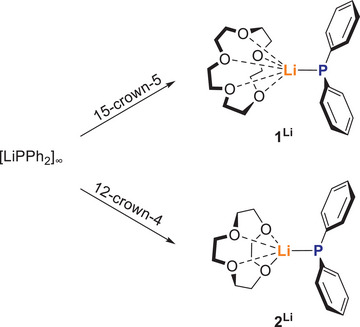
Synthesis of the lithium phosphides **1** ^Li^ and **2** ^Li^.

In order to make a comprehensive comparison of the alkali metal phosphides, we started with the synthesis of the lithium derivative, which is easily obtained by the addition of a crown ether (15‐crown‐5 for **1^Li^** or 12‐crown‐4 for **2^Li^**) to a suspension of [LiPPh_2_]_∞_ in toluene (Scheme 2).

While **1^Li^** is very soluble in toluene and is obtained in a yield of 78% as orange crystals by crystallization at −30 °C, **2^Li^** only dissolves by adding THF up to a ratio of 2:3 toluene:THF and can be obtained in 30% yield as pale‐yellow crystals. The lower solubility of **2^Li^** is probably due to the formation of oligomeric or polymeric species in donor‐free solvents, which break up upon addition of a polar donor solvent like THF.

**Scheme 3:**
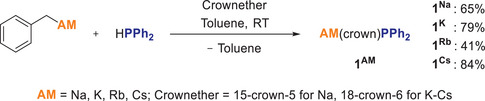
Synthesis of alkali metal phosphides **1** ^AM^ with AM = Na, K, Rb, Cs.

The heavier alkali metal phosphides were synthesized directly by deprotonating HPPh_2_ with the corresponding benzyl derivatives of the alkali metals Na‐Cs in the presence of the respective crown ether (Scheme 3). This has the advantage that the conjugate acid is toluene, in which the reaction is carried out and thus no by‐products must be separated. The products were crystallized from hot toluene solutions, which were then stored at −30 °C to further promote crystallization. This provides good to very good yields for the isolated complexes [**1^Na^** (65%), **1 ^K^** (79%), **1^Rb^** (41%), and **1^(Cs)^** (84%)]. Additionally, further material can be obtained by concentration of the solution and storage again at −30 °C, particularly in the case of **1^Rb^**.

**Scheme 4:**
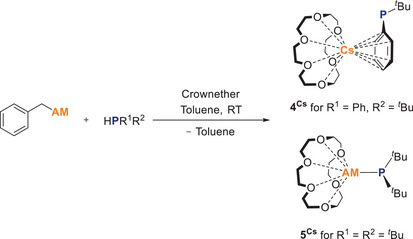
Synthesis of cesium phosphides **4** ^Cs^ and **5** ^Cs^.

In addition, the mixed aryl/alkyl and dialkyl phosphides of cesium were of interest in order to compare the bonding interactions with those of the diphenyl phosphide. For this purpose, benzyl cesium was reacted with HP*^t^* BuPh and HP*^t^* Bu_2_ respectively in the presence of 18‐crown‐6 in the same way as described above and the compounds Cs(18‐crown‐6)P*^t^* BuPh (**4^Cs^**) and Cs(18‐crown‐6)P*^t^* Bu_2_ (**5^Cs^**) were obtained (Scheme 4).

While **4^Cs^** is obtained as large red‐orange crystals in 52% yield, it was found that although **5^Cs^** crystallizes and can be investigated using SC‐XRD methods, it cannot be isolated in pure form. During crystallization, the formation of a dark brown decomposition product was observed. When the solid was dried in a vacuum, the orange crystals also decomposed. Decomposition was also observed when the toluene‐moist crystals were stored at room temperature in an argon atmosphere for a longer period of time. This is possibly due to the lower bond strength between the cesium crown ether fragment and the P*^t^* Bu_2_ which is described in more detail in the discussion of the quantum chemical bond analysis.

### 2.2 Solid State Structures

**Figure 1:**
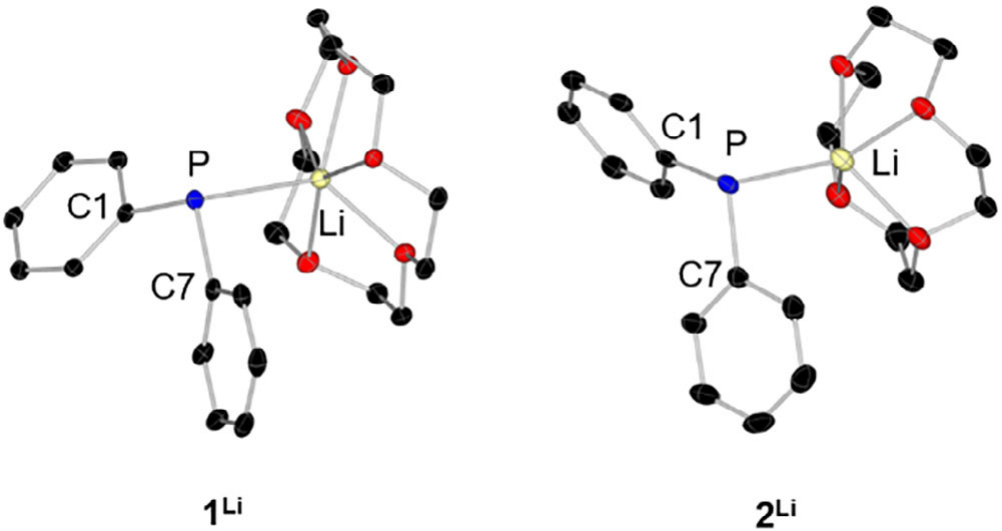
Molecular structures of **1** ^Li^ and **2** ^Li^ in the solid state. Hydrogen atoms are omitted for clarity. Thermal ellipsoids have been drawn at 50% probability.

The lithium compounds crystallize in the monoclinic space groups P2_1_/n (**1^Li^**) and P2_1_/c (**2^Li^**), each with one formula unit in the asymmetric unit. The molecular structures in the solid state are shown in Figure 1.

Five published structures for lithium diphenyl phosphide with different donors and lithium‐phosphorus contact can be found in the literature. While [Li(Et_2_O)_2_PPh_2_]_∞_ (**A**), [Li(THF)_2_PPh_2_]_∞_ (**B**), and [Li(DME)PPh_2_]_∞_ (**C**) are presented as coordination polymers, a dimeric ([Li(TMEDA)PPh_2_]_2_, **D**) and a monomeric (Li(PMDTA)PPh_2_ **E**) structure have been reported with nitrogen donors.^[^ ^18^ ^]^ With the multidentate crown ethers, **1^Li^** and **2^Li^** show monomeric structures with a Li–P distance of 2.650(2) Å (**1^Li^**) and 2.557(2) Å (**2^Li^**), respectively. The shortening of this distance by 0.1 Å in **2^Li^** compared to **1^Li^** is due to the lithium atom lying deeper in the coordination pocket of the crown ether. Compared with the distances of the known structures, comparable values were obtained (Table 2). The Li–O distances are approx. 0.15 Å longer in **1^Li^** than in **2^Li^**, which is due to the size of the crown ether pocket. If one compares the Li–O distances with those for the compound [Li(12‐c‐4)_2_][PPh_2_] (⌀ Li–O = 2.35 Å), these are in a comparable region.^[^ ^19^ ^]^

**Table 2:** Selected bond distances of **1^Li^**, **2^Li^**, and the structures **A‐E** known from literature in the solid state.

	1^Li^	2^Li^	A	B	C	D	E
Li–P / Å	2.650(2)	2.557(2)	⌀2.489	⌀2.632	2.567(6)	⌀2.614	2.552
⌀ Li–O / Å	2.276	2.115	1.946	1.963	‐	‐	1.992

**Figure 2:**
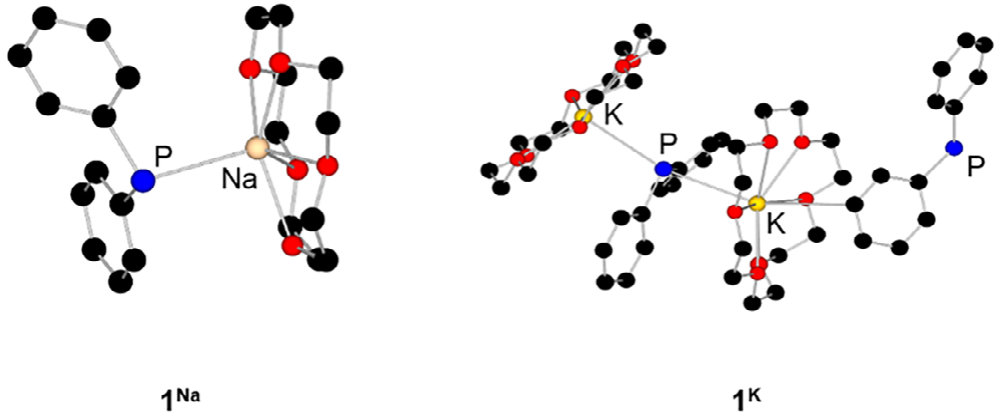
Reported molecular structures of **1** ^Na^ and **1** ^K^ in the solid state.^[^ ^11^, ^17^ ^]^ Hydrogen atoms are omitted for clarity.

The phosphorus atom in **1^Li^** is with the angles: C1–P1–Li1 = 97.27(6)°, C7–P1–C1 = 104.49(6)°, and C7–P1–Li1 = 110.48(6)° distorted pseudo‐tetrahedral coordinated and the lithium atom is distorted pentagonal pyramidal coordinated by five oxygen atoms and the phosphorus atom. A very similar picture emerges for **2^Li^** with the angles C1–P1–Li1 = 92.29(7)°, C7–P1–C1 = 104.69(6)°, and C7–P1–Li1 = 100.00(7)° whereby the angles here deviate more strongly from the ideal tetrahedral angle. The lithium atom in **2^Li^** is distorted square pyramidally coordinated by four oxygen atoms and one phosphorus atom, whereby the lithium atom is strongly deflected out of the plane of the oxygen atoms by 0.887 Å (Figure 1). The structures of **1^Na^** and **1^K^** are displayed in Figure 2 for comparison.

**Figure 3:**
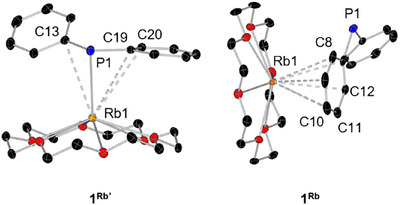
Different molecular structures of **1^Rb^** in the solid state. Hydrogen atoms are omitted for clarity. Thermal ellipsoids have been drawn at 30% probability.

For **1^Rb^**, we were able to determine two different molecular structures in the solid state using SC‐XRD methods. Both show monomeric structures (Figure 3), where only one of the two has an Rb–P contact (**1^Rb^**) and the other is *η* ^6^‐coordinated by one of the phenyl rings (**1^Rb'^**). **1^Rb^** crystallizes in the monoclinic space group P2_1_/c with two formula units and one molecule of toluene in the asymmetric unit. **1^Rb'^** crystallizes in the tetragonal space group I4 with two formula units in the asymmetric unit.

The average Rb–P distance in reported rubidium phosphides is 3.479 Å, which perfectly fits **1^Rb^**.^[^ ^20^ ^]^ The average Rb–O distances of 2.952 Å also fit very well with the average Rb–O distances of 3.05 Å reported in the literature for similar compounds.^[^ ^20^, ^21^ ^]^ The Rb–C distances in **1^Rb^** (listed in Table 3) are slightly longer compared to those in **1^Rb'^** in which the phenyl ring coordinates to the rubidium atom in a *η* ^6^ fashion. The reported rubidium–carbon distances in similar compounds vary between 3.393–3.625 Å for *η* ^3^ coordination and between 3.297–3.700 Å for *η* ^6^ coordination.^[^ ^20e^ ^]^ Similar structural diversity was reported by Ruhland‐Senge and coworkers for the crown ether coordinated rubidium diphenyl methanide Rb(18‐crown‐6)C(H)Ph_2_. Here, the different coordination modes *η* ^6^ and *η* ^3^ were observed depending on the crystallization temperature.^[^ ^22^ ^]^

**Table 3:** Selected bond distances of **1^Rb^** and **1^Rb'^** in the solid state.

	1^Rb^	1^(Rb')^
Rb–P / Å	3.4202(7)	4.5319(11)
⌀ Rb–O / Å	2.952	2.917
Rb–C / Å	C13: 3.516(3)	C7: 3.3562(18)
	C19 3.544(3)	C8: 3.4353(18)
	C20 3.632(3)	C9: 3.483(2
		C10: 3.442(2)
		C11: 3.354(2)
		C12: 3.305(2)

**Figure 4:**
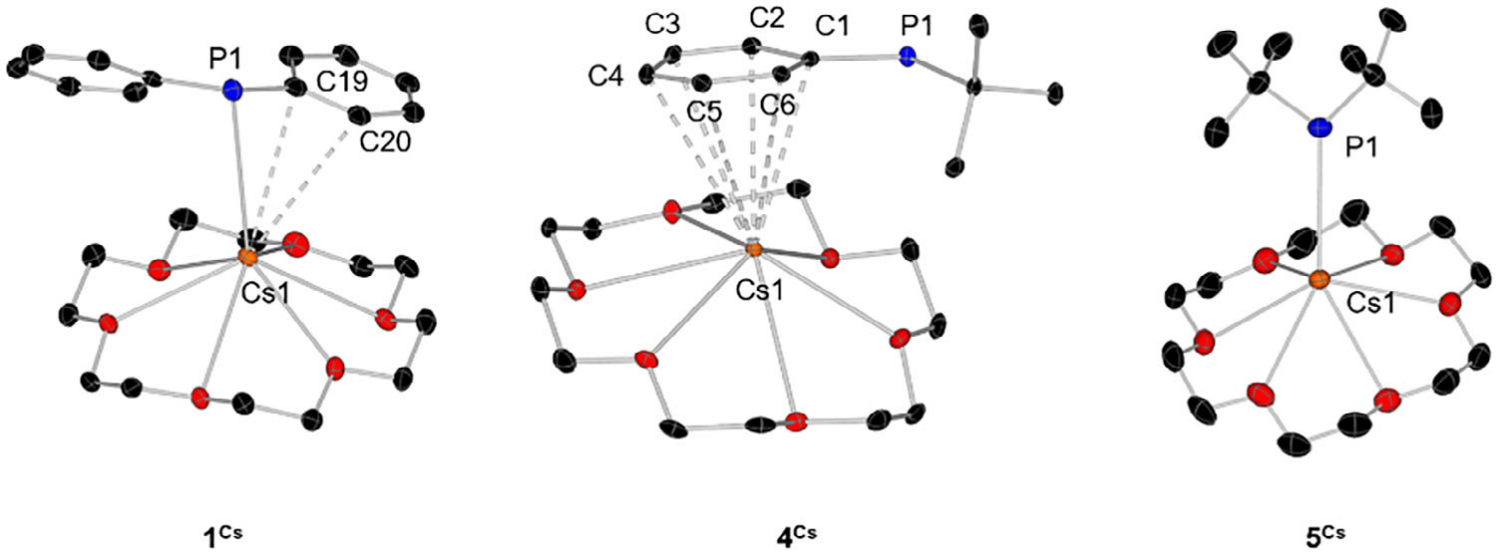
Molecular structures of **1** ^Cs^, **3** ^Cs^, and **4** ^Cs^ in the solid state. Hydrogen atoms are omitted for clarity. Thermal ellipsoids have been drawn at 30% probability.

The cesium phosphides crystallize in the monoclinic space group P2_1_ (**1^Cs^**), orthorhombic space group P2_1_2_1_2_1_ (**4^Cs^**), and the monoclinic space group P2_1_/n (**5^Cs^**) all with six (**1^Cs^**) or one (**4^Cs^** and **5^Cs^**) formula unit in the asymmetric unit (Figure 4). While in **1^Cs^** the PPh_2_ fragment coordinates with a phosphorus atom and twice *η* ^2^ with the phenyl rings to the Cs crown fragment, in **4^Cs^** only *η* ^6^ coordination of the phenyl ring is observed. In the absence of arene rings, only the phosphorus atom coordinates to the Cs‐crown fragment in **5^Cs^**. Selected bond distances are summarized in Table 4.

**Table 4:** Selected bond distances of **1^Cs^**, **4^Cs^**, and **5^Cs^** in the solid state.

	1^Cs^	4^Cs^	5^Cs^
Cs–P / Å	3.797(3)	4.2372(9)	3.4559(12)
⌀ Cs–O / Å	3.160	3.130	3.121
Cs–C / Å	C19: 3.561(11)	C1: 3.428(3)	
	C24: 3.686(12)	C2: 3.480(3)	
		C3: 3.574(4)	
		C4: 3.648(4)	
		C5: 3.639(4)	
		C6: 3.543(4)	

The Cs‐P distance in **1^Cs^** of 3.797(3) Å is within the average of published Cs–P distances in similar compounds of 3.604 Å.^[^ ^20^, ^23^ ^]^ The Cs–C distances in **1^Cs^** and **4^Cs^** are similar to those in published compounds, which are between 3.365–3.708 Å.^[^ ^20^, ^23^ ^]^ Similarly, the average Cs–O distances of the title compounds are within the range of those of published compounds (3.171 Å).^[^ ^23a^ ^]^ It is also interesting to note that in **4^Cs^** a comparatively long (4.2372(9) Å) Cs–P distance is observed and the cesium is preferentially coordinated via *π*‐arene interactions rather than by direct contact with the donor atom. Similar structural motifs have been described several times in the literature, in which the cesium atom favours arene coordination over donor atom coordination.^[^ ^23^, ^24^ ^]^ In **5^Cs^** a significantly (0.34 Å) shorter Cs–P distance is observed compared to that of **1^Cs^**. This can be explained by the lack of aryl interaction and the increased nucleophilicity of the dialkyl phosphide fragment.

For the angles C–P–C between the phenyl groups and the phosphorus atom, no significant deviation between the different alkali metals is observed (Table 5, entry 2). The angles between each phenyl group, the phosphorus atom and the alkali metal show that very similar structural motifs are obtained for Li and Na, with potassium representing an outlier, as only the dimeric structure has been reported. For rubidium and cesium, these two angles show a significantly smaller value, as the PPh_2_ fragment “slides upwards” and additionally coordinates to the alkali metal centre via *π*‐arene interactions.

**Table 5:** Selected bond angles of **1^AM^** in the solid state.

	1^Li^	1^Na^	1^K^	1^Rb^	1^Cs^
C–P–AM / °	97.27(6)	95.49(5)	120.37(7)	30.48(5)	68.6(4)
C–P–AM / °	110.48(6)	105.78(5)	98.60(7)	30.09(5)	88.1(3)
C–P–C / °	104.49(6)	105.67(7)	107.13(10)	107.18(15)	106.4(5)

**Figure 5:**
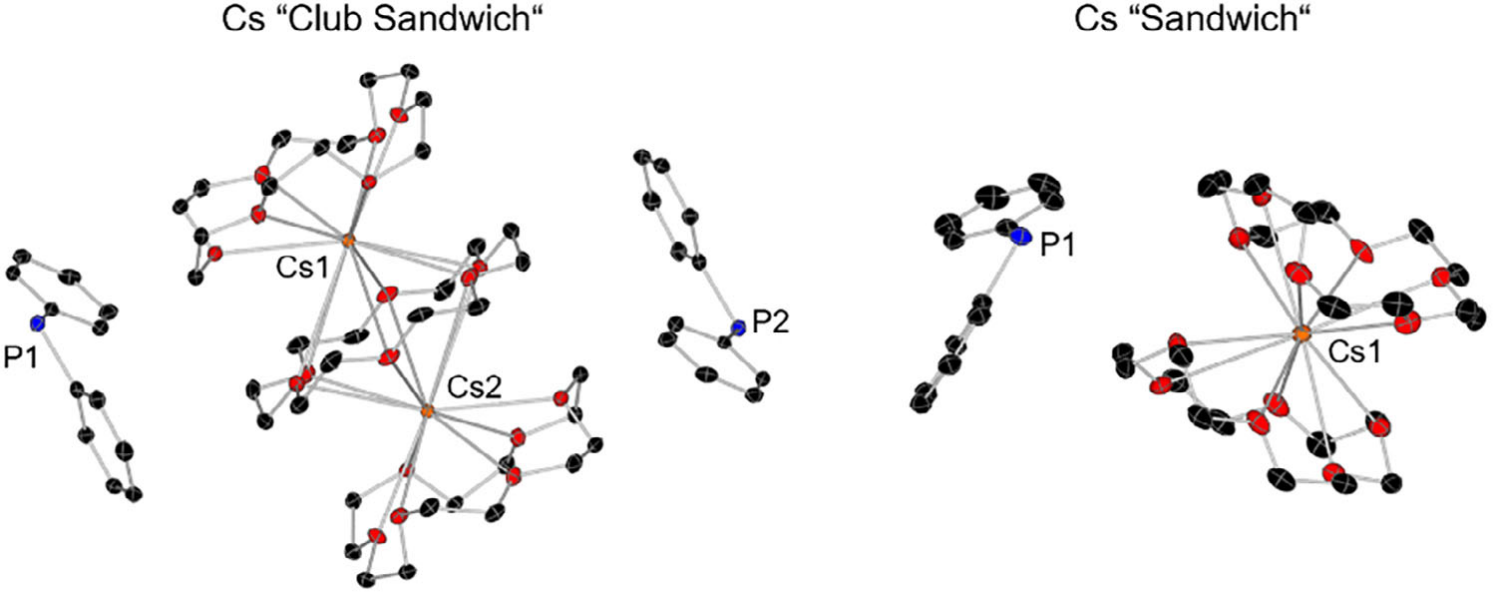
Molecular structures of cesium sandwich structures in the solid state. Hydrogen atoms are omitted for clarity. Thermal ellipsoids have been drawn at 30% probability.

Further, we were able to obtain two more cesium phosphide structures. During the synthesis of **1^Cs^**, more than one equivalent of 18‐crown‐6 was added to the reaction mixture and after crystallization, we noticed that different types of crystals were found. Besides the main part, which could be identified as **1^Cs^**, we were able to obtain two sandwich structures [Cs_2_(18‐c‐6)_3_][PPh_2_]_2_ (“club sandwich”) and Cs(18‐c‐6)_2_PPh_2_ (“sandwich”) (Figure 5). Further attempts to selectively synthesize these two compounds failed and they could only be identified using SC‐XRD methods. The average Cs–O distances of 3.314 and 3.35 Å are approx. 0.2 Å longer than those of the above‐mentioned and literature‐known compounds, which is not surprising due to the coordination of two crown ethers. A compound isostructural to the described “club sandwich” structure was described for the cesium diphenyl methanide [Cs_2_(18‐c‐6)_3_][C(H)Ph_2_]_2_.^[^ ^25^ ^]^

### 2.3 Solution Behavior

**Figure 6:**
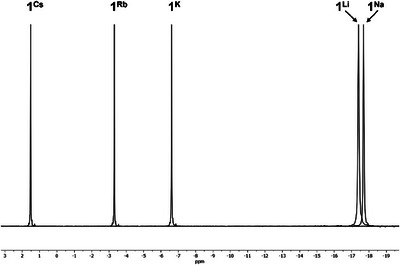
Superimposed ^31^P{^1^H} NMR spectra of **1** ^AM^ in C_6_D_6_ at 300 K.

The title compounds were analyzed in C_6_D_6_ solution using ^1^H and ^31^P{^1^H} NMR spectroscopy. **2^Li^** will not be discussed in detail, here as **2^Li^** does not dissolve in C_6_D_6_ like the other compounds. The ^1^H NMR spectra are very similar, with a characteristic signal for the coordinated crown ether being detected at *δ*_1H_ (ppm) = 3.15 (**1^Li^**), 3.08 (**1^Na^**), 3.12 (**1 ^K^**), 3.11 (**1^Rb^**), 3.13 (**1^Cs^**), 3.14 (**4^Cs^**), and 3.31 (**5^Cs^**). The only significant deviation here is **5^Cs^**, which is due to the stronger interaction between the cesium atom and the phosphide donor. Furthermore, for **1^AM^** a set of signals consisting of three multiples between 6–8.25 ppm is detected for the aromatic protons of the PPh_2_ units. For **4^Cs^** and **5^Cs^**, a doublet is detected for the *^t^* Bu group at *δ*_1H_ (ppm) = 1.97 (^3^ *J*_PH_ = 9.58 Hz) and 2.13 (^3^ *J*_PH_ = 8.80 Hz), respectively. While **1^Li^** (−17.4 ppm) and **1^Na^** (−17.7 ppm) show very similar shifts in the ^31^P{^1^H} spectrum, a clear trend toward low‐field shifted resonances *δ*_31P_ (ppm) = −6.6 (**1 ^K^**), −3.3 (**1^Rb^**), and 1.5 (**1^Cs^**) can be observed for the heavy alkali metal phosphides (Figure 6). Due to the alkyl substituent on the phosphide, the resonances for **4^Cs^** (39.9 ppm) and **5^Cs^** (90.1 ppm) are clearly downfield shifted compared to **1^Cs^**.

In order to investigate the behavior of alkali metal phosphides in solution in more detail, diffusion ordered spectroscopy (DOSY) NMR methods were applied to determine the diffusion constants (D) for the title compounds.^[^ ^26^ ^]^ Diffusion measurements on ionic transition metal compounds provide information on the interaction between cations and anions and thus on the degree of ion pair formation in solution.^[^ ^27^ ^]^ We applied this principle to the series of alkali metal phosphides **1^AM^** presented and the results are summarized in Table 6. In addition, the corresponding hydrodynamic radii r_H_ were calculated according to the Stokes‐Einstein equation with a specific viscosity for C_6_D_6_ of *η* = 0.4047·10^−3 ^kg m^2^ s^−2^. The values for r_Hc_ and r_Ha_ hardly differ from each other and increase with increasing weight of the alkali metal, which is in line with expectations. Dialkyl phosphide **5^Cs^** displays an exception where the two values deviate from each other by 12%, which is an indication that there is a certain degree of separated ions in solution. This is supported by the value 1.13 for the quotient of the diffusion coefficients. The degree of ion pairing can be determined using the quotient of the cationic (Dc) and anionic (Da) diffusion coefficients. A value of Dc/Da = 1 indicates a bond between anionic and cationic fragments, a deviation from the unit value is an indication of a certain degree of solvent‐separated ion pairs (SSIPs). For **1^AM^** and **4^Cs^** the quotients are almost one which, together with the values for the hydrodynamic radii, strongly indicates molecular compounds with cation‐anion interactions.

**Table 6:** DOSY NMR (c = 30 mM, 300 K, C_6_D_6_) data of the title compounds **1^AM^**, **4^Cs^**, and **5^Cs^**. Measured diffusion coefficients Da and Dc for anion and cation, calculated hydrodynamic radii r_Ha_ and r_Hc_ for anion and cation, calculated, and experimentally determined molecular weights using the diffusion coefficient for the cationic fragment Dc.

	⌀ Da / ^−10^m^2^/s	Dc / ^−10^m^2^/s	Dc/Da	r_Ha_ / Å	r_Hc_ / Å	MW_exp_ / g/mol	MW_calc_ / g/mol	MW_diff_
**1^Li^**	6.19	6.52	1.05	8.78	8.33	464	412	−11%
**1^Na^**	5.87	5.88	1.00	9.26	9.23	548	428	−22%
**1^K^**	5.87	5.71	0.97	9.26	9.51	595	503	−15%
**1^Rb^**	5.48	5.55	1.01	9.92	9.78	612	549	−10%
**1^Cs^**	4.93	4.87	0.99	11.02	11.15	749	596	−20%
**4^Cs^**	5.68	5.78	1.02	9.56	9.39	553	556	1%
**5^Cs^**	6.73	7.63	1.13	8.07	7.12	367	576	57%

In addition, the molar masses MW_exp_ of **1^AM^** were determined using the methods presented by Stalke and coworkers (Table 6).^[^ ^28^ ^]^ This allows us to determine the possible formation of aggregates in solution. For the compounds **1^AM^** and **4^Cs^**, the values for the experimentally determined molar masses are a maximum of 22% higher than the theoretically calculated values, which indicates the presence of monomeric species, but suggests a dynamic process is operative and formation of higher aggregates. The deviation of 57% for **5^Cs^** supports the previous assumptions that there are at least partially SSIPs present the in solution. This behavior in solution possibly reflects the significantly lower stability of **5^Cs^** compared to the other alkali metal phosphides.

To investigate whether it is possible to generate the sandwich and club sandwich structures in solution, we added one and two equivalents of 18‐crown‐6 to a solution of **1^Cs^** and analyzed the solution by ^31^P{^1^H} and ^133^Cs NMR spectroscopy. While the signals of *δ*_133Cs_ (ppm) = 53.4 (**1^Cs^**), 46.0 (**1^Cs^ **+ 18‐crown‐6), and 40.7 (**1^Cs^ **+ 2 eq. 18‐crown‐6) in the ^133^Cs NMR spectrum shifts by approx. 6 ppm, the resonance in the ^31^P{^1^H} NMR remains almost identical with a change of *Δδ*_31P_ = 0.2 ppm, indicating the sole presence of **1^Cs^** in solution at room temperature (Supporting Information Figures S27, S28). The shift of the signal in the ^133^Cs NMR is most likely due to the change in polarity of the sample. Variable temperature NMR studies have not been applied yet but will be a valuable tool in future investigations of such compounds.

### 2.4 Quantum Chemical Bond Analysis

**Figure 7:**
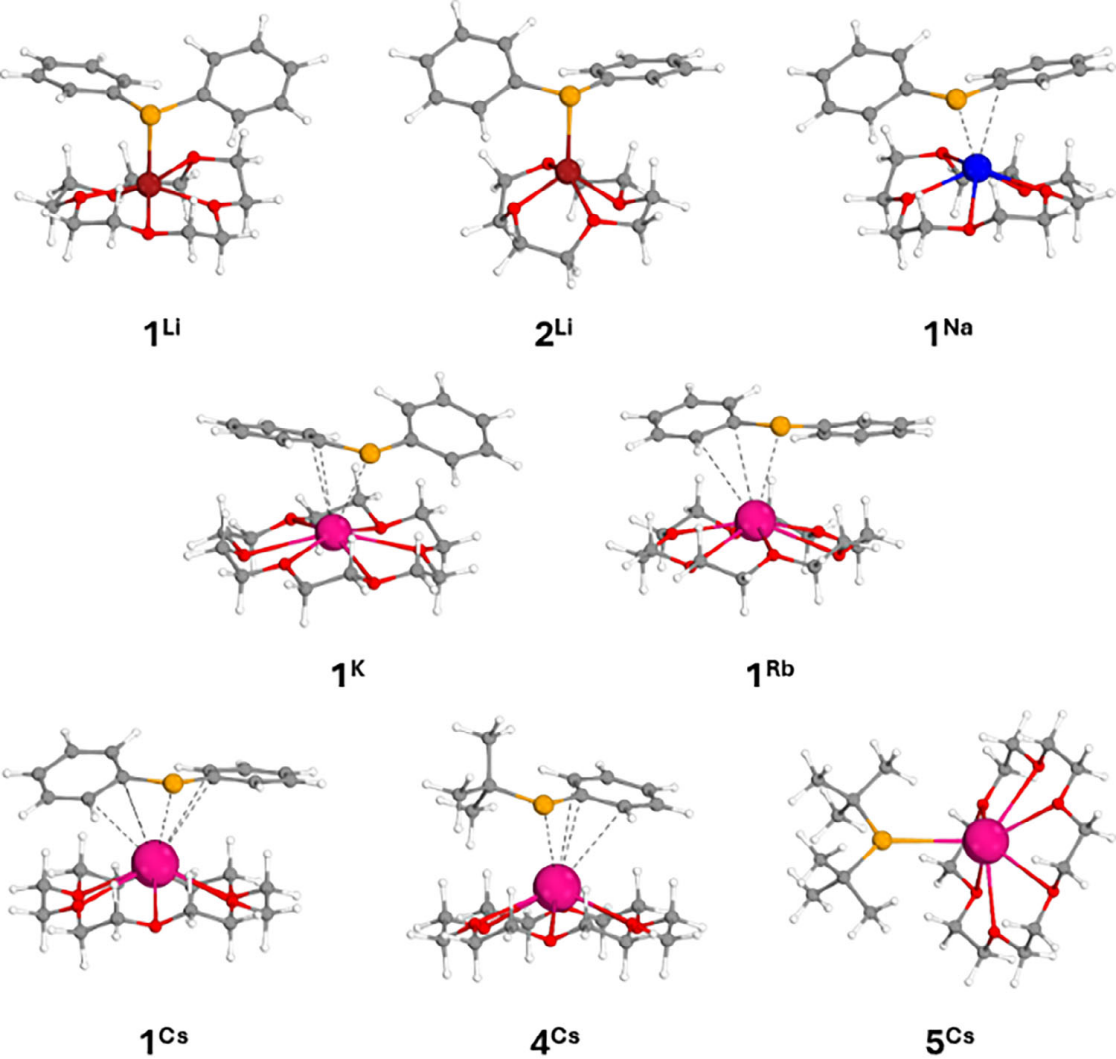
Calculated (RI‐BP86‐D3BJ/def2‐SVP) minimum structures of the alkali metal phosphides **1** ^AM^, **2** ^Li^, **4** ^Cs^, and **5** ^Cs^.

**Figure 8:**
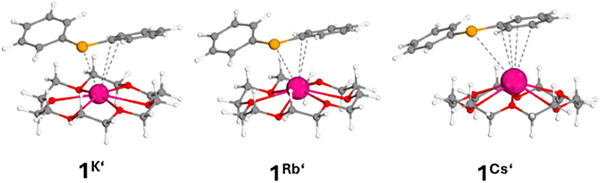
Calculated (BP86‐D3BJ/def2‐SVP) minimum structures of the second isomers of the alkali metal phosphides **1** ^K'^, **1** ^Rb'^, and **1** ^Cs'^.

As can be clearly seen in Figure 7, the minimum structures calculated at the dispersion‐corrected (RI)‐BP86‐D3BJ/def2‐SVP level are very similar to those in the solid state. Descending the group, we found that the coordination to the phosphorus atom has less and less weight and the favored *π*‐arene interactions are well represented. Since we observed different coordination modes of the phenyl groups in the solid‐state structures, we calculated the corresponding second isomers of the heavy AM phosphides **1^K'^**, **1^Rb'^**, and **1^Cs'^** (Figure 8). The energy differences are very small with 0.2 (K), 1.0 (Rb), and 1.7 (Cs) kcal/mol. Interestingly, no minimum structure with *η* ^6^ coordination of the phenyl ring, as in the solid state, was found for rubidium, whereas the second isomer for cesium is now only coordinated by a phenyl ring *η* ^4^.

**Figure 9:**
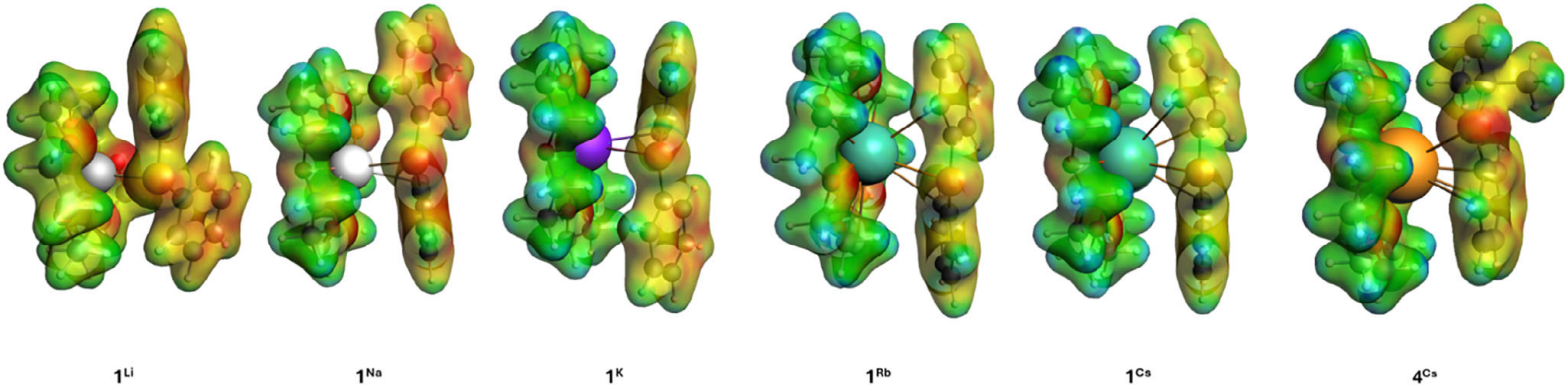
Molecular electrostatic potential (MEP) plots of alkali metal phosphides **1** ^AM^. Red stands for areas of high electron density and blue for areas of low electron density.

To gain a more detailed insight into the bonding situation in the newly presented and the two known alkali metal phosphides, we performed a natural bond orbital analysis (NBO) calculated at the BP86‐D3BJ/def2‐SVP level. The results are summarized in Table 7, and the Wiberg Bond Indices (WBI) clearly show that the interaction between the alkali metal and the phosphorus atom is very weak. As expected, the strongest interaction is observed in **5^Cs^**. It is also interesting to note that the NBO charge *q* on the alkali metal is close to +1, but the phosphorus atom appears almost uncharged, indicating a delocalization of the charge via the aromatic system of the phenyl rings. This is well illustrated by the molecular electrostatic potential (MEP) plots in Figure 9, were red displays regions of increased electron density and blue regions of low electron density.

**Table 7:** Results of the NBO analysis (BP86‐D3BJ/def2‐SVP) for **1^AM^**, **2^Li^**, **4^Cs^**, and **5^Cs^**.

NBO	1^Li^	2^Li^	1^Na^	1^K^	1^Rb^	1^Cs^	4^Cs^	5^Cs^
**WBI (AM‐P)**	0.01	0.15	0.06	0.03	0.05	0.03	0.06	0.16
***q*(AM)**	0.80	0.78	0.82	0.85	0.84	0.84	0.84	0.79
***q*(P)**	0.06	0.07	0.12	0.16	0.17	0.18	0.08	−0.19

**Figure 10:**
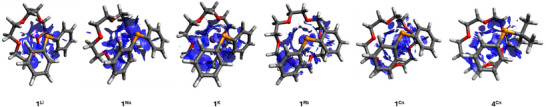
Plots (isosurface = 0.6 a.u.) of the noncovalent interactions (shown as blue surfaces) in the alkali metal phosphides **1** ^AM^.

A much more detailed insight into the bonding situation between the crown ether alkali metal fragment and the phosphide was obtained by applying an energy decomposition analysis (EDA) calculated at the ZORA‐BP86‐D3BJ/DZP level using the optimized geometries at the (RI)‐BP86‐D3BJ/def2‐SVP level. From the data in Table 8, it becomes clear that the bond between [AM(crown)]^+^ and [PR_2_]^−^ is dominated by electrostatic interactions (measured by the term Δ*V*_elstat_), which contribute 77–81% to the total interaction (Δ*E*_int_). Nevertheless, both the orbital (19–21%) and dispersion (12–23%) interactions, albeit comparatively weaker, are not negligible. It is interesting to note that the total interaction (Δ*E*_int_) steadily decreases by descending in group 1 and ultimately also decreases from diaryl phosphide **1^Cs^** to dialkyl phosphide **5^Cs^**. The latter may further underline the instability of compound **5^Cs^** observed experimentally. Finally, to visualize the noncovalent interactions between the [AM(crown)]^+^ and [PR_2_]^−^, which are responsible for the Δ*E*_disp_ term, the NCI plots of the alkali metal phosphides **1^AM^** and **4^Cs^** are shown in Figure 10, indicating the occurrence of multiple stabilizing CH···π interactions.

**Table 8:** Results of the EDA‐NOCV analysis (BP86‐D3BJ/def2‐SVP) for **1^AM^**, **2^Li^**, **4^Cs^**, and **5^Cs^**.

EDA‐NOCV	1^Li^	2^Li^	1^Na^	1^K^	1^Rb^	1^Cs^	4^Cs^	5^Cs^
***ΔE*_int_**	−106.6	−105.3	−103.3	−97.5	−95.2	−93.8	−93.8	−90.5
***ΔE*_Pauli_**	30.3	39.5	34.5	34.7	30.7	31.3	31.5	27.2
***ΔV*_elstat_**	−94.3	−96.2	−93.5	−88.0	−84.2	−84.8	−85.7	−81.6
***ΔE*_orb_**	−25.2	−25.5	−22.5	−21.7	−20.1	−22.7	−23.3	−24.1
***ΔE*_disp_**	−17.4	−23.1	−21.8	−22.5	−21.5	−17.6	−16.3	−11.9
**Elstat / %**	79	79	81	80	81	79	79	77
**Orb / %**	21	21	19	20	19	21	21	23

## 3 Conclusion

In conclusion, we herein presented the synthesis and full characterization of the two lithium diphenyl phosphides Li(15‐crown‐5)PPh_2_ (**1^Li^**) and Li(12‐crown‐4)PPh_2_ (**2^Li^**) as well as the heavy homologues Rb(18‐crown‐6)PPh_2_ (**1^Rb^**) and Cs(18‐crown‐6)PPh_2_ (**1^Cs^**). We thus completed the series of crown ether coordinated alkali metal diphenyl phosphides and showed that the structural diversity increases as descending the group. While the lithium derivatives have a clear alkali metal‐phosphorus interaction in the solid state and form 1:1 complexes like the sodium phosphide **1^Na^**, the heavy homologues Rb and Cs tend to interact with the phosphide fragment via *π‐*arene interactions.  We were able to characterize two different structural motifs for **1^Rb^** using SC‐XRD methods, one of which has an Rb–P interaction with three additional Rb–C interactions. The second shows a very long Rb–P distance, with the diphenyl phosphide unit *η* ^6^ coordinating to the rubidium atom via a phenyl ring. For cesium, we identified three structural motifs, incorporating several equivalents of 18‐crown‐6 and yielding the Cs(18‐crown‐6)PPh_2_ (**1^Cs^**), the “sandwich”, and “club‐sandwich” structures Cs(18‐c‐6)_2_PPh_2_ and [Cs_2_(18‐c‐6)_3_][PPh_2_]_2_. However, this appears to be a phenomenon in the solid state, as these structures could not be generated in solution.

Including the literature known Na and K derivatives, we presented a detailed study of the title compounds in solution and showed that all of them are monomers possessing an alkali metal−phosphide interaction. We further investigated the influence of the substituents on the phosphorus atom on the bonding between the Cs(18‐crown‐6) and the phosphide fragment. For this purpose, we synthesized and characterized the cesium phosphides Cs(18‐crown‐6)P*^t^* BuPh (**4^Cs^**) and Cs(18‐crown‐6)P*^t^* Bu_2_ (**5^Cs^**). As observed for **1^Rb^**, **4^Cs^** shows only *η* ^6^ coordination and a very long Cs–P distance, whereas **5^Cs^** shows a relatively short Cs–P distance due to the absence of any aromatic groups. It was interesting to observe here that the heavy alkali metals Rb and Cs favor coordination via *π*‐arene interactions over the P–donor interaction. Finally, we used state‐of‐the‐art quantum chemical methods such as NBO analysis followed by EDA calculations to precisely analyze the bonding situation in the title compounds. A clear trend in the bonding energy between the alkali metal and the phosphide fragment was found. As the metal becomes heavier, the binding energy decreases, with electrostatic interactions dominating the bonding contributions in all cases.

## 4 Experimental Section

### Computational details

All calculations have been performed with the ORCA 5.0.3 or ORCA6.0.1 program.^[^ ^29^ ^]^ Geometry optimizations were carried out using the functional BP86^[^ ^30^ ^]^ and the basis set def2‐SVP^[^ ^31^ ^]^ including D3BJ dispersion correction^[^ ^32^ ^]^ and the SMD CPCM(benzene) solvent model^[^ ^33^ ^]^ as implemented in the ORCA Programme. All species were also characterized by frequency calculations, and have positive definite Hessian matrices thus confirming that the computed structures are minima on the potential energy surface. The interaction between the alkali metal crown and the phosphide fragment has been investigated with the NBO^[^ ^34^ ^]^ method using Gaussian09^[^ ^35^ ^]^ and the EDA^[^ ^36^ ^]^ at the ZORA^[^ ^37^ ^]^‐BP86‐D3BJ/DZP^[^ ^38^ ^]^ level with the ADF2024.104 software^[^ ^39^ ^]^ using the optimized RI‐BP86‐D3BJ/def2‐SVP geometries. Noncovalent interactions were visualized by means of the NCIPlot program.^[^ ^40^ ^]^

### Crystallographic details

Crystallographic data for all compounds were measured with a Rigaku Synergy‐i instrument with monochromated Cu − Kα (*λ* = 1.54 184 Å) radiation. The measured data was processed with the CrysAlisPro software package. The structures were solved in OLEX2 1.5^[^ ^41^ ^]^ by dual‐space direct methods with SHELXT,^[^ ^42^ ^]^ followed by full‐matrix least‐squares refinement to converge using SHELXL.^[^ ^43^ ^]^ All nonhydrogen atoms were refined anisotropically. The contribution of the hydrogen atoms, in their calculated positions, was included in the refinement using riding models. Reflection data for **1^Cs^** was treated as twinned by a 180 ° rotation about reciprocal 100 and refined against a hklf 5 formatted reflection file. The twin scale factor refined to 0.4774(16). Throughout, disordered groups were modelled across two sites with appropriate restraints and constraints added so as to approximate normal geometric and displacement parameters. Groups treated in this way were one of the six crystallographically independent crown ether ligands of **1^Cs^**, part of one crown ether ligand in **1^Cs^** (sandwich), and one butyl anion of **5^C^** ^s^. Structure **1^Rb^** contained a small amount of disordered and unidentified solvent. The effects of this were removed using the SQUEEZE routine as implemented in PLATON. A total of 10 electron equivalents were removed from approximately 50 Å^3^ of unit cell space. A summary of crystallographic parameters is given in the Supporting Information and a full listing of atomic coordinates, bond lengths, angles, displacement parameters, and reflection data for all the structures has been deposited at the Cambridge Crystallographic Data Centre with reference numbers CCDC 2467228 to 2467236.

### General synthetic details

All synthetic procedures were carried out under a dry nitrogen atmosphere (N_2_) using standard Schlenk techniques or in a glove box under an argon atmosphere (Ar). Before use, the glassware was predried in an oven at 150 °C and then heated with a heat gun under vacuum. The solvents were dried, distilled, and degassed using standard methods. C_6_D_6_ was dried over potassium, distilled, degassed, and then stored in the glove box over activated molecular sieves (4 Å). n‐Hexane, THF and toluene were dried in a Solvent Purification System (Innovative Technology, PS‐Micro), degassed and stored under inert atmosphere over activated 4 Å molecular sieves. Butyllithium solutions, crown ether, CsF, RbF, NaO*^t^* Bu, KO*^t^* Bu, PhPCl_2_, PPh_3_, and lithium were obtained from commercial sources and used as received. LiAlH_4_ was purified by extraction with Et_2_O, filtration, and drying in high vacuum to obtain a colorless powder. The literature known compounds Ph_2_PH,^[^ ^44^ ^]^ *^t^* Bu_2_PH,^[^ ^45^ ^]^ and BnAM^[^ ^46^ ^]^ were prepared according to published procedures. ^1^H, ^13^C{^1^H}, ^31^P{^1^H}, ^7^Li, and ^133^Cs as well as COSY, HSQC, and HMBC NMR spectra were recorded using an AV300, AV400 or AV500 MHz spectrometer. The chemical shifts (*δ* in ppm) in the ^1^H and ^13^C NMR spectra were referenced to the residual signals of the deuterated solvents. ^1^H and ^13^C{^1^H} chemical shifts were reported against Me_4_Si and ^31^P{^1^H} against H_3_PO_4_. Common abbreviations were used to describe the signal multiplications: s (singlet), d (doublet), t (triplet), q (quartet), dd (doublet of a doublet), m (multiplet), and b (broad).

### Synthesis of PhtBuPH

PhPCl_2_ (10.00 g, 7.58 mL, 56 mmol, 1 eq.) was added to 80 mL of hexane and cooled to ‐78 °C. *^t^* BuLi (1.7 M in pentane, 36 mL, 61.5 mmol, 1.1 eq.) was added dropwise via syringe. The colorless suspension was allowed to reach room temperature and was cooled again to 0 °C. Freshly purified LiAlH_4_ (636 mg, 16.8 mmol, 0.3 eq.) in 20 mL THF was added dropwise via syringe and the mixture was allowed to reach room temperature again. After stirring overnight, the volume of the mixture was reduced to approximately 50% under reduced pressure and it was filtered via cannula filtration. Removing the solvent under reduced pressure yielded a colorless oily liquid with some precipitate. After trap‐to‐trap condensation at 60 °C in dynamic vacuum the product was obtained as a colorless liquid (4.93 g, 53%). ^1^H NMR (400 MHz, 300 K, C_6_D_6_, ppm): *δ* = 7.43 – 7.35 (m, H_Ar_ 2H), 7.11 – 7.06 (m, H_Ar_, 3H), 3.93 (d, ^1^ *J_PH_* = 206.7 Hz, P*H* 1H), 1.02 (d, ^3^ *J_PH_* = 12.2 Hz, H*_t_*_Bu_, 9H). ^13^C{^1^H} NMR (100 MHz, 300 K, C_6_D_6_, ppm): *δ* = 135.54 (d, *J_PC_* = 15.3 Hz, C_Ar_), 128.09 (d, *J_PC_* = 14.9 Hz, C_Ar_), 29.45 (d, ^3^ *J_PC_* = 13.2 Hz, C*_t_*_Bu_).* ^31^P NMR (121 MHz, 300 K, C_6_D_6_, ppm): *δ* = −5.7 (dm, ^1^ *J_PH_* = 206.7 Hz). * C_ipso_ could not be identified in the ^13^C{^1^H} NMR spectrum due to low intensity.

### Synthesis of LiPPh_2_

Ph_2_PH (3.21 g, 3 mL, 17.2 mmol, 1 eq.) was added to 20 ml of pentane and cooled to 0 °C. *^n^* BuLi (2.5 M in hexane, 7.6 mL, 19.0 mmol, 1.1 eq.) was added dropwise via syringe. The bright yellow suspension was allowed to warm to room temperature, stirred for 30 minutes and filtered via cannula filtration. After washing the remaining bright yellow solids three times with hexane it was dried in high vacuum and used as obtained.

### Synthesis of 1^Li^

To LiPPh_2_ (150 mg, 781 µmol, 1 eq.) toluene (10 mL) and 15‐crown‐5 (154.9 µl, 781 µmol, 1 eq.) was added, resulting in a bright orange solution. The reaction mixture was stored at ‐30 °C overnight whereupon the product crystallizes at orange block‐shaped crystals. The mother liquor was removed and the crystals were dried in high vacuum yielding **1^Li^** as an orange crystalline material (250 mg, 606 µmol, 78%). ^1^H NMR (400 MHz, 300 K, C_6_D_6_, ppm): *δ* = 8.05 (m, H_Ph_ortho_, 4H), 7.20 (m, H_Ph_meta_, 4H), 6.91 (m, H_Ph_para_, 2H), 3.15 (s, H_crown_, 20H). ^13^C{^1^H} NMR (100 MHz, 300 K, C_6_D_6_, ppm): *δ* = 156.7 (d, ^1^ *J_CP_* = 44.2 Hz, C_Ph_ispo_), 130.2 (d, ^2^ *J_CP_* = 17.2 Hz, C_Ph_ortho_), 127.0 (d, ^3^ *J_CP_* = 5.7 Hz, C_Ph_meta_), 119.0 (s, C_Ph_para_), 68.4 (s, C_crown_). ^31^P{^1^H} NMR (121 MHz, 300 K, C_6_D_6_, ppm): *δ* = −17.4 (s). ^7^Li NMR (155 MHz, 300 K, C_6_D_6_, ppm): *δ* = ‐0.36 (bs).

### Synthesis of 2^Li^

LiPPh_2_ (100 mg, 520 µmol, 1 eq.) toluene (10 mL) and 12‐crown‐4 (183.4 µL, 1 mmol, 2 eq.) were combined in a Schlenk flask, resulting in a bright orange suspension. After adding THF (15 mL) the volume of the orange solution was reduced to half under reduced pressure. The product crystallizes from this mixture at room temperature in the form of bright yellow needles. The mother liquor was removed, the crystals were washed 3 times with pentane and dried in high vacuum yielding **2^Li^** as yellow crystalline material (60 mg, 163 µmol, 31%). Further product can be obtained by cooling the combined mother liquor and pentane wash to ‐30 °C. ^1^H NMR (400 MHz, 300 K, THF‐d_8_, ppm): *δ* = 7.40 (m, H_Ph_ortho_, 4H), 6.74 (br, H_Ph_meta_, 4H), 6.43 (br, H_Ph_para_, 2H), 3.67 (s, H_crown_, 20H). ^13^C{^1^H} NMR (100 MHz, 300 K, THF‐d_8_, ppm): *δ* = 128.8 (br, C_Ph_ortho_), 126.4 (br, C_Ph_meta_), 117.5 (s, C_Ph_para_), 68.6 (s, C_crown_).* ^31^P{^1^H} NMR (121 MHz, 300 K, THF‐d_8_, ppm): *δ* = ‐11.4 (bs). ^7^Li NMR (155 MHz, 300 K, THF‐d_8_, ppm): *δ* = 0.00 (bs). * C_ipso_ could not be identified in the ^13^C{^1^H} NMR spectrum due to low intensity.

### General synthesis of AM(crown)PPh_2_ (AM = Na, K, Rb, Cs)

BnAM precursor (1.3 mmol, 1 eq., see Table 9) was suspended in toluene (20 mL) and crown ether (1.3 mmol, 1 eq., see Table 9) was added, followed by Ph_2_PH (1.3 mmol, 226.2 µL 1 eq.) resulting in an orange suspension. After heating to reflux until all solids dissolved deep orange solutions formed from which the products began to crystallize at room temperature. Storing at ‐30 °C overnight, removing the mother liquor and drying the crystals in high vacuum yielded **1^Na^** (365 mg, 65%), **1 ^K^** (500 mg, 79%), **1^Rb^** (286 mg, 41%), and **1^Cs^** (655 mg, 84%) as orange crystalline material. The obtained analytical data for **1^Na^** and **1 ^K^** matches the previously reported data.^[^ ^11^, ^17^ ^]^ **1^Rb^**: ^1^H NMR (400 MHz, 300 K, C_6_D_6_, ppm): *δ* = 8.11 (m, H_Ph_ortho_, 4H), 7.13 (m, H_Ph_meta_, 4H; overlap with C_6_D_6_), 6.78 (m, H_Ph_para_, 2H), 3.11 (s, H_crown_, 24H). ^13^C{^1^H} NMR (100 MHz, 300 K, C_6_D_6_, ppm): *δ* = 159.7 (d, ^1^ *J_CP_* = 57.8 Hz, C_Ph_ispo_), 128.7 (d, ^2^ *J_CP_* = 19.4 Hz, C_Ph_ortho_), 127.5 (overlap with C_6_D_6_, C_Ph_meta_), 117.3 (s, C_Ph_para_), 69.6 (s, C_crown_). ^31^P{^1^H} NMR (121 MHz, 300 K, C_6_D_6_, ppm): *δ* = ‐3.2 (s). **1^Cs^**: ^1^H NMR (400 MHz, 300 K, C_6_D_6_, ppm): *δ* = 8.14 (m, H_Ph_ortho_, 4H), 7.18 (m, H_Ph_meta_, 4H, overlap with C_6_D_6_), 6.80 (m, H_Ph_para_, 2H), 3.14 (s, H_crown_, 24H). ^13^C{^1^H} NMR (100 MHz, 300 K, C_6_D_6_, ppm): *δ* = 158.8 (d, ^1^ *J_CP_* = 55.5 Hz, C_Ph_ispo_), 128.9 (d, ^2^ *J_CP_* = 18.9 Hz, C_Ph_ortho_), 127.3 (d, ^3^ *J_CP_* = 5.6 Hz, C_Ph_meta_), 117.6 (s, C_Ph_para_), 69.7 (s, C_crown_). ^31^P{^1^H} NMR (121 MHz, 300 K, C_6_D_6_, ppm): *δ* = 1.5 (s). ^133^Cs NMR (65 MHz, 300 K, 0.1 M C_6_D_6_, ppm): *δ* = 53.4 (bs).

**Table 9:** Quantities for the synthesis of **1^AM^**.

	BnAM	Crown
**1^Na^**	BnNa (148 mg)	15‐crown‐5 (257.3 mL)
**1^K^**	BnK (169 mg)	18‐crown‐6 (343 mg)
**1^Rb^**	BnRb (230 mg)	18‐crown‐6 (343 mg)
**1^Cs^**	BnCs (300 mg)	18‐crown‐6 (353 mg)

Synthesis of **4^Cs^**: BnCs (100 mg, 446 µmol, 1 eq) was placed in toluene (5 ml) and 18‐crown‐6 (118 mg, 446 µmol, 1 eq) was added, followed by Ph_2_PH (80.2 µl, 446 µmol, 1 eq) resulting in a dark orange suspension. After gentle heating, a red solution formed from which the products began to crystallize at room temperature. Storing at ‐30 °C overnight, removing the mother liquor and drying the crystals in high vacuum yielded **4^Cs^** (130 mg, 52%) as dark orange crystalline material. ^1^H NMR (400 MHz, 300 K, C_6_D_6_, ppm): *δ* = 7.49 (m, H_Ph_ortho_, 2H), 7.00 (m, H_Ph_meta_, 2H), 6.38 (m, H_Ph_para_, 1H), 3.15 (s, H_crown_, 24H), 1.98 (d, ^3^ *J_PH_* = 9.6 Hz, H*_t_*_Bu_, 19H). ^13^C{^1^H} NMR (100 MHz, 300 K, C_6_D_6_, ppm): *δ* = 126.3 (d, ^2^ *J_CP_* = 20.8 Hz, C_Ph_ortho_), 127.4 (C_Ph_meta_, overlap with C_6_D_6_), 110.3 (d, ^4^ *J_CP_* = 4.3 Hz C_Ph_para_), 69.7 (s, C_crown_), 33.1 (d, ^2^ *J_PH_* = 14.4 Hz, C*_t_*_Bu_). ^31^P{^1^H} NMR (121 MHz, 300 K, C_6_D_6_, ppm): *δ* = 39.8 (s). ^133^Cs NMR (65 MHz, 300 K, 0.1 M C_6_D_6_, ppm): *δ* = 37.0 (bs).

Synthesis of **5^Cs^**: BnCs precursor (76 mg, 340 µmol, 1 eq) was placed in toluene (5 ml) and 18‐crown‐6 (90 mg, 340 µmol, 1 eq) was added, followed by *^t^* Bu_2_PH (64.5 µl, 340 µmol, 1 eq) resulting in a dark red solution. The volume was reduced to approx. 1 ml under reduced pressure and the red oily residue was layered with hexane 10 ml. Removing the mother liquor and drying the crystals in high vacuum yielded **5^Cs^** (114 mg, 62%) as dark orange crystalline material with some unknown impurities. **5^Cs^** decomposes when stored in solution or as a solid under an argon atmosphere. ^1^H NMR (400 MHz, 300 K, C_6_D_6_, ppm): *δ* = 3.28 (s, H_crown_, 24H), 2.13 (d, ^3^ *J_PH_* = 8.8 Hz, H*_t_*_Bu_, 18H). ^13^C{^1^H} NMR (100 MHz, 300 K, C_6_D_6_, ppm): *δ* = 70.4 (s, C_crown_), 38.1 (d, ^2^ *J_PH_* = 15.2 Hz, C*_t_*_Bu_). ^31^P{^1^H} NMR (121 MHz, 300 K, C_6_D_6_, ppm): *δ* = 90.1 (s).

## Conflict of Interest

The authors declare no conflict of interest.

## Supporting information



Supporting Information

Supporting Information

## Data Availability

Deposition numbers 2467228 to 2467236 contain the supplementary crystallographic data for this paper. These data are provided free of charge by the joint Cambridge Crystallographic Data Centre and Fachinformationszentrum Karlsruhe Access Structures service. The data that support the findings of this study are openly available in Pureportal.strath.ac.uk at reference number https://doi.org/10.15129/a38f6a15‐7c42‐4ba6‐a3f8‐1b525d9e9ae7, reference number 291 460 728.
