# Supplementary material for: Crown Ether Supported Alkali Metal Phosphides: Synthesis, Structures and Bonding
Source: Chemistry. 2025 Aug 3;31(51):e02127. doi: 10.1002/chem.202502127 (PMC12434438; doi:10.1002/chem.202502127)
Supplement: Supplementary file 1 — Supporting Information [file CHEM-31-e02127-s002.docx]

Supporting Information

TOC

[Section S1 – NMR Spectra 2](#_Toc204000606)

[Section S2 – DOSY NMR studies 16](#_Toc204000607)

[Section S3 – Crystallographic details 17](#_Toc204000608)

[Section S4 – XYZ Files of the Optimized Structures 19](#_Toc204000609)

# Section S1 – NMR Spectra

Figure S1. ^1^H NMR spectrum of ^t^BuPhPH in C_6_D_6_ at 300 K.


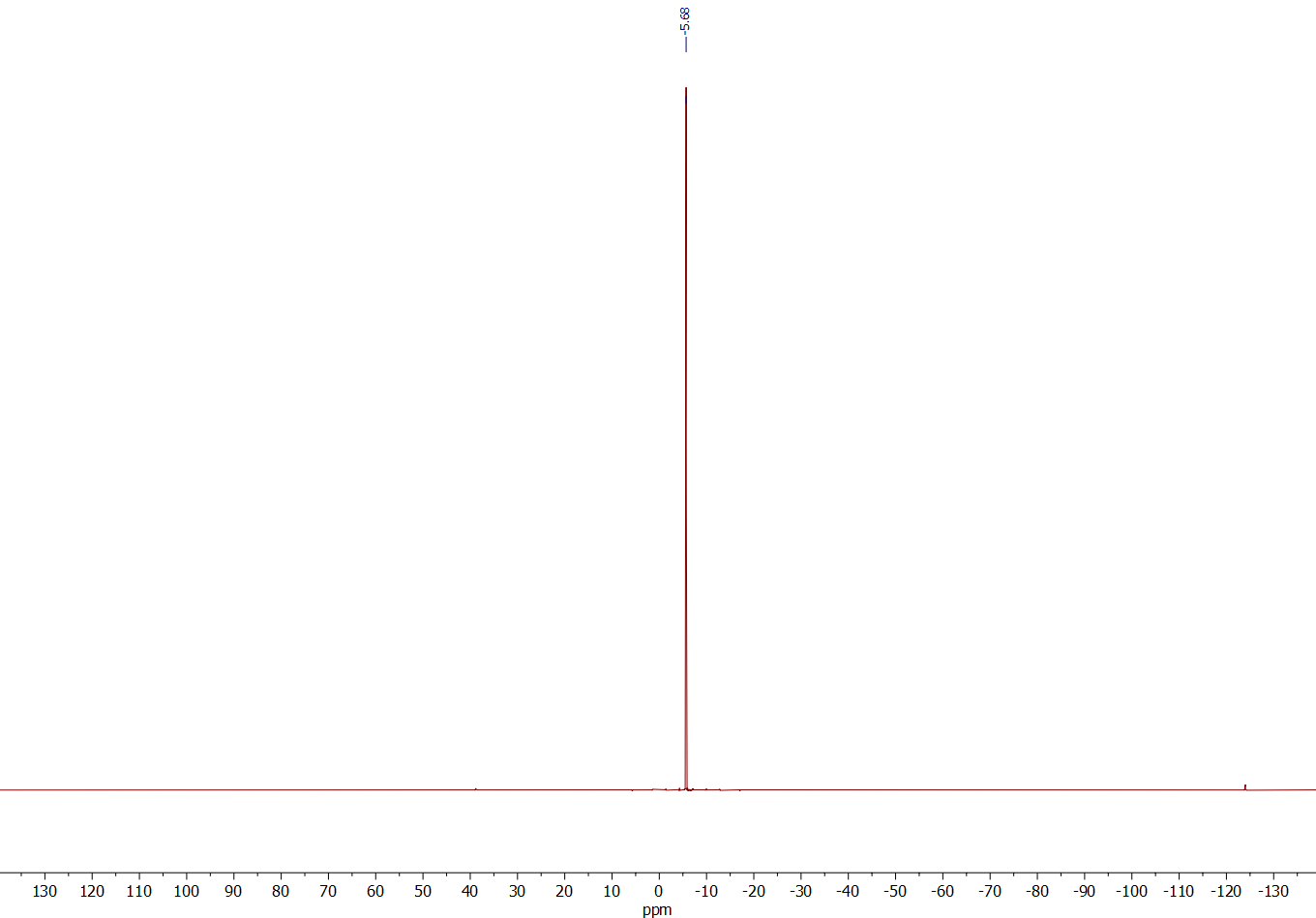


Figure S2. ^31^P{^1^H} NMR spectrum of ^t^BuPhPH in C_6_D_6_ at 300 K.


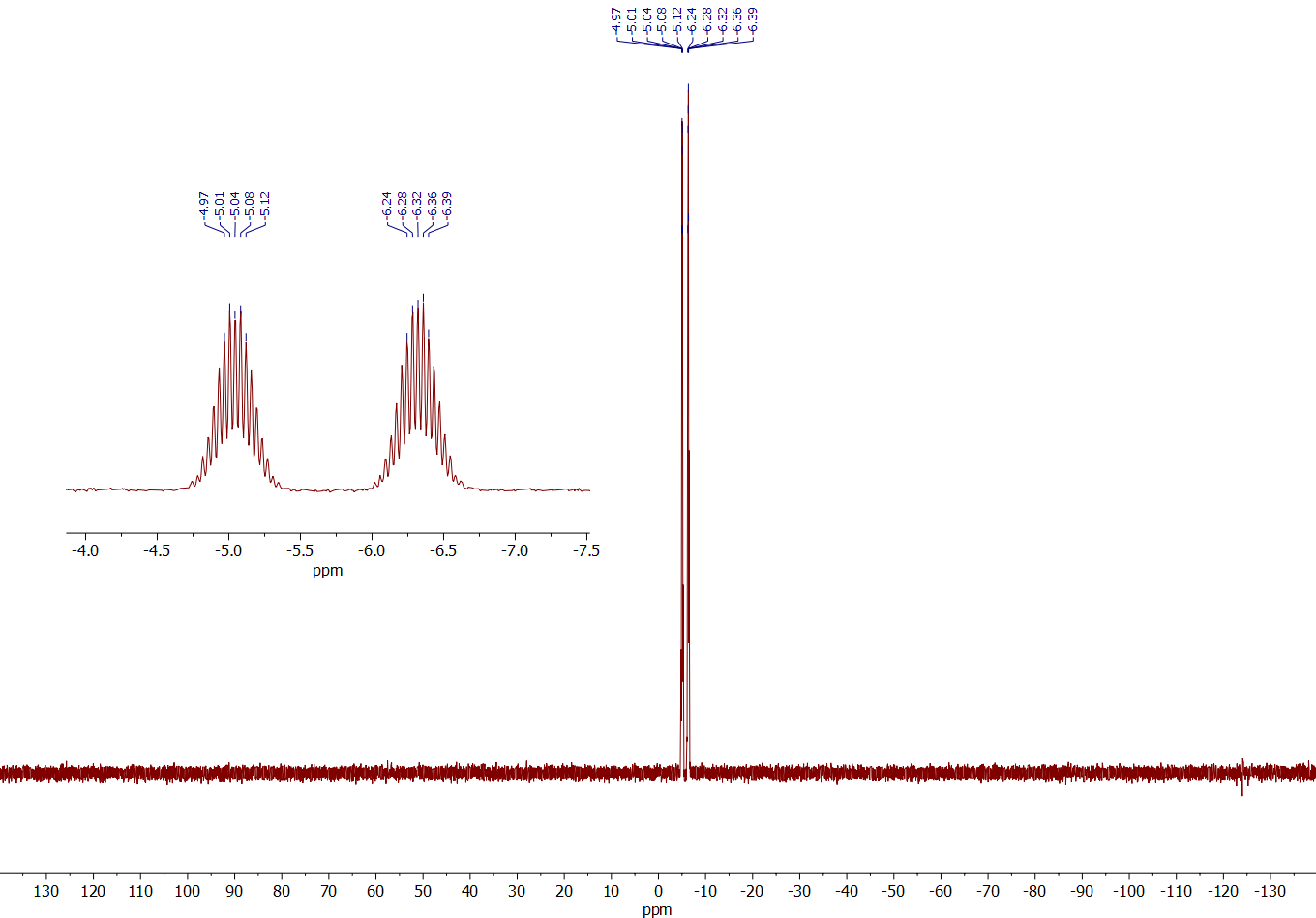


Figure S3. ^31^P NMR spectrum of ^t^BuPhPH in C_6_D_6_ at 300 K.


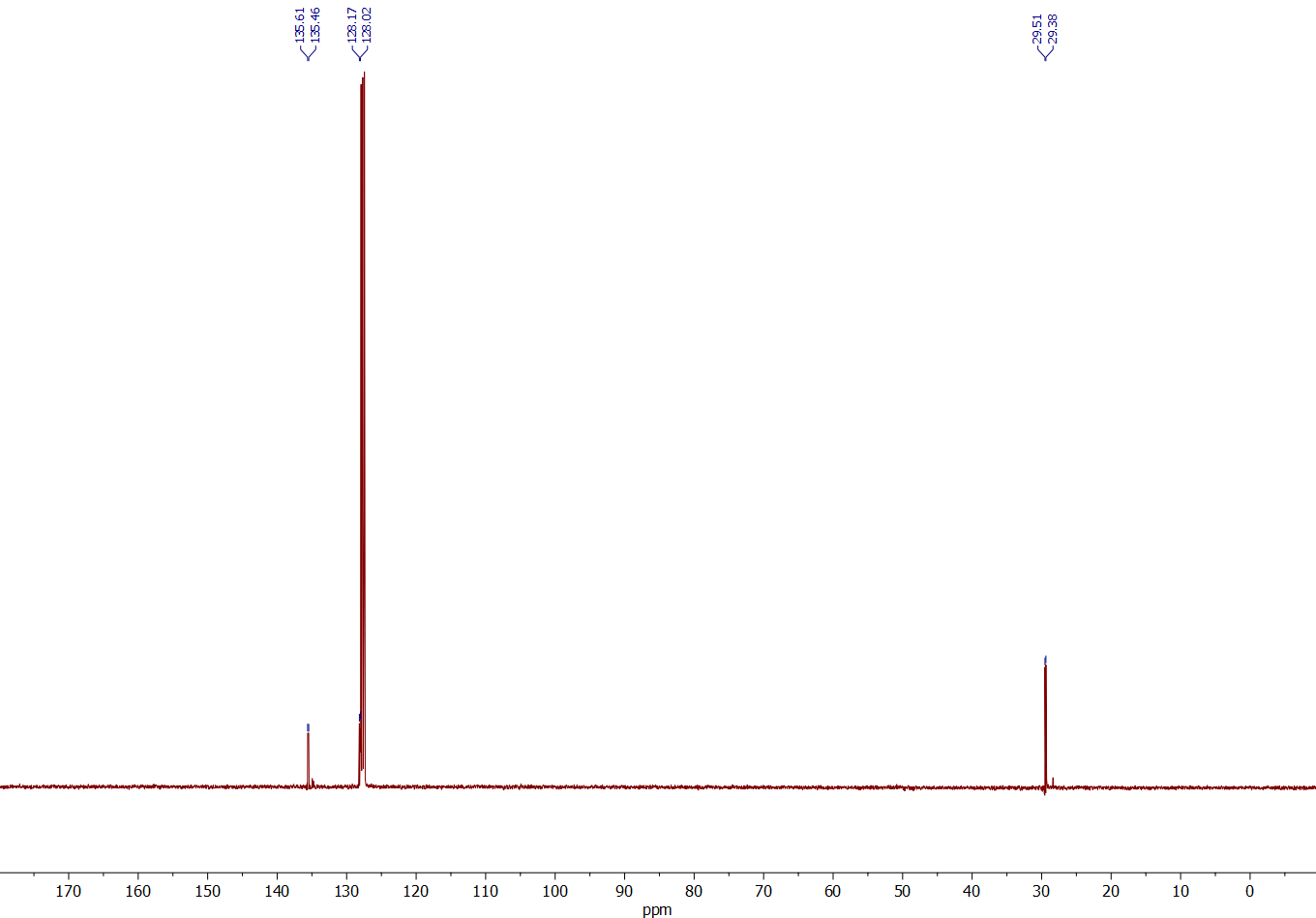


Figure S4. ^13^C NMR spectrum of ^t^BuPhPH in C_6_D_6_ at 300 K.

Figure S5. ^1^H NMR spectrum of **1^Li^** in C_6_D_6_ at 300 K.


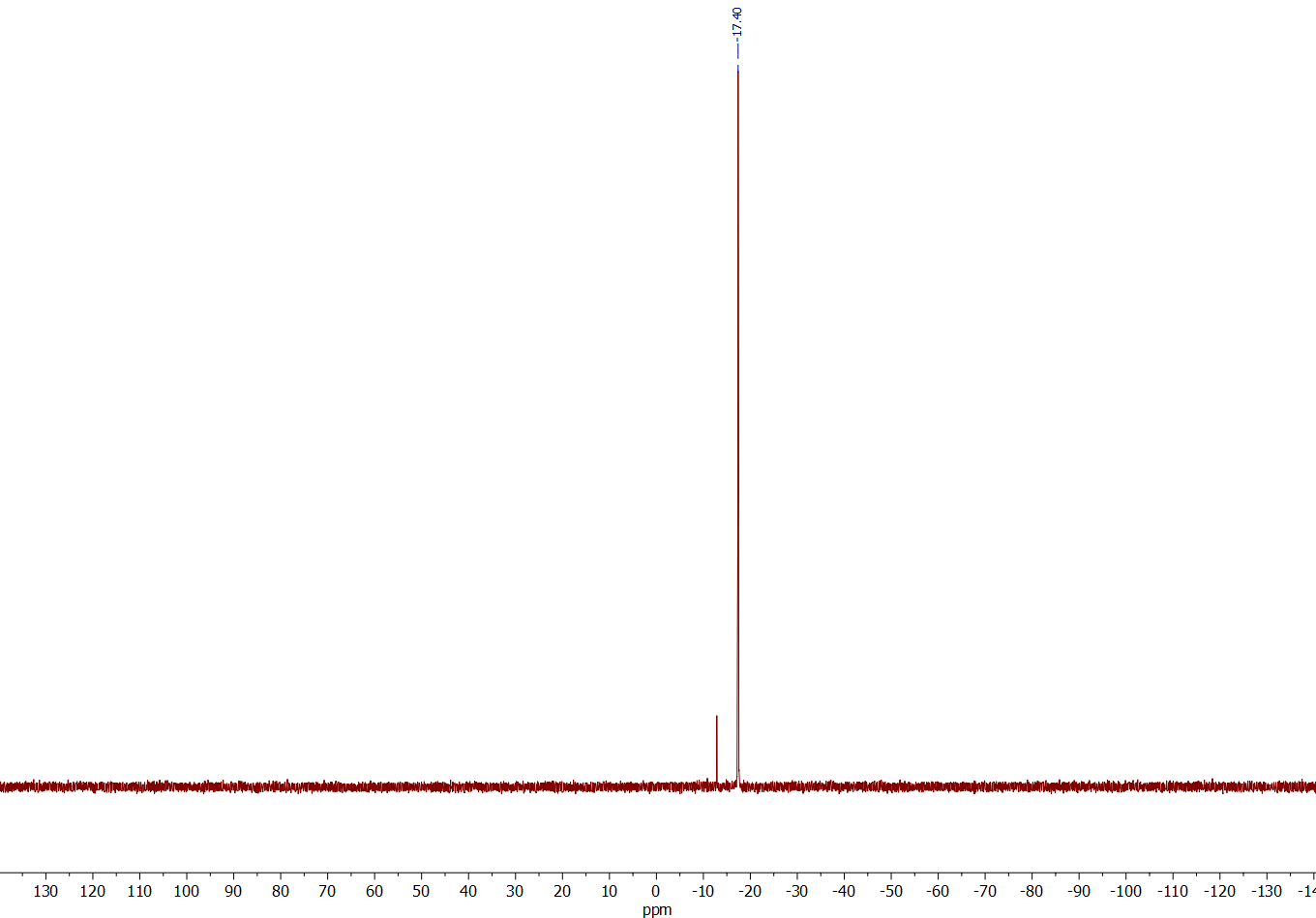


Figure S6. ^31^P{^1^H} NMR spectrum of **1^Li^** in C_6_D_6_ at 300 K. Signal at -10 ppm belongs to an unknown impurity.


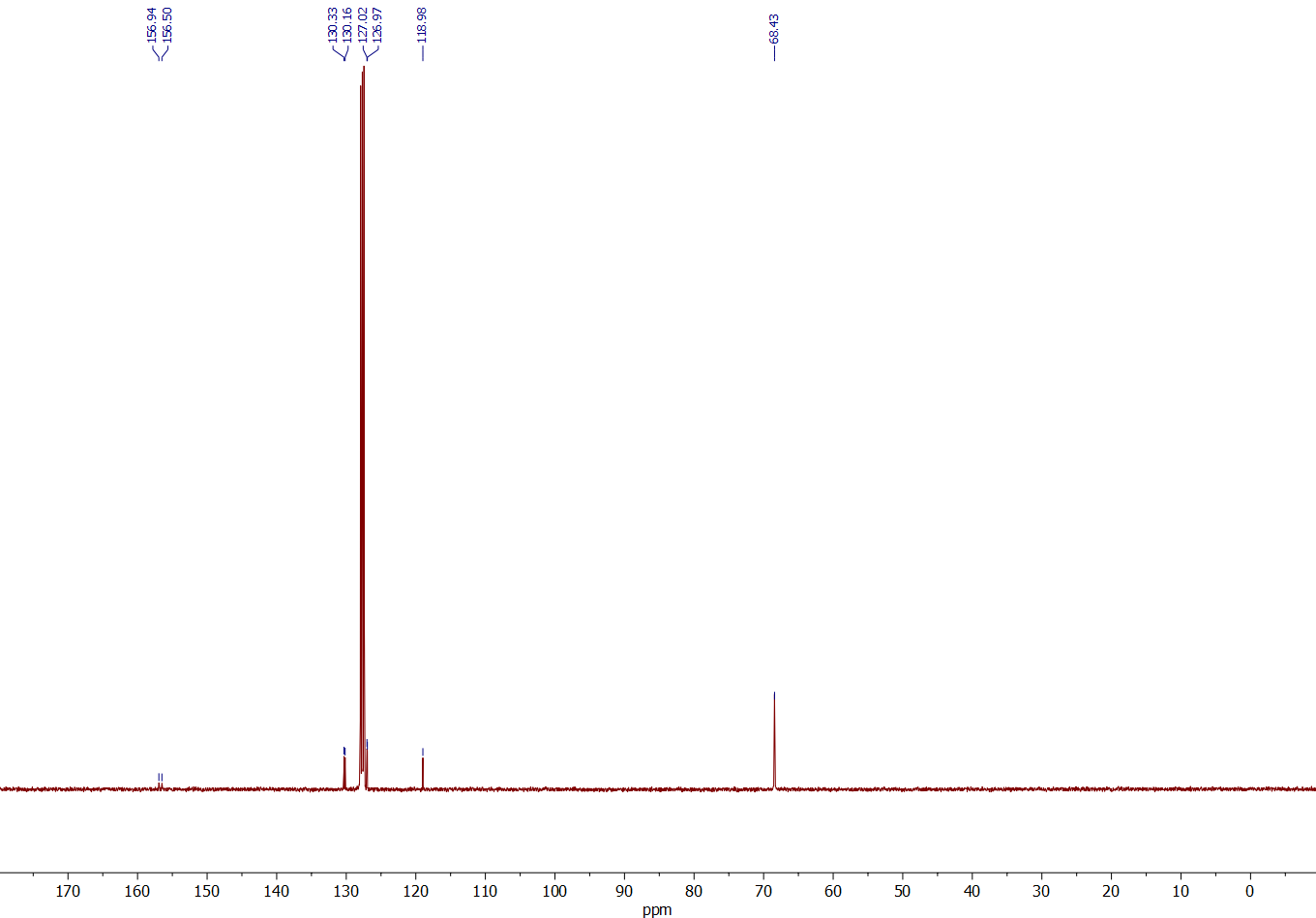


Figure S7. ^13^C NMR spectrum of **1^Li^** in C_6_D_6_ at 300 K.


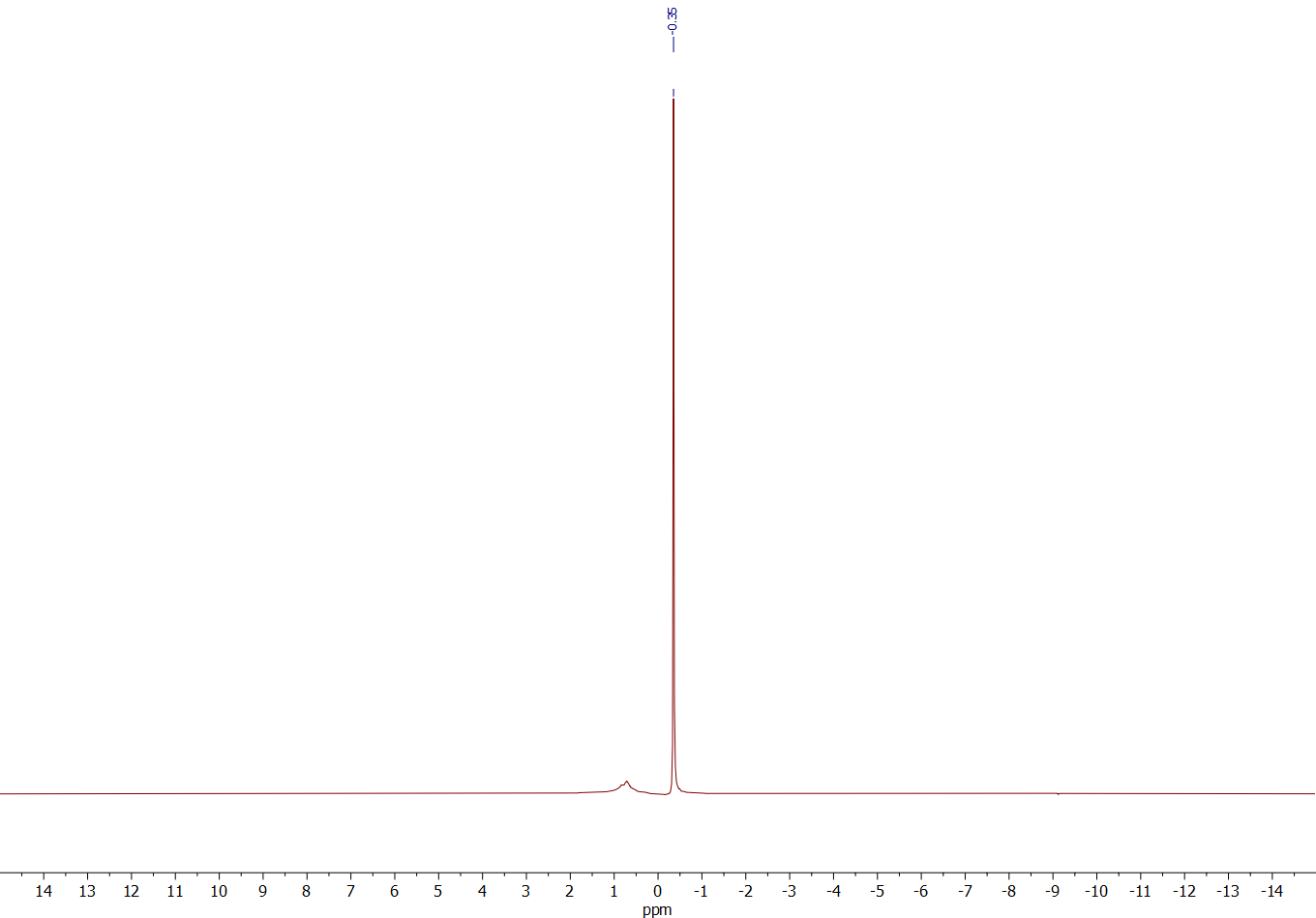


Figure S8. ^7^Li NMR spectrum of **1^Li^** in C_6_D_6_ at 300 K. Signal at 1 ppm belongs to an unknown impurity.

Figure S9. ^1^H NMR spectrum of **2^Li^** in THF-D_8_ at 300 K.


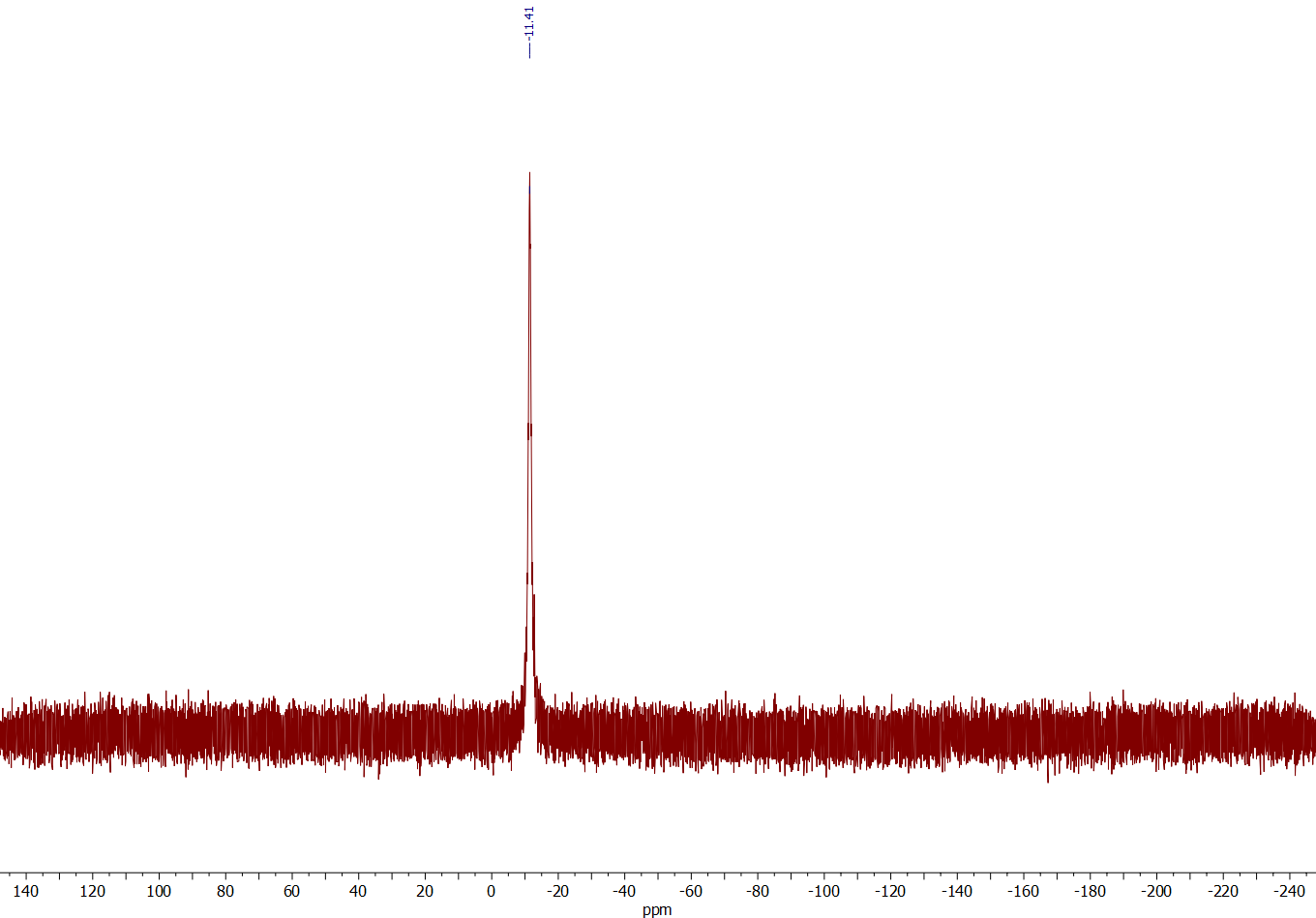


Figure S10. ^31^P{^1^H} NMR spectrum of **2^Li^** in THF-D_8_ at 300 K.


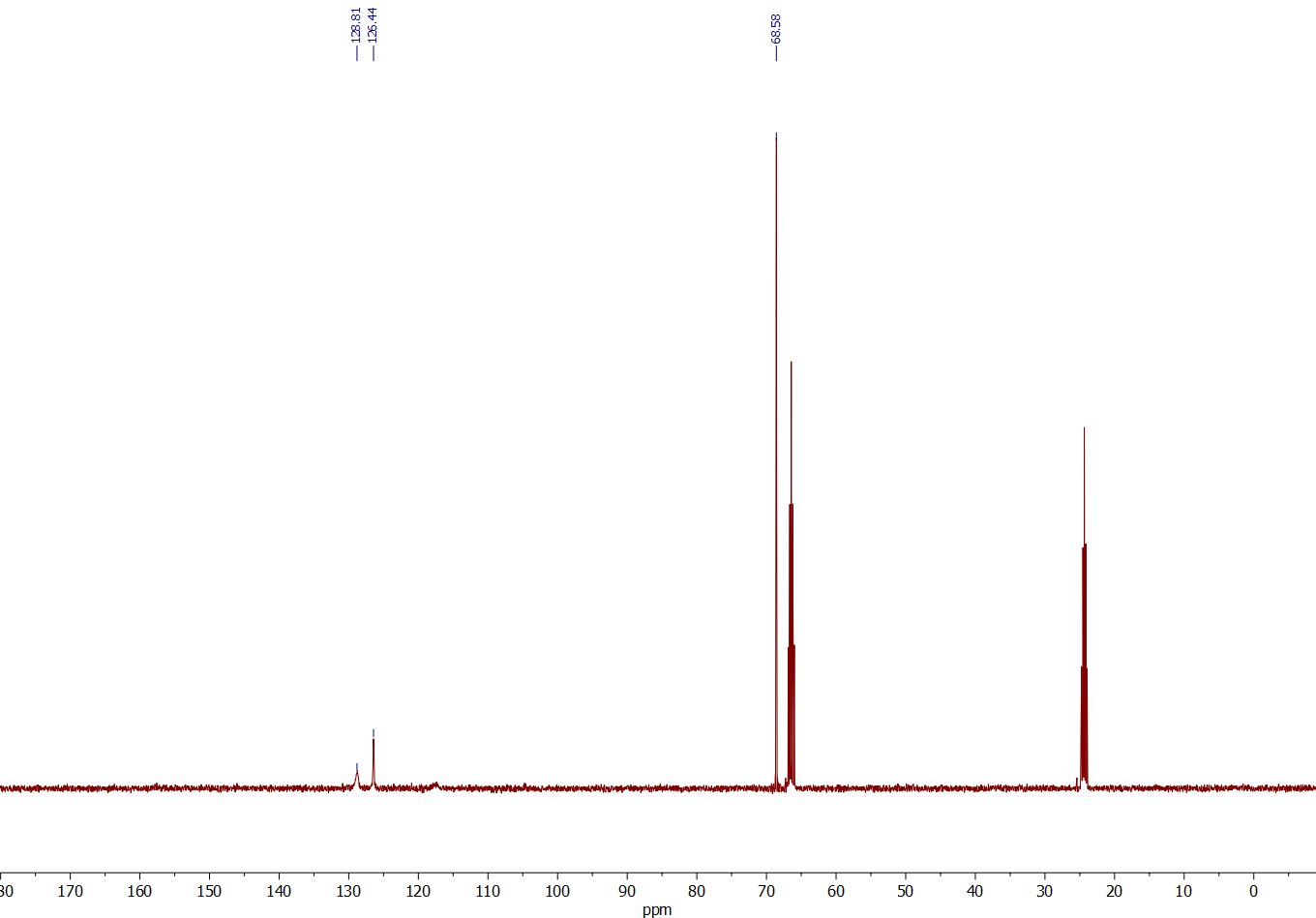


Figure S11. ^13^C NMR spectrum of **2^Li^** in THF-D_8_ at 300 K.


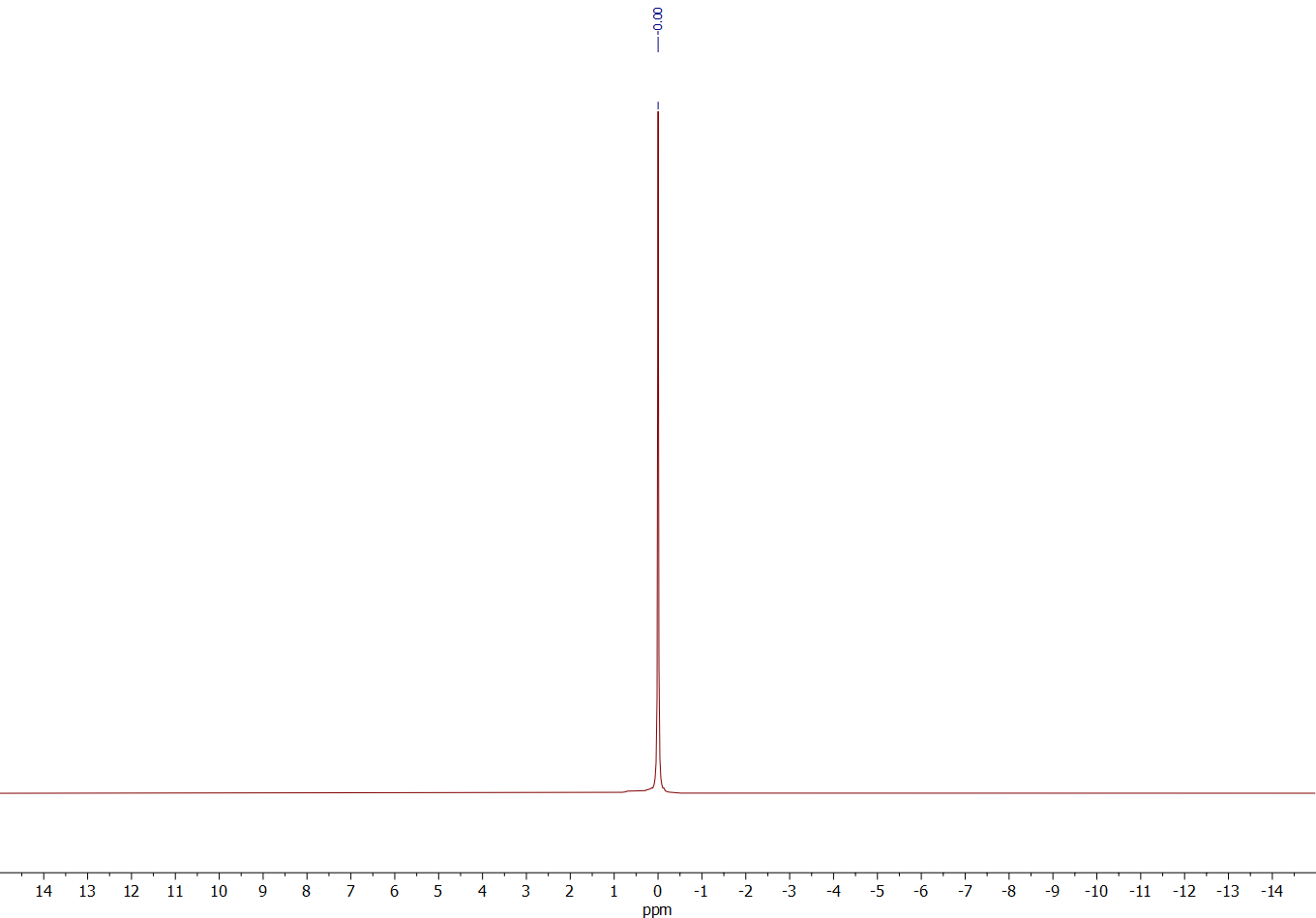


Figure S12. ^7^Li NMR spectrum of **2^Li^** in THF-D_8_ at 300 K.

Figure S13. ^1^H NMR spectrum of **1^Rb^** in C_6_D_6_ at 300 K.


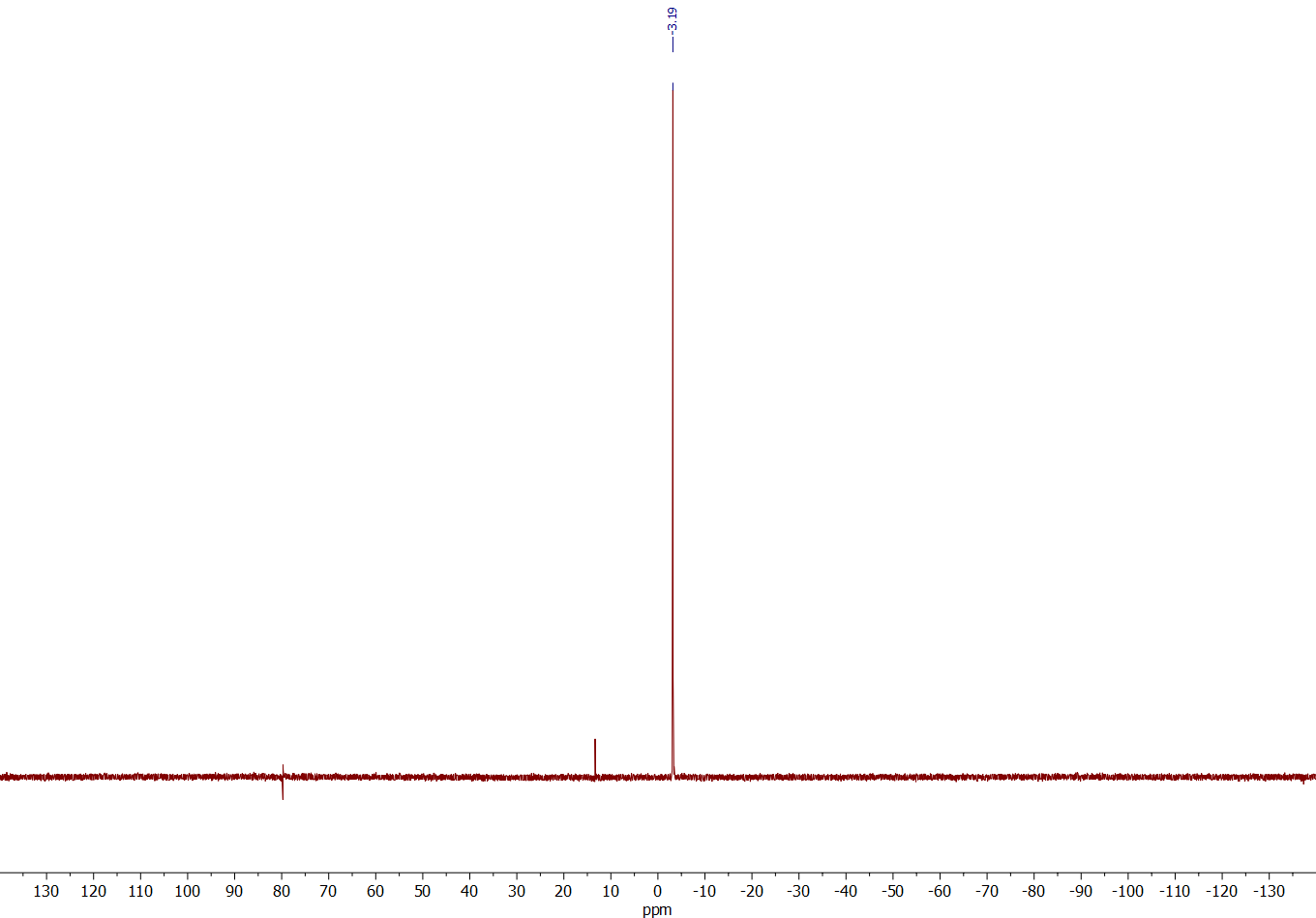


Figure S14. ^31^P{^1^H} NMR spectrum of **1^Rb^** in C_6_D_6_ at 300 K. Signals at 12 and 80 ppm belong to unknown impurities.


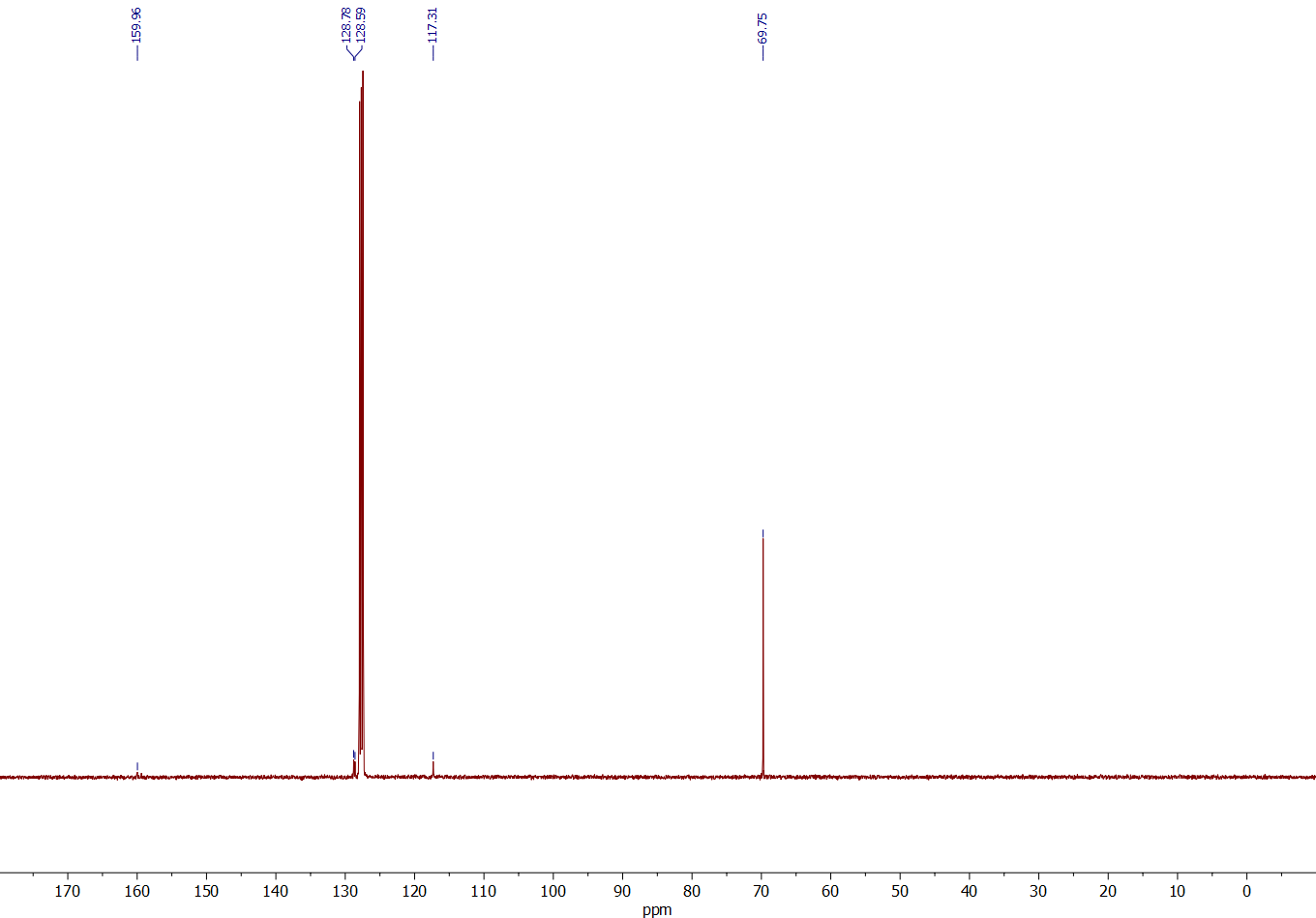


Figure S15. ^13^C NMR spectrum of **1^Rb^** in C_6_D_6_ at 300 K.

Figure S16. ^1^H NMR spectrum of **1^Cs^** in C_6_D_6_ at 300 K.


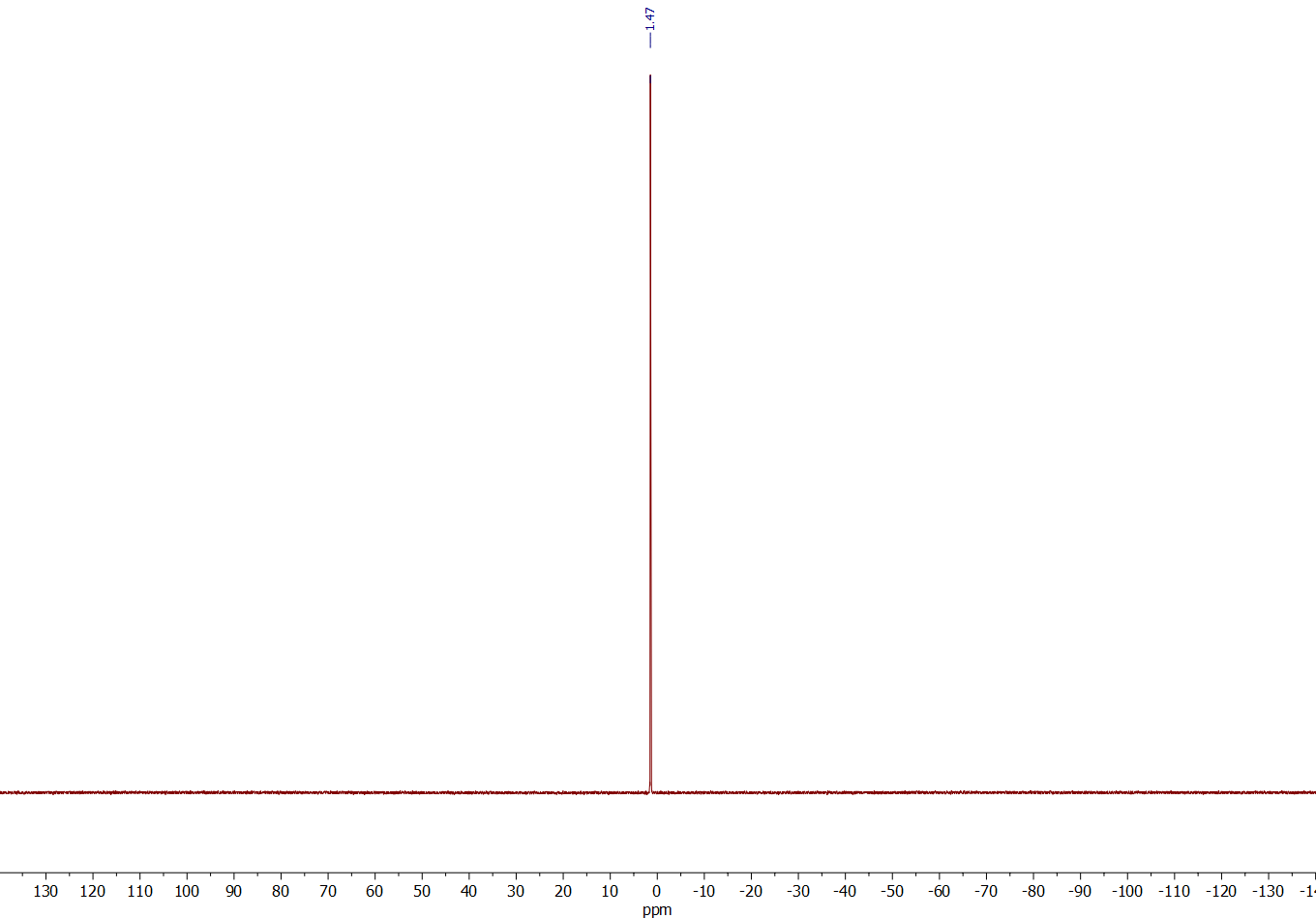


Figure S17. ^31^P{^1^H} NMR spectrum of **1^Cs^** in C_6_D_6_ at 300 K.


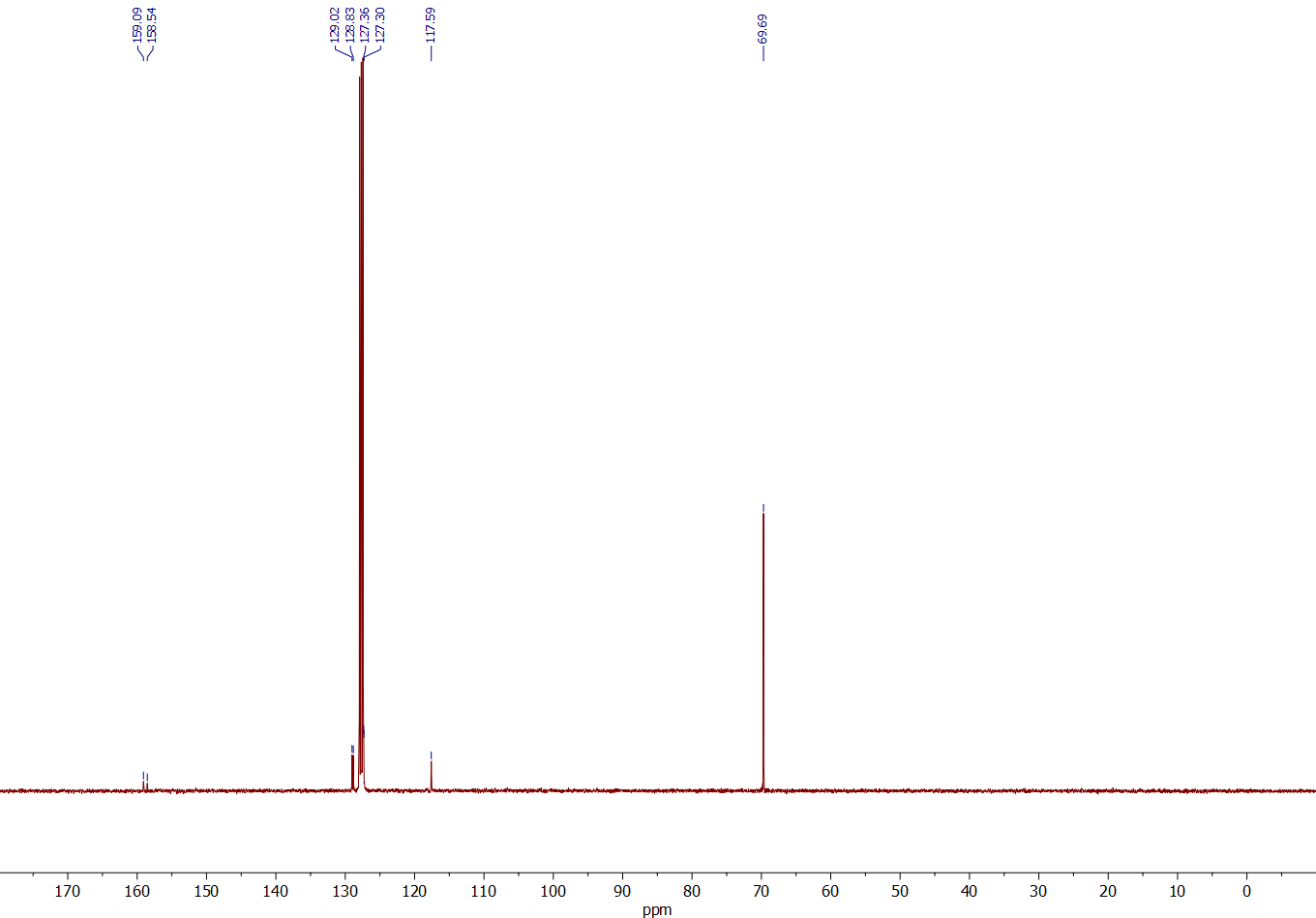


Figure S18. ^13^C NMR spectrum of **1^Cs^** in C_6_D_6_ at 300 K.


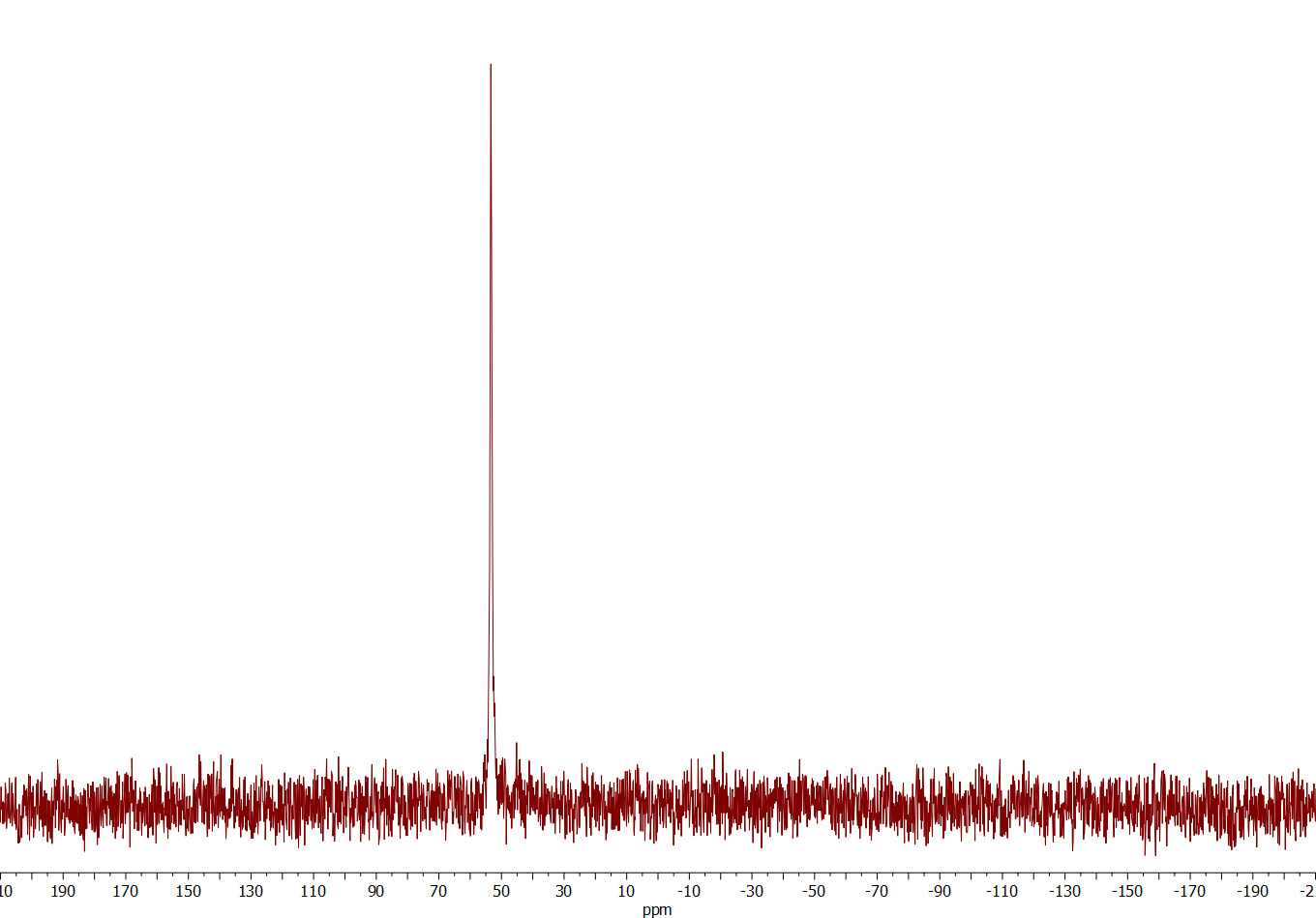


Figure S19. ^133^Cs NMR spectrum of **1^Cs^** in C_6_D_6_ at 300 K.

Figure S20. ^1^H NMR spectrum of **4^Cs^** in C_6_D_6_ at 300 K.


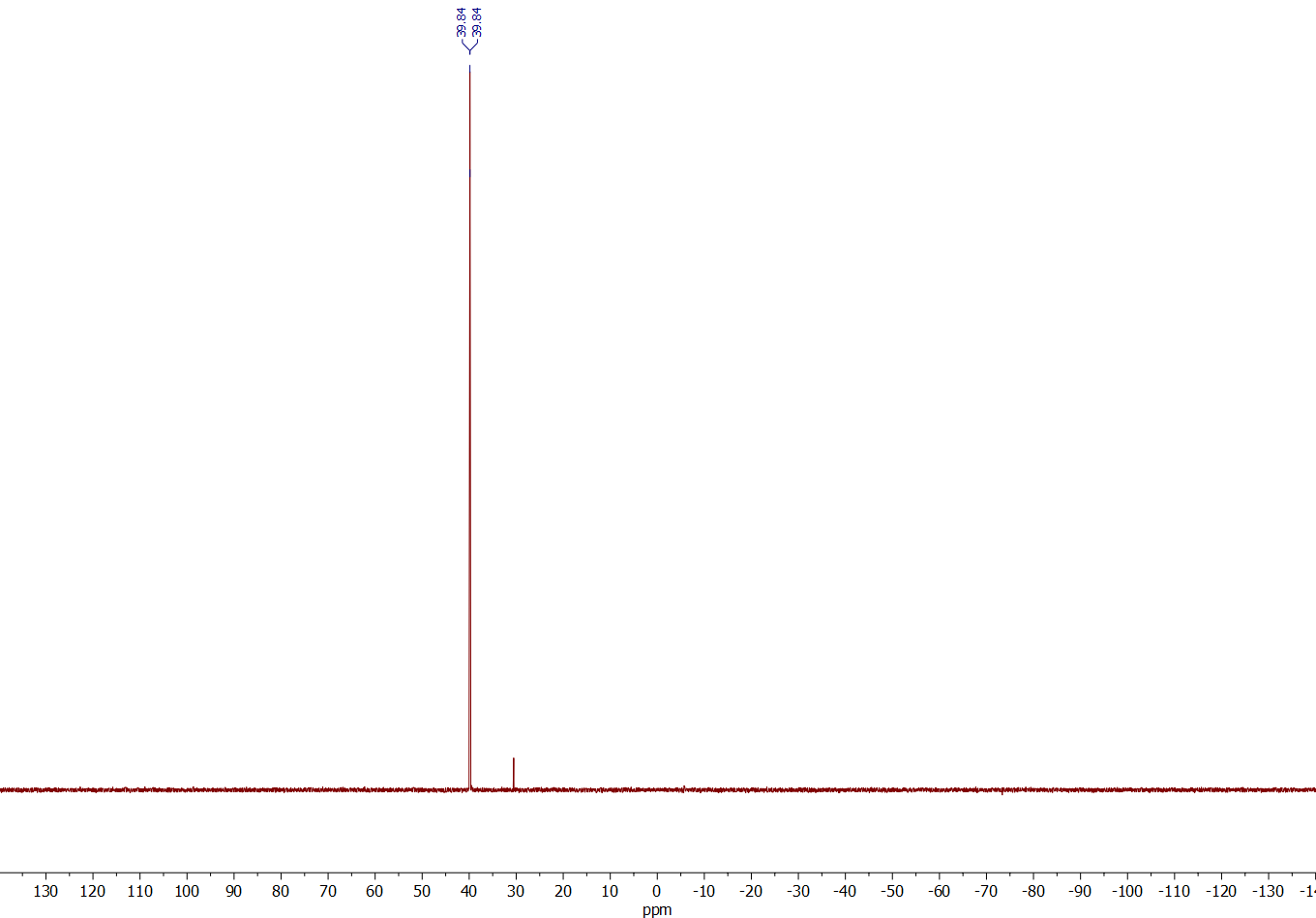


Figure S21. ^31^P{^1^H} NMR spectrum of **4^Cs^** in C_6_D_6_ at 300 K. Signals at 30 ppm belongs to an unknown impurity.


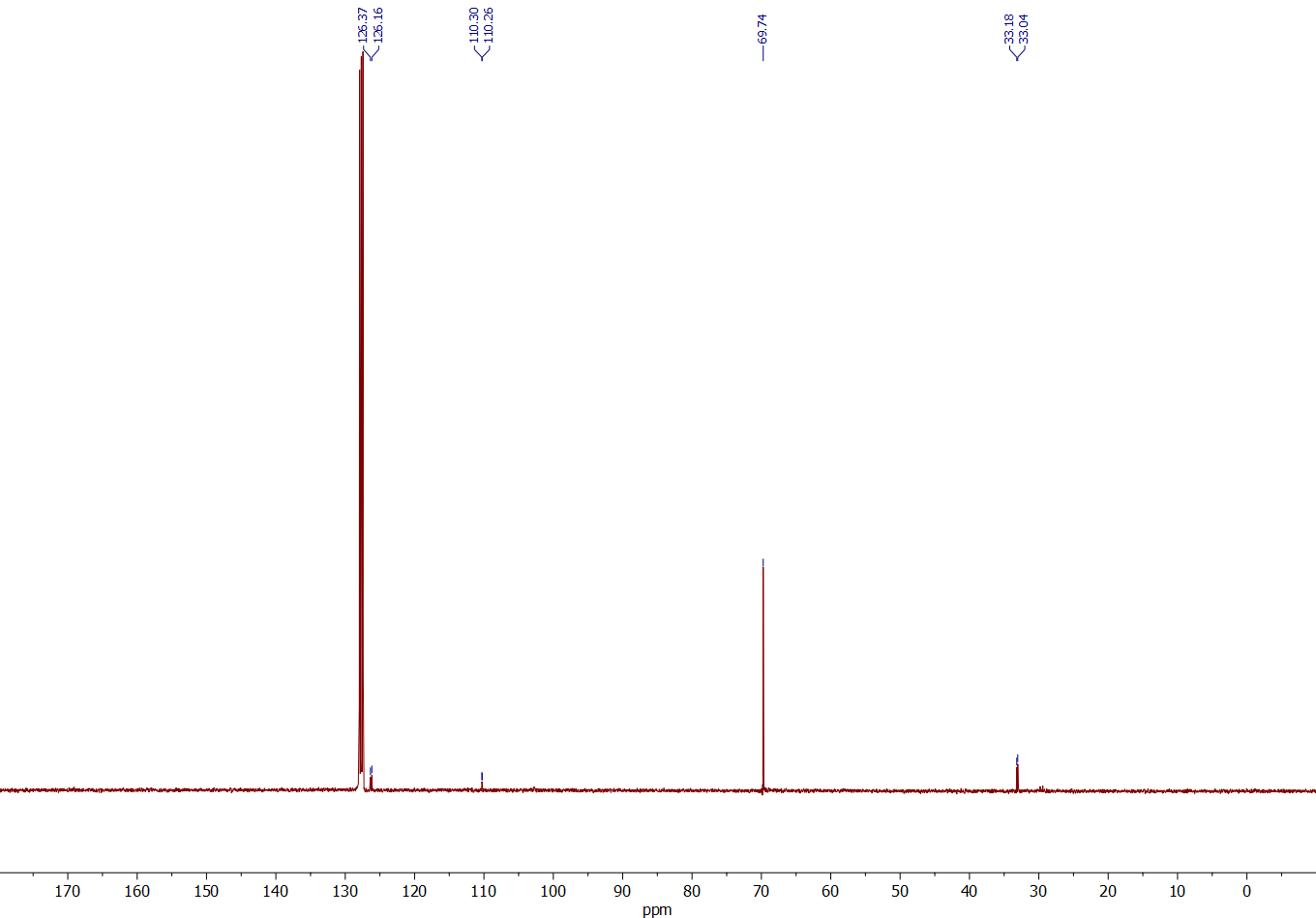


Figure S22. ^13^C NMR spectrum of **4^Cs^** in C_6_D_6_ at 300 K.


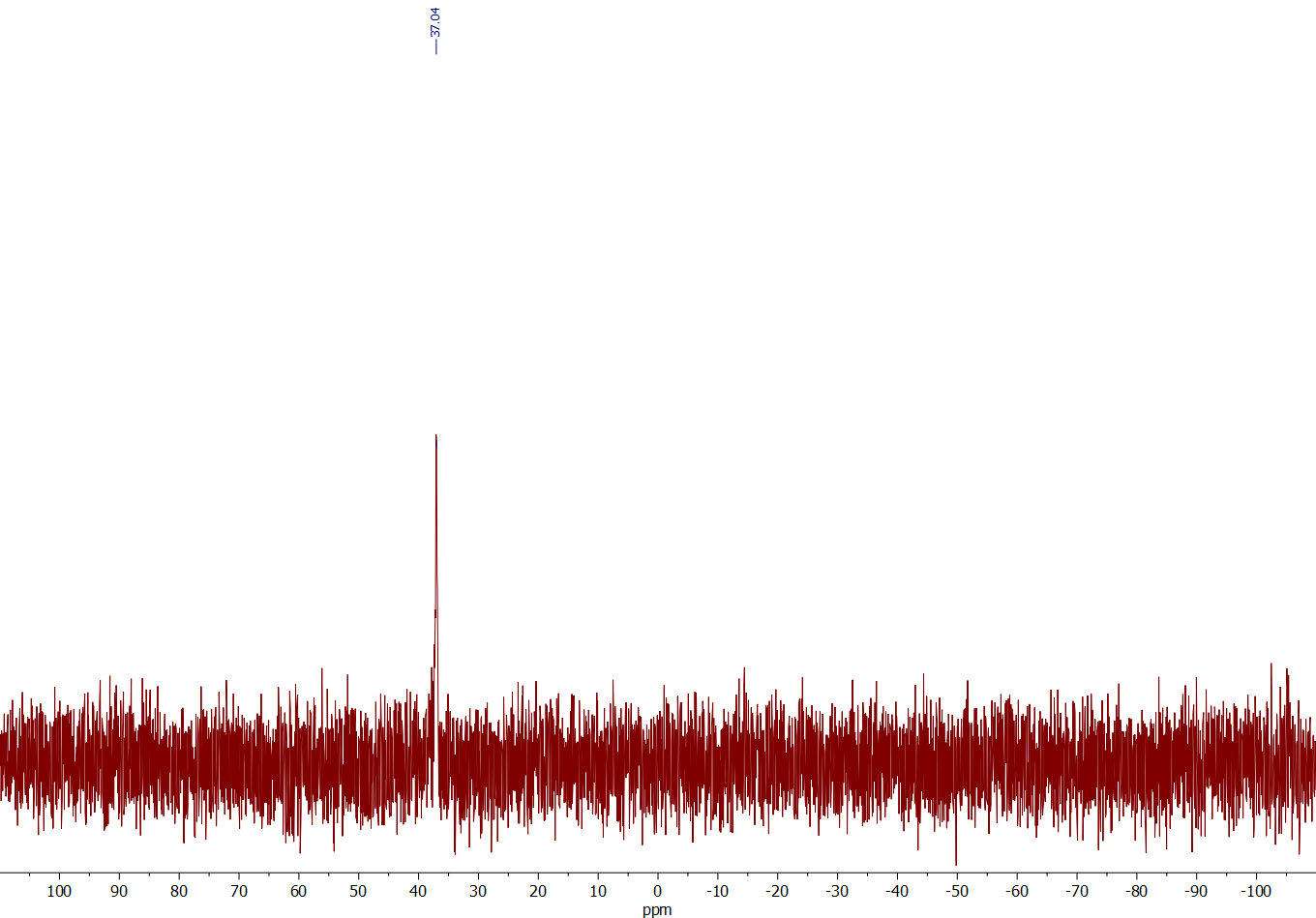


Figure S23. ^133^Cs NMR spectrum of **4^Cs^** in C_6_D_6_ at 300 K.

Figure S24. ^1^H NMR spectrum of **5^Cs^** in C_6_D_6_ at 300 K, with # marked signals belong to unidentified decomposition products.


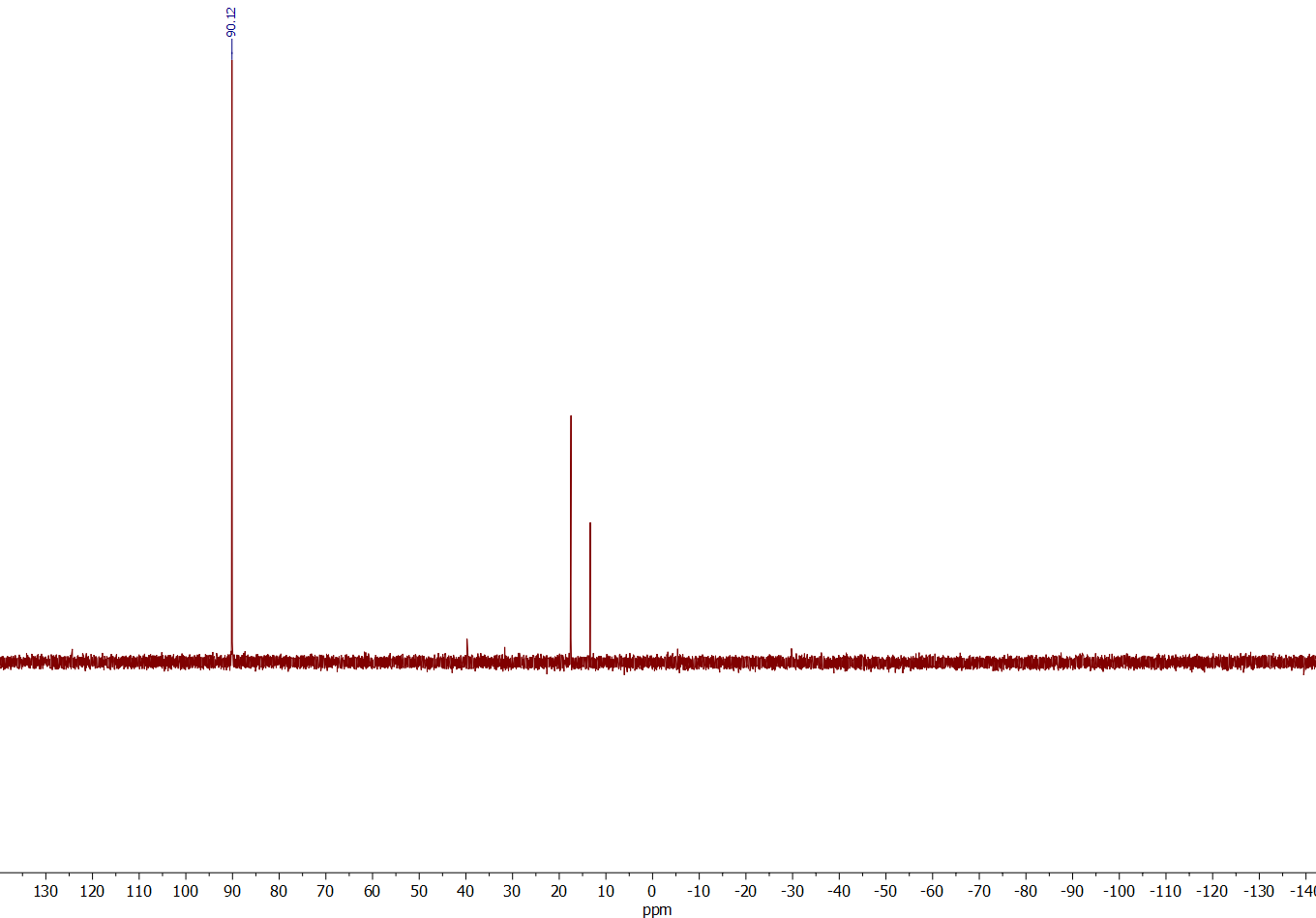


Figure S25. ^31^P{^1^H} NMR spectrum of **5^Cs^** in C_6_D_6_ at 300 K, signals at 17.5 and 13.4 ppm belong to unidentified decomposition products.


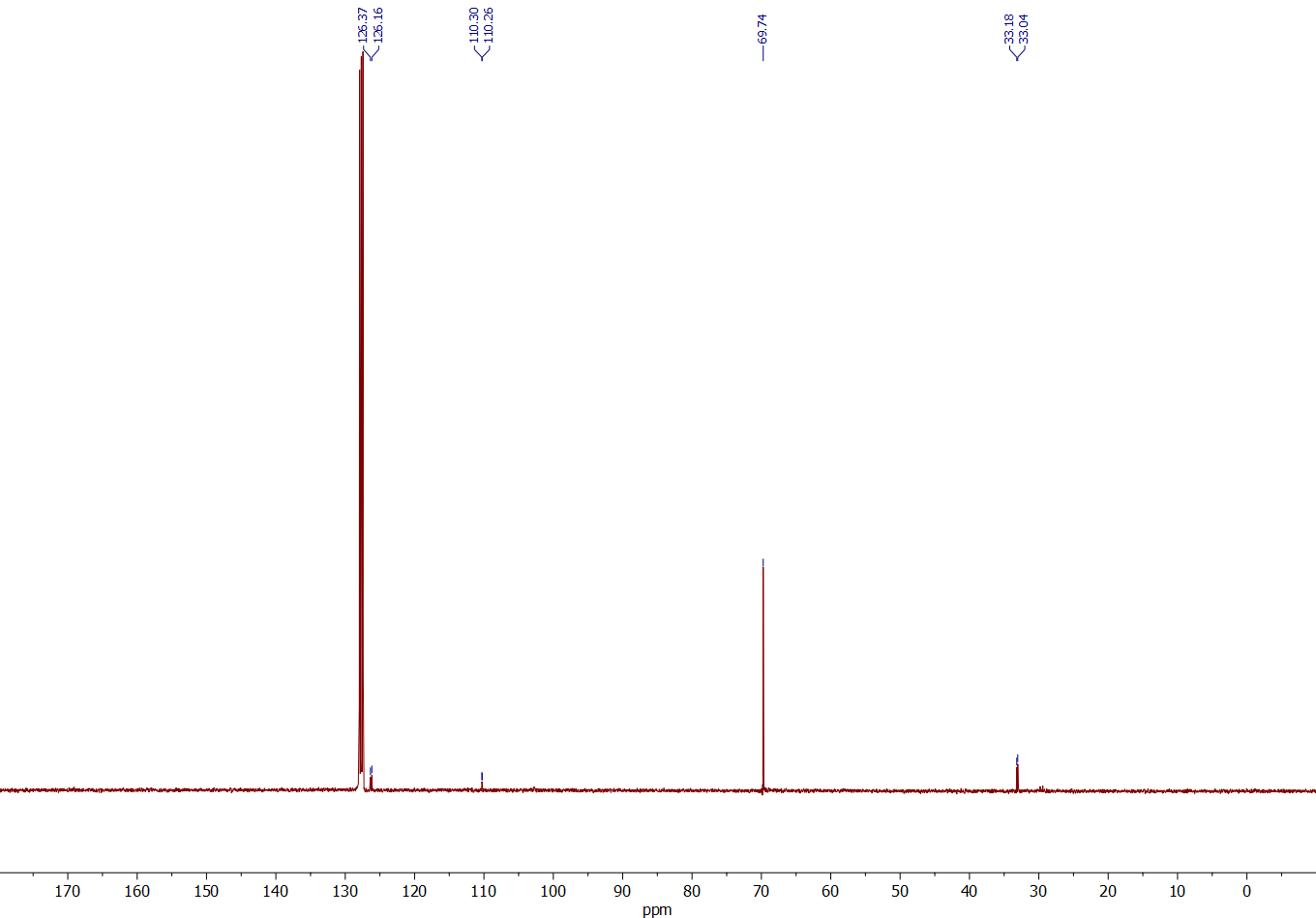


Figure S26. ^13^C NMR spectrum of **5^Cs^** in C_6_D_6_ at 300 K.


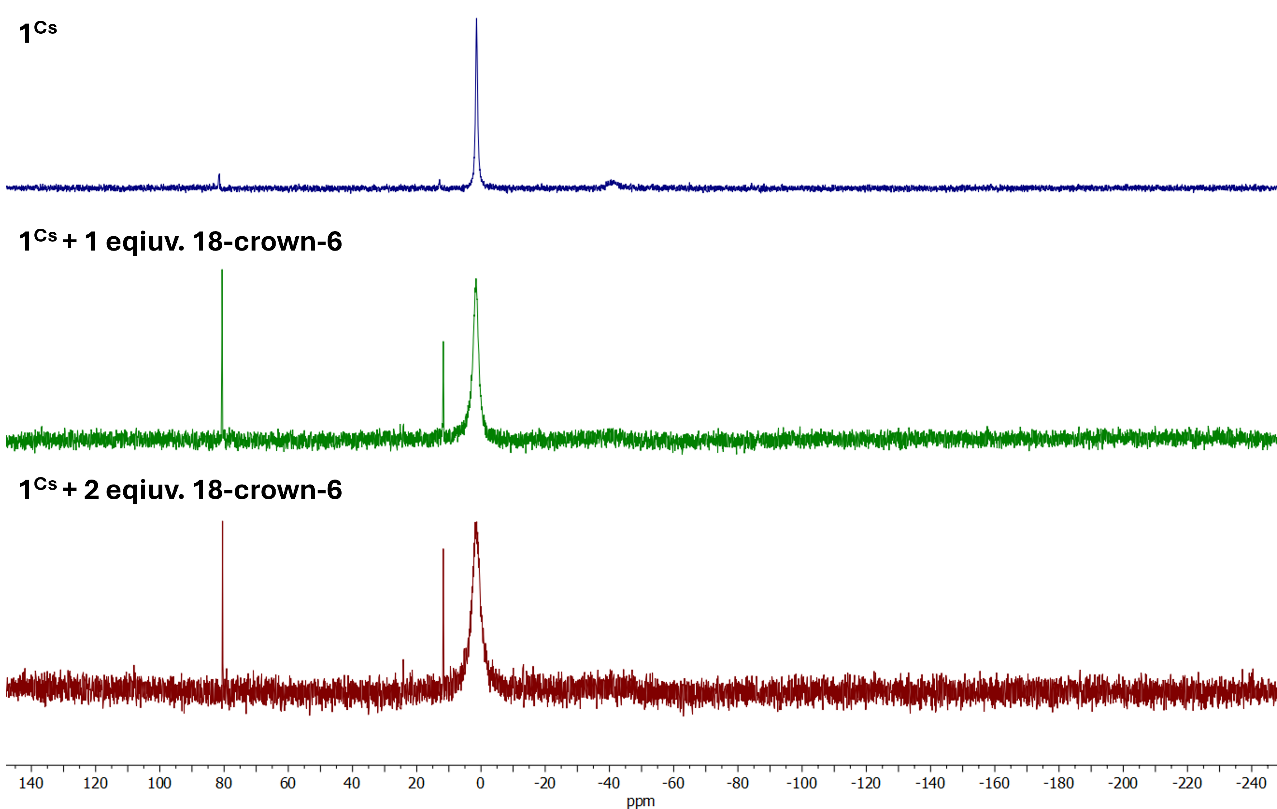


Figure S27. Stacked ^31^P{^1^H} NMR spectra of the addition of 18-crown-6 to **1^Cs^**.


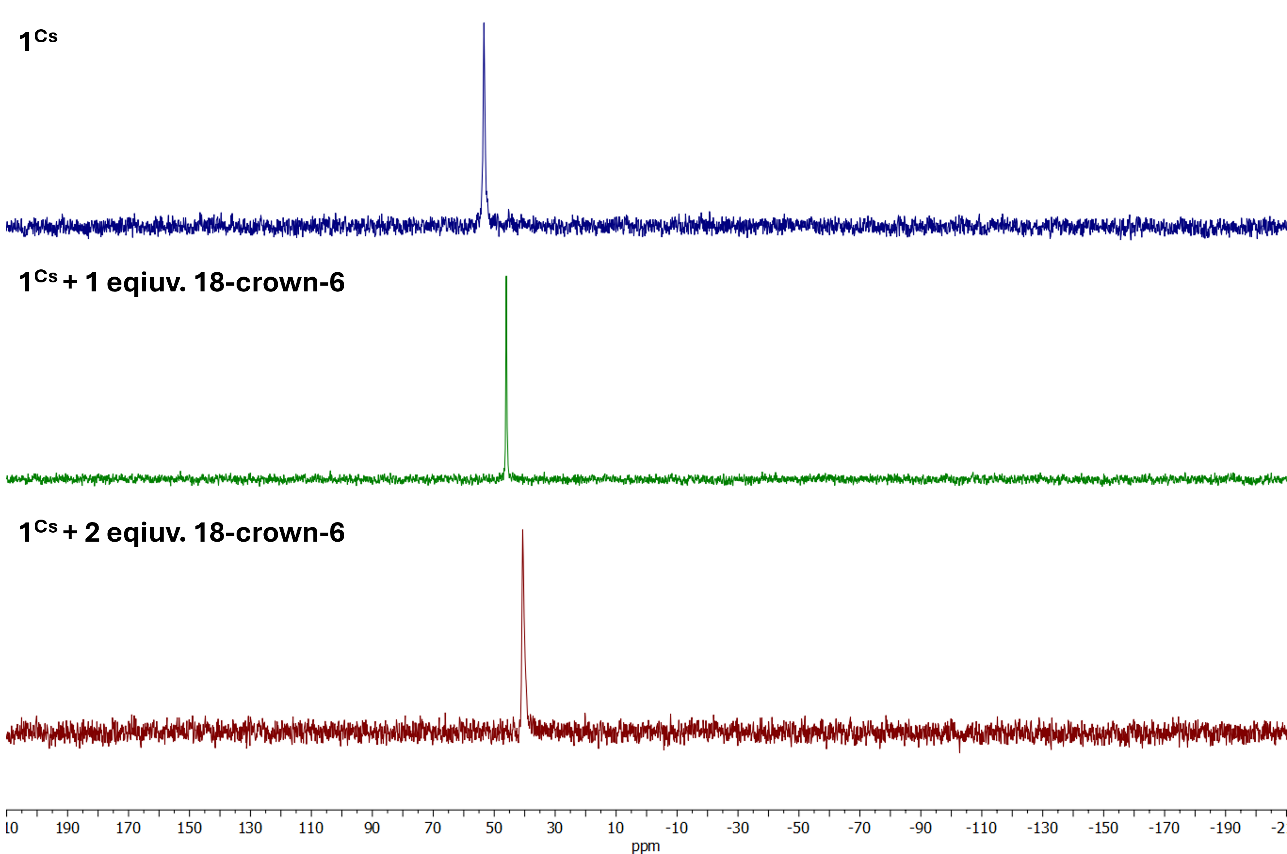


Figure S28. Stacked ^133^Cs NMR spectra of the addition of 18-crown-6 to **1^Cs^**.

# Section S2 – DOSY NMR studies

2D ^1^H Diffusion-Ordered Spectroscopy (DOSY) spectra were recorded on a Bruker AV400 spectrometer operating at 400.1 MHz for ^1^H and measured at 300 K. A 30 mM solution of the title compounds in 0.5 mL benzene-d_6_ and 0.01 mL toluene as an internal standard was. The diffusion coefficients Dc and Da were obtained by analysing the recorded data with the Dynamic Centre program package from Bruker and listed in Table S1. An estimate of the molecular weight (MW) of the species in solution was obtained *via* comparison of the diffusion coefficients of the compounds and internal standard toluene to external calibration curves (ECCs) with normalised diffusion coefficients.^[[1]](#footnote-1)^ The ECCs for molecules which diffuse like compact spheres (CS), dissipated spheres and ellipsoids (DSE), extended discs (ED) and a merge of all three were utilised. For species with multiple ^1^H signals, the average diffusion coefficient was taken. The accuracy of this estimation is in the range of MW_dif_ ± 9 %.^1^ In addition, the corresponding hydrodynamic radii r_H_ were calculated according to the Stokes-Einstein equation (Eq. 1) with a specific viscosity for C_6_D_6_ of *η* = 0.4047·10^-3^ kg m^2^ s^-2^.

$r_{H}=\frac{k_{B}T}{6\pi\eta D}$ Eq. 1

Table S1. Measured DOSY-NMR data.

|  | Da1 / ^-10^m^2^/s | Da2 / ^-10^m^2^/s | ⌀ Da / ^-10^m^2^/s | Dc / ^-10^m^2^/s | DTol / ^-10^m^2^/s | Dc/Da |
| --- | --- | --- | --- | --- | --- | --- |
| **1Li** | 6.07E-10 | 6.30E-10 | 6.19E-10 | 6.52E-10 | 1.79E-09 | 1.05 |
| **1Na** | 5.64E-10 | 6.09E-10 | 5.87E-10 | 5.88E-10 | 1.79E-09 | 1.00 |
| **1K** | 5.66E-10 | 6.07E-10 | 5.87E-10 | 5.71E-10 | 1.83E-09 | 0.97 |
| **1Rb** | 5.51E-10 | 5.44E-10 | 5.48E-10 | 5.55E-10 | 1.81E-09 | 1.01 |
| **1Cs** | 4.76E-10 | 5.09E-10 | 4.93E-10 | 4.87E-10 | 1.80E-09 | 0.99 |
| **3Cs** | 5.74E-10 | 5.53E-10 | 5.68E-10 | 5.78E-10 | 1.77E-09 | 1.02 |
| **4Cs** | - | 6.73E-10 | - | 7.63E-10 | 1.81E-09 | 1.13 |


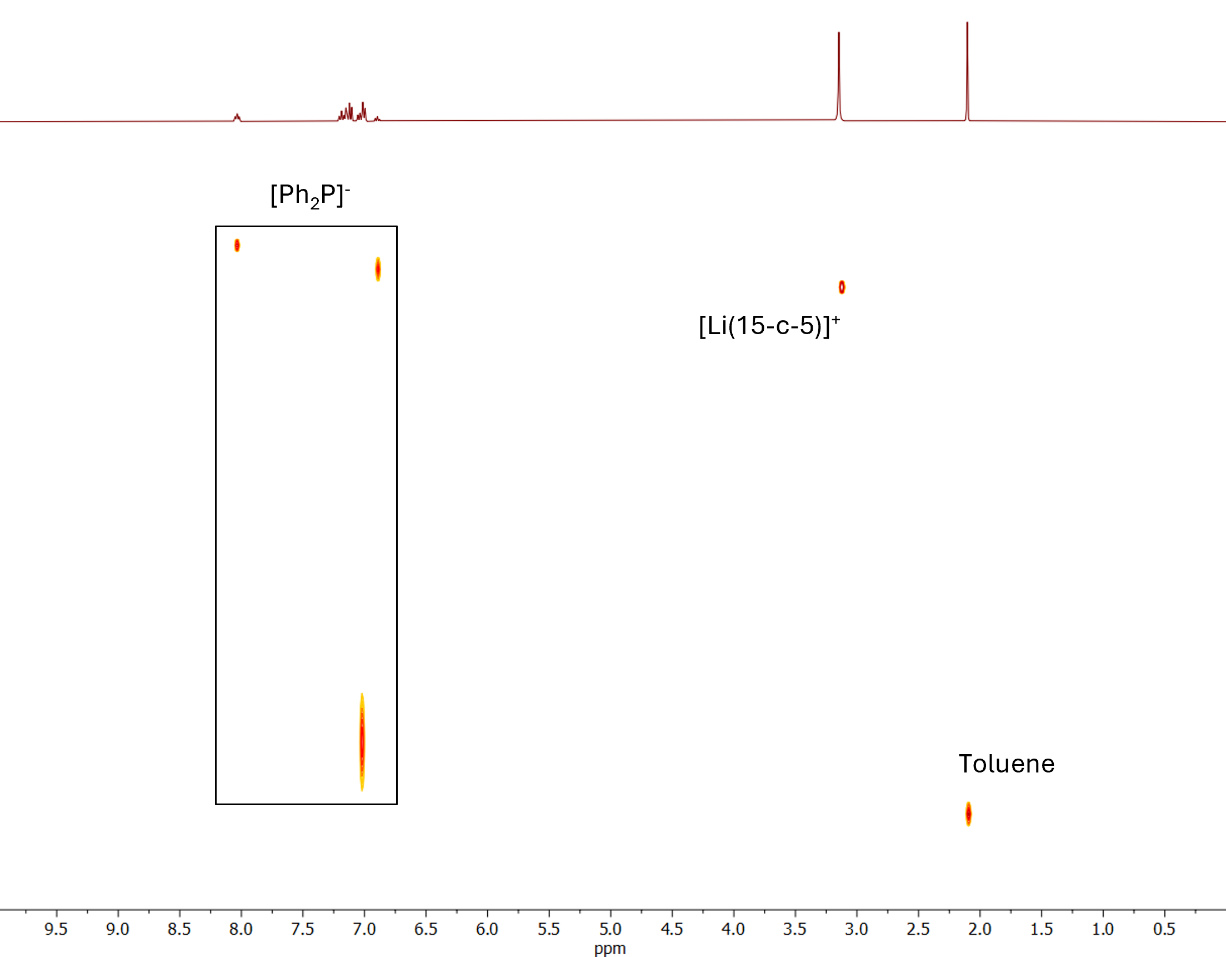


Figure S29. DOSY NMR spectrum of **1^Li^**.


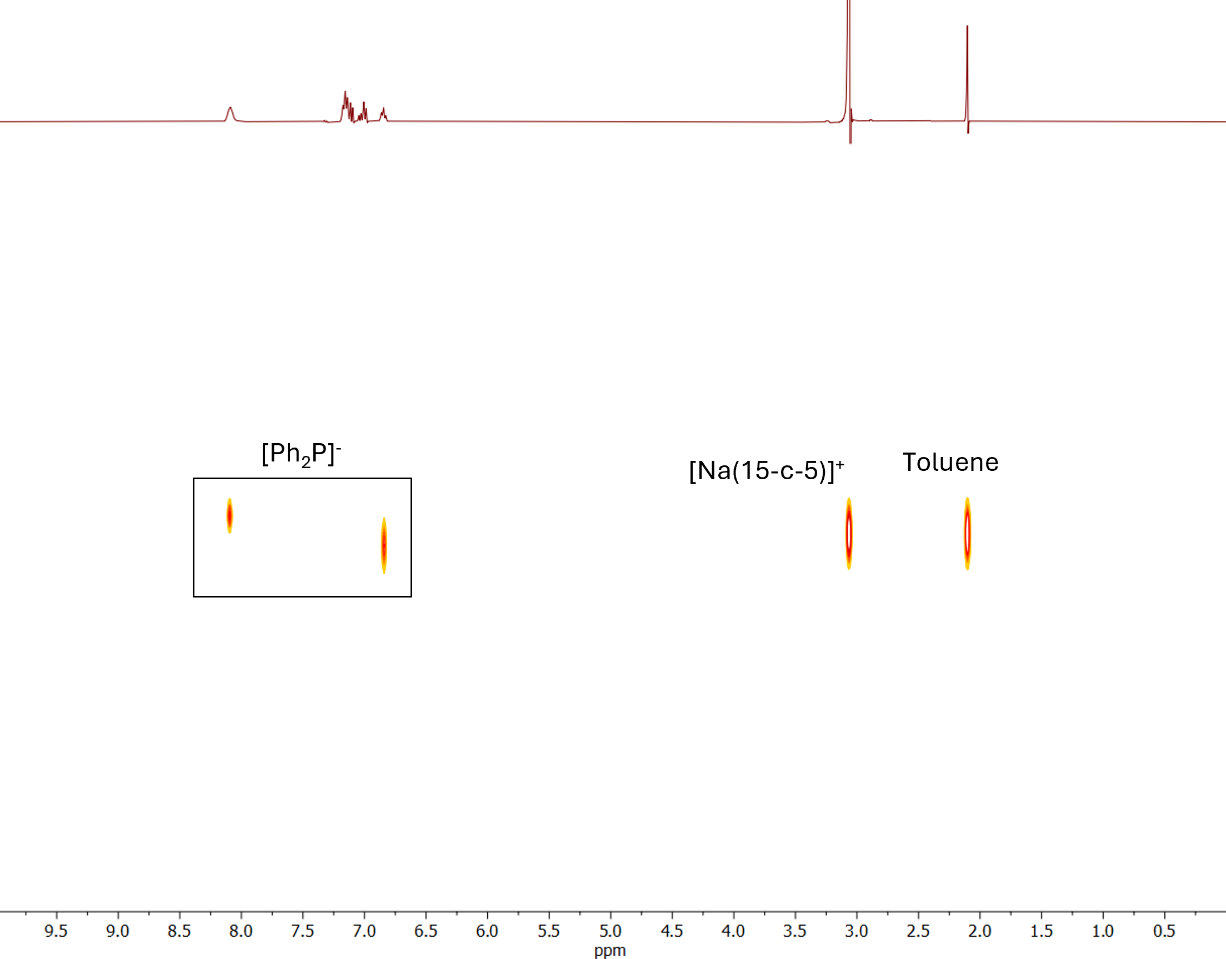


Figure S30. DOSY NMR spectrum of **1^Na^**.


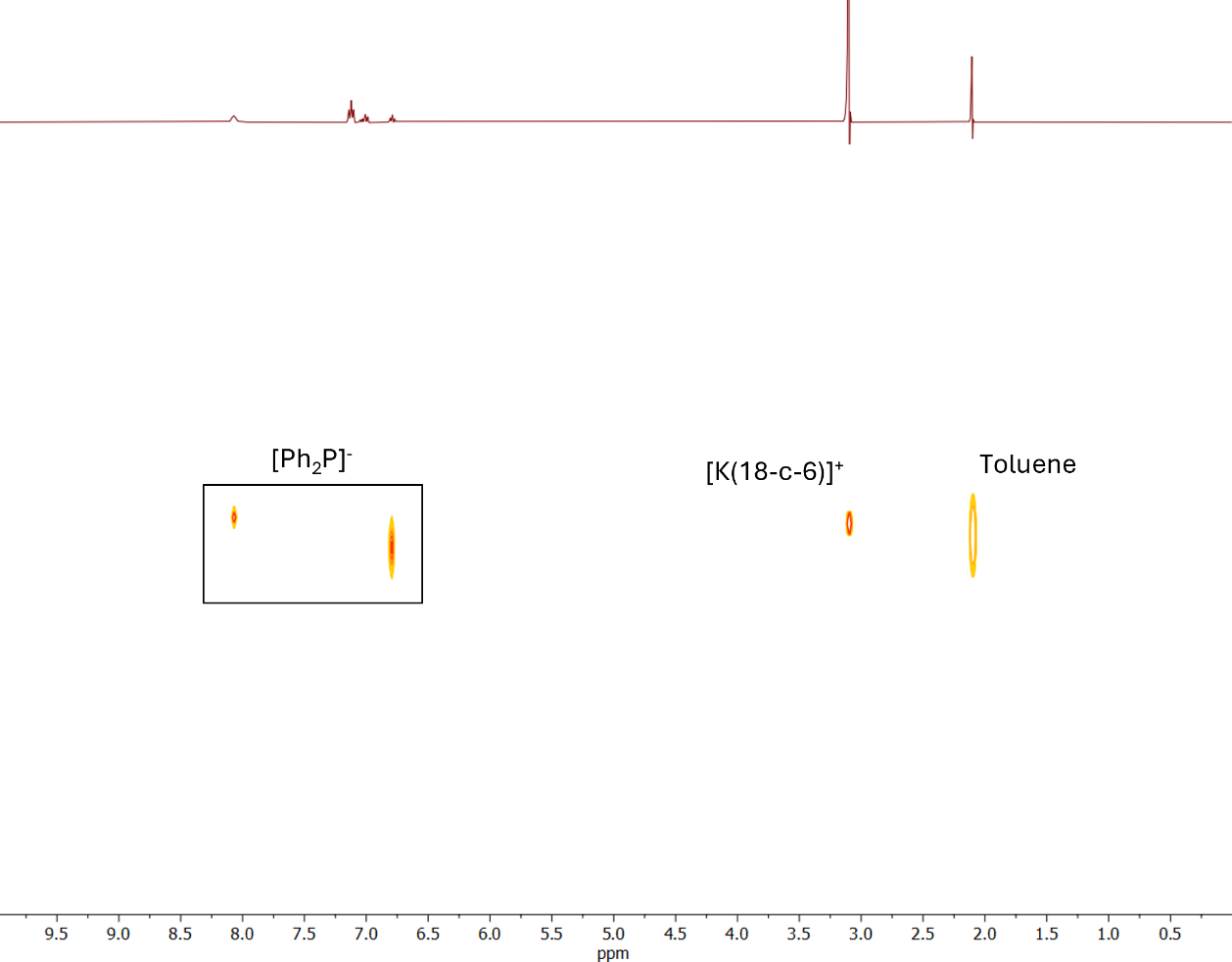


Figure S31. DOSY NMR spectrum of **1^K^**.


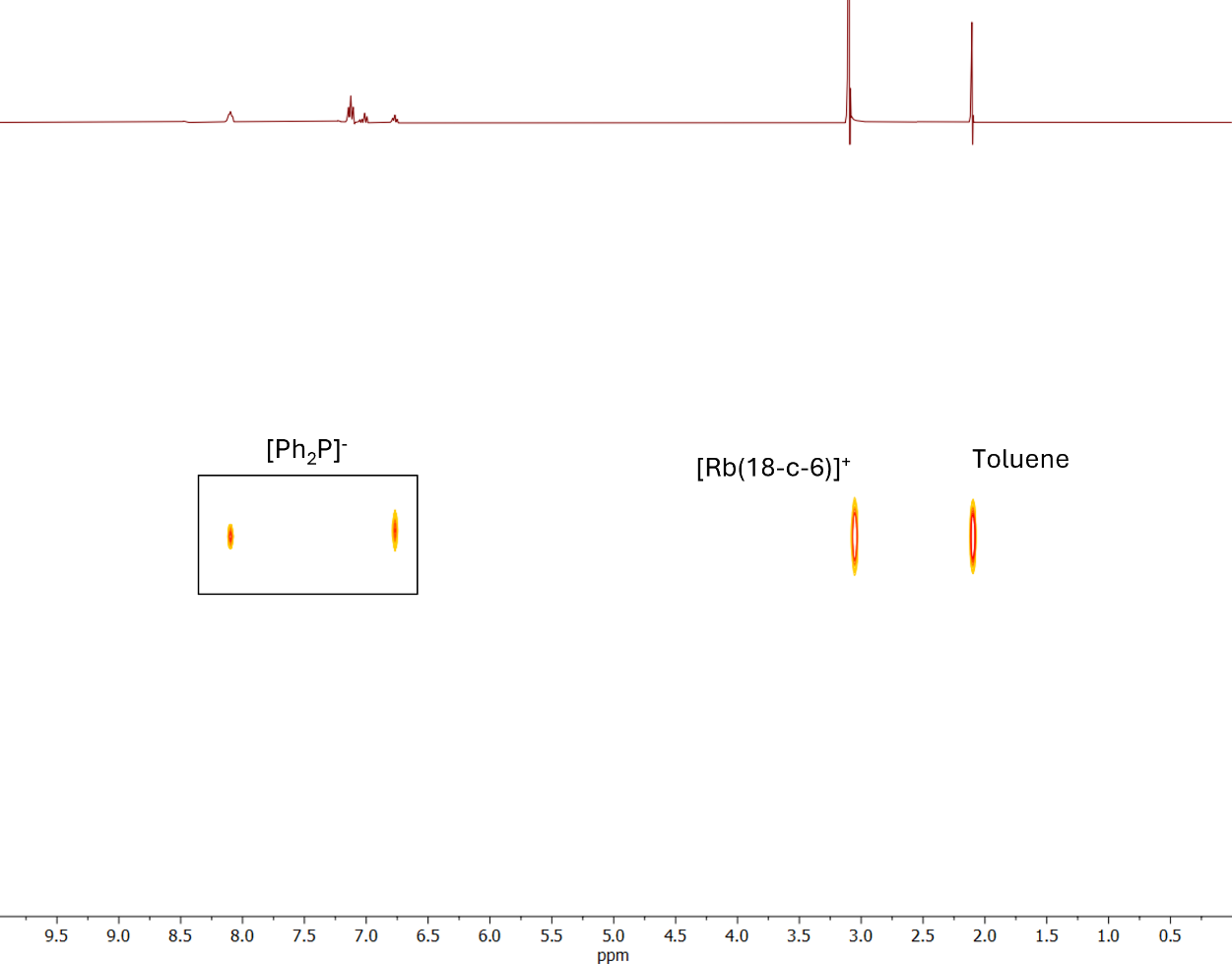


Figure S32. DOSY NMR spectrum of **1^Rb^**.


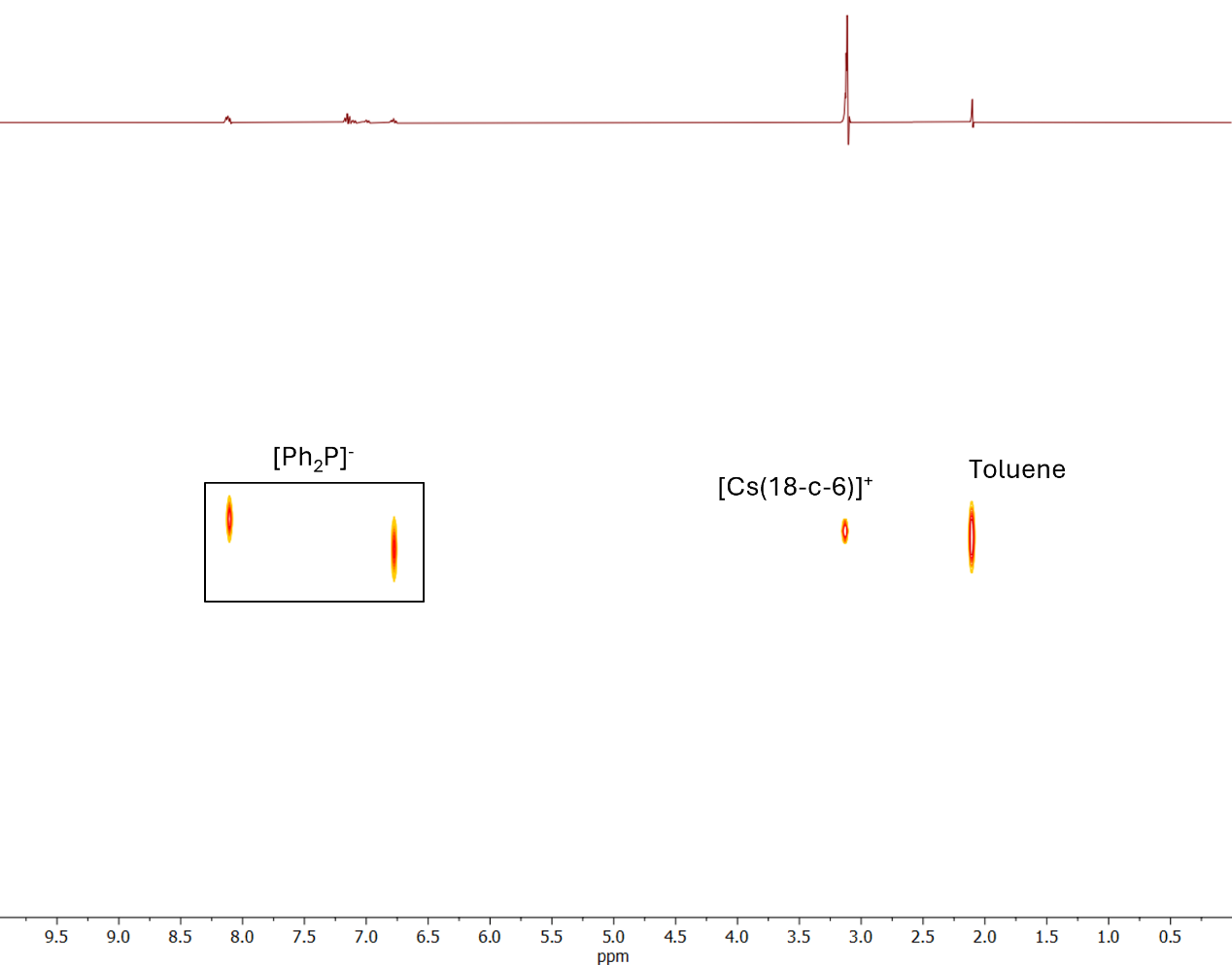


Figure D33. DOSY NMR spectrum of **1^Cs^**.


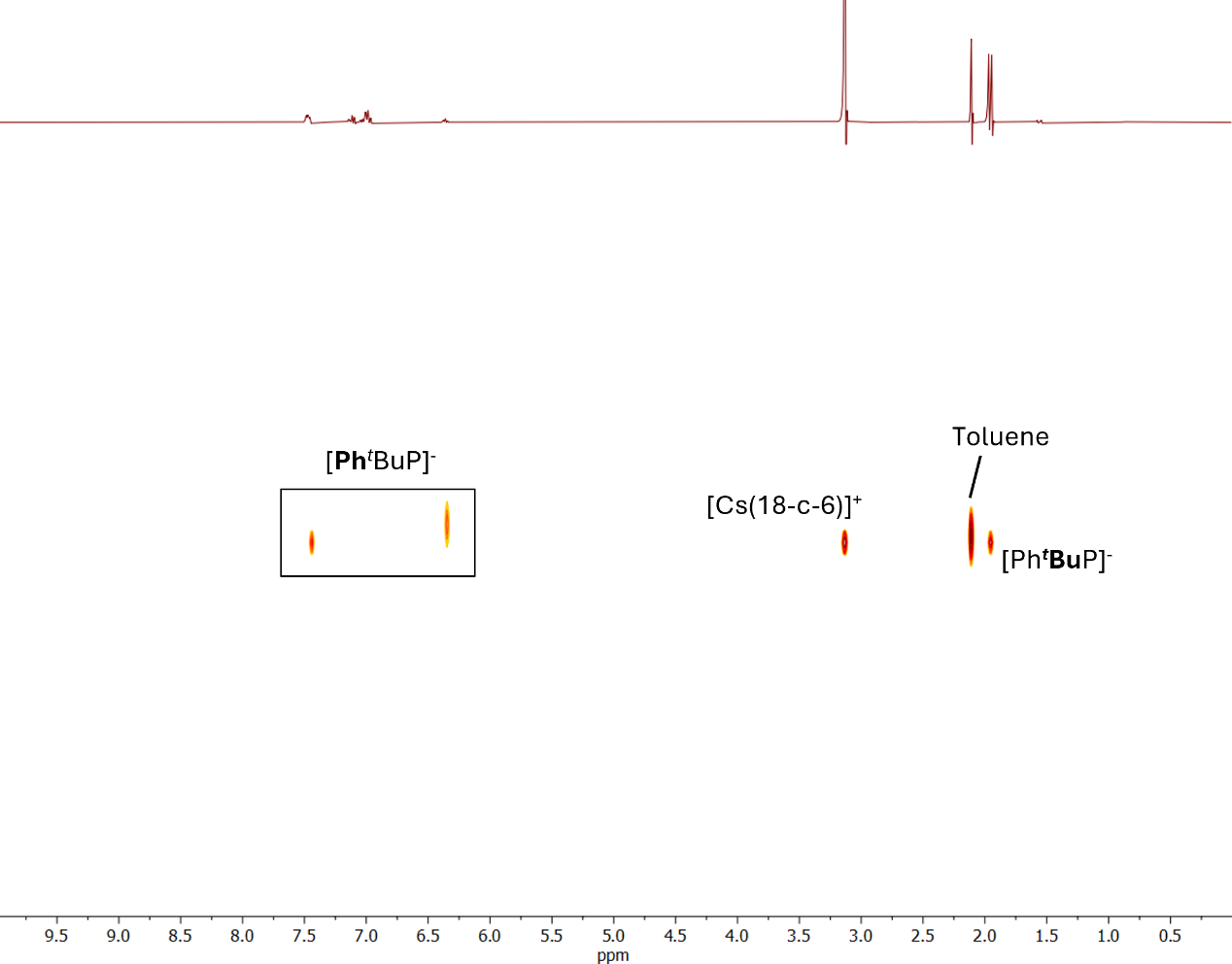


Figure S34. DOSY NMR spectrum of **4^Cs^**.


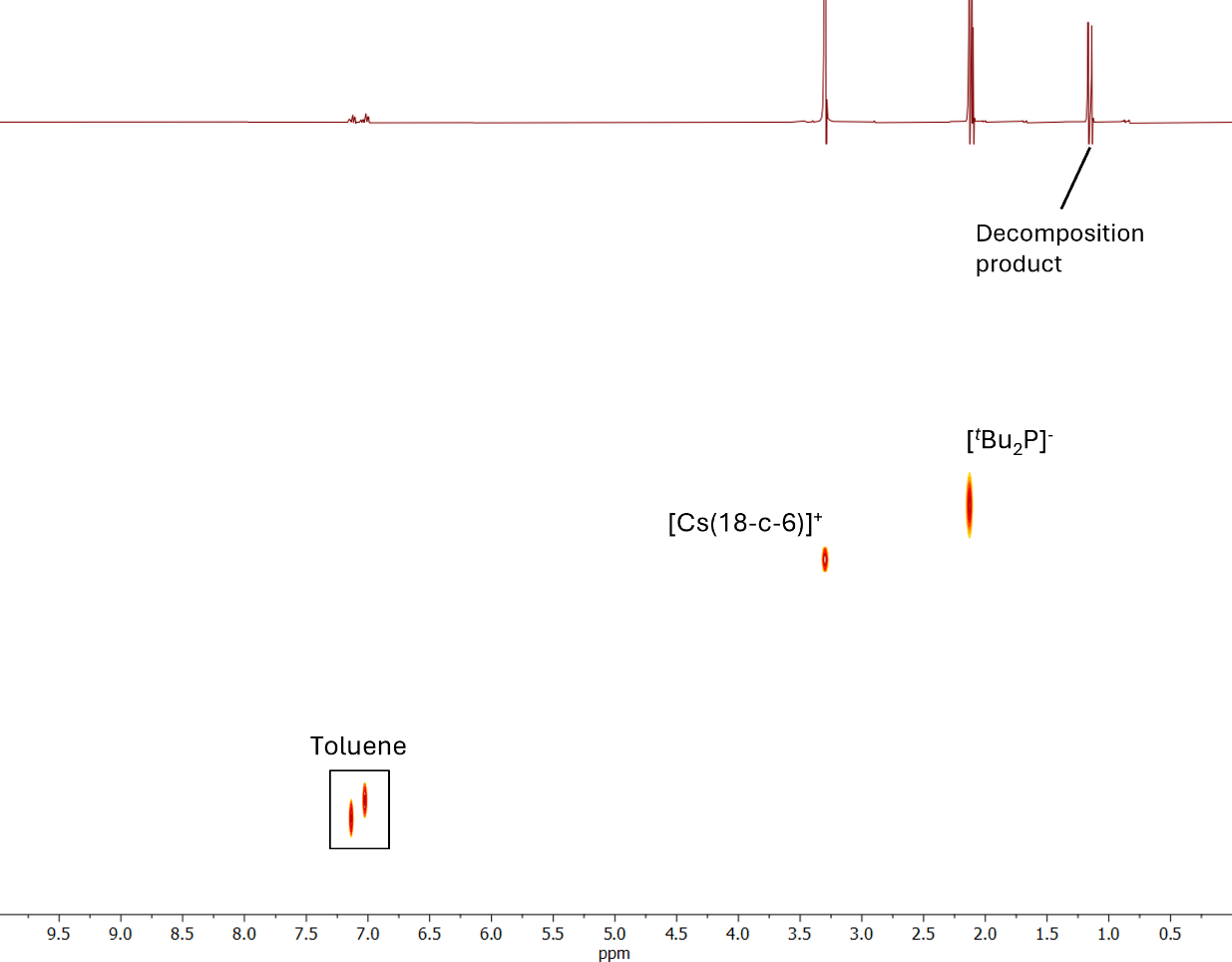


Figure S35. DOSY NMR spectrum of **5^Cs^**.

# Section S3 – Crystallographic details

| Table S2. Selected crystallographic and refinement parameters.   \| Compound \| **1^Li^** \| **2^Li^** \| **1^Rb^** \| **1^Rb‘^** \| \| --- \| --- \| --- \| --- \| --- \| \| CCDC# \| 2467228 \| 2467229 \| 2467230 \| 2467231 \| \| Empirical formula \| C_22_H_30_LiO_5_P \| C_20_H_26_LiO_4_P \| C_24_H_34_O_6_PRb \| C_27.5_H_38_O_6_PRb \| \| Formula weight \| 412.37 \| 368.32 \| 534.95 \| 581.02 \| \| Temperature/K \| 123(2) \| 102(2) \| 100(2) \| 100(2) \| \| Crystal system \| monoclinic \| monoclinic \| tetragonal \| monoclinic \| \| Space group \| P2_1_/n \| P2_1_/c \| I4 \| P2_1_/c \| \| a/Å \| 9.1729(1) \| 14.0154(2) \| 25.1596(1) \| 20.12196(7) \| \| b/Å \| 15.7730(2) \| 9.1654(1) \| 25.1596(1) \| 14.31027(4) \| \| c/Å \| 14.9967(2) \| 15.7999(3) \| 8.5149(1) \| 19.93499(6) \| \| α/° \| 90 \| 90 \| 90 \| 90 \| \| β/° \| 92.724(1) \| 105.013(2) \| 90 \| 92.2406(3) \| \| γ/° \| 90 \| 90 \| 90 \| 90 \| \| Volume/Å^3^ \| 2167.33(5) \| 1960.33(5) \| 5389.98(8) \| 5735.91(3) \| \| Z \| 4 \| 4 \| 8 \| 8 \| \| ρ_calc_g/cm^3^ \| 1.264 \| 1.248 \| 1.318 \| 1.346 \| \| μ/mm^‑1^ \| 1.366 \| 1.411 \| 3.367 \| 3.208 \| \| F(000) \| 880 \| 784 \| 2224 \| 2424 \| \| 2Θ max/° \| 146.282 \| 143.006 \| 146.234 \| 145.698 \| \| Reflections collected \| 14884 \| 10558 \| 57059 \| 118694 \| \| Independent reflections \| 4324 \| 3769 \| 4982 \| 11440 \| \| Rint \| 0.0212 \| 0.0340 \| 0.0567 \| 0.0518 \| \| restraints/parameters \| 0/262 \| 0/235 \| 1/290 \| 0/642 \| \| Goodness-of-fit on F^2^ \| 1.026 \| 1.056 \| 1.006 \| 1.049 \| \| Final R indexes [I>=2σ (I)] \| R_1_ = 0.0326, wR_2_ = 0.0807 \| R_1_ = 0.0383, wR_2_ = 0.1047 \| R_1_ = 0.0251, wR_2_ = 0.0585 \| R_1_ = 0.0354, wR_2_ = 0.0959 \| \| Final R indexes [all data] \| R_1_ = 0.0354, wR_2_ = 0.0825 \| R_1_ = 0.0413, wR_2_ = 0.1073 \| R_1_ = 0.0257, wR_2_ = 0.0594 \| R_1_ = 0.0363, wR_2_ = 0.0964 \| \| Largest diff. peak/hole / e Å^-3^ \| 0.456/-0.228 \| 0.417/-0.417 \| 0.254/-0.292 \| 0.968/-0.536 \| |
| --- | --- | --- | --- | --- | --- | --- | --- | --- | --- | --- | --- | --- | --- | --- | --- | --- | --- | --- | --- | --- | --- | --- | --- | --- | --- | --- | --- | --- | --- | --- | --- | --- | --- | --- | --- | --- | --- | --- | --- | --- | --- | --- | --- | --- | --- | --- | --- | --- | --- | --- | --- | --- | --- | --- | --- | --- | --- | --- | --- | --- | --- | --- | --- | --- | --- | --- | --- | --- | --- | --- | --- | --- | --- | --- | --- | --- | --- | --- | --- | --- | --- | --- | --- | --- | --- | --- | --- | --- | --- | --- | --- | --- | --- | --- | --- | --- | --- | --- | --- | --- | --- | --- | --- | --- | --- | --- | --- | --- | --- | --- | --- | --- | --- | --- | --- | --- | --- | --- | --- | --- | --- | --- | --- | --- | --- | --- | --- | --- | --- | --- | --- | --- | --- | --- | --- |

| Compound | **1^Cs^** | **1^Cs^** (Sandwich) | **1^Cs^** (Club Sandwich) | **4^Cs^** | **5^Cs^** |
| --- | --- | --- | --- | --- | --- |
| CCDC# | 2467232 | 2467233 | 2467234 | 2467235 | 2467236 |
| Empirical formula | C_24_H_34_CsO_6_P | C_36_H_56_CsO_12_P | C_60_H_92_Cs_2_O_18_P_2_ | C_22_H_38_CsO_6_P | C_20_H_42_CsO_6_P |
| Formula weight | 582.39 | 844.68 | 1429.09 | 562.40 | 542.41 |
| Temperature/K | 100(2) | 100(2) | 100(2) | 100(2) | 150(2) |
| Crystal system | monoclinic | triclinic | triclinic | orthorhombic | monoclinic |
| Space group | P2_1_ | P-1 | P-1 | P2_1_2_1_2_1_ | P2_1_/n |
| a/Å | 23.4932(2) | 8.9538(2) | 9.9868(1) | 10.4064(1) | 12.1075(2) |
| b/Å | 13.8394(1) | 13.8705(3) | 13.8362(2) | 12.3838(1) | 8.9621(2) |
| c/Å | 25.5717(2) | 16.8922(1) | 14.1847(2) | 20.1464(2) | 24.5186(4) |
| α/° | 90 | 92.282(1) | 62.568(2) | 90 | 90 |
| β/° | 111.238(1) | 97.905(1) | 75.641(1) | 90 | 95.464(2) |
| γ/° | 90 | 102.227(2) | 69.336(2) | 90 | 90 |
| Volume/Å^3^ | 7749.50(12) | 2025.68(7) | 1619.09(5) | 2596.28(4) | 2648.39(9) |
| Z | 12 | 2 | 1 | 4 | 4 |
| ρ_calc_g/cm^3^ | 1.498 | 1.385 | 1.466 | 1.439 | 1.360 |
| μ/mm^‑1^ | 12.060 | 7.978 | 9.801 | 11.972 | 11.71 |
| F(000) | 3552 | 876 | 736 | 1152 | 1120 |
| 2Θ max/° | 146.988 | 139.996 | 143.934 | 146.100 | 143.026 |
| Reflections collected | 116915* | 43449 | 27784 | 9353 | 15484 |
| Independent reflections | 105131* | 7661 | 6190 | 4507 | 5110 |
| Rint | 0.0771* | 0.0983 | 0.0478 | 0.0277 | 0.0402 |
| restraints/parameters | 37/1863 | 99/479 | 0/378 | 0/282 | 3/290 |
| Goodness-of-fit on *F*^2^ | 1.003 | 1.098 | 1.096 | 1.052 | 1.161 |
| Final R indexes [I>=2σ (I)] | R_1_ = 0.0451,  wR_2_ = 0.1155 | R_1_ = 0.0568,  wR_2_ = 0.1555 | R_1_ = 0.0244,  wR_2_ = 0.0612 | R_1_ = 0.0240, wR_2_ = 0.0610 | R_1_ = 0.0523, wR_2_ = 0.1565 |
| Final R indexes [all data] | R_1_ = 0.0497, wR_2_ = 0.1179 | R_1_ = 0.0612, wR_2_ = 0.1569 | R_1_ = 0.0258, wR_2_ = 0.0618 | R_1_ = 0.0242, wR_2_ = 0.0611 | R_1_ = 0.0567, wR_2_ = 0.1597 |
| Largest diff. peak/hole / e Å^-3^ | 0.653/-1.657 | 1.614/-1.808 | 0.581/-0.732 | 0.703/-1.089 | 1.752/-1.225 |
|  |  |  |  |  |  |

* Treated as twinned and refined against an hklf 5 formatted reflection file.

# Section S4 – XYZ Files of the Optimized Structures

**1^Li^**

C 4.38352754669694 12.14522168299271 9.83904542458538

C 5.39758339511726 11.18518339866435 9.55293962882712

H 5.10729386315814 10.15088064233464 9.32392634174214

C 6.75385639791215 11.54210961097965 9.49924026361219

H 7.50667300333288 10.76552114816081 9.28324781227156

C 7.16063444495678 12.87615451680376 9.69789202930865

H 8.22573770114875 13.15202394124599 9.65817657217216

C 6.17309280102580 13.84992240859835 9.94786583400921

H 6.46468603713926 14.90177152375535 10.10341748269672

C 4.82037911636836 13.49071919189763 10.02541733551548

H 4.06563025026474 14.26416454169574 10.24520809772031

C 2.30827587440699 10.11688787306067 10.30474956248136

C 3.23440242962695 9.23369821240033 10.93159844952873

H 4.24095092177554 9.59529698709927 11.18631245000350

C 2.89324314860015 7.90808323487931 11.24279680429836

H 3.64636875729451 7.25793990305301 11.71834089819040

C 1.60790484285297 7.40437365006163 10.96709378285421

H 1.34657310316645 6.36245749402580 11.20787463644413

C 0.65678675783092 8.27412762996309 10.39436739053576

H -0.36564748360101 7.91474254240601 10.19049481431797

C 0.99934871507185 9.59301546624609 10.07397391311912

H 0.23926169660798 10.25069771821618 9.62198668362452

C 2.88457365646086 8.41957951645167 7.32807382226469

H 2.66760880724370 8.35808322062100 8.41040340044588

H 3.25462871038084 7.42582676867634 6.98279540621420

C 1.61390018863924 8.78527794242116 6.57174698574642

H 1.72456767763337 8.66716647133973 5.46614562341000

H 0.79434456907743 8.10237423475086 6.89607803929079

C 0.01325454728204 10.55778633370263 6.47214277519817

H -0.77072009578740 9.84594242339568 6.82064985566111

H -0.04054648158998 10.60789389109495 5.35743949591761

C -0.23426290159824 11.92039741961592 7.08404243552818

H -1.21021723482914 12.32663275255561 6.72576901790503

H -0.27662129827860 11.83992292666666 8.19508644605224

C 0.87581679646002 14.00864105411788 7.38088950192613

H 0.93575489180696 13.83561929801499 8.48075643588397

H -0.03482440083097 14.61234375559047 7.15618260824301

C 2.10777235672974 14.73475799451394 6.88701980938693

H 2.00422295312588 14.96223065966984 5.79885077021822

H 2.23012402988280 15.70160373788300 7.42959242609592

C 4.35243868573858 14.18269772323709 6.30027396413645

H 4.86191281936667 15.11205422466814 6.64920233993845

H 4.03806226124209 14.34008858338817 5.23976675224469

C 5.30871121947962 13.01682003888223 6.39749721114454

H 6.14538738479277 13.16985446476562 5.67696105806376

H 5.73977685775418 12.93492383119942 7.41876005837312

C 5.43044714245942 10.68823695548478 5.96159904622678

H 6.13128454770466 10.65052842244760 6.82539350697892

H 6.03124821106356 10.74308046256991 5.02321650208019

C 4.56108112729132 9.45337386045852 5.95308118567537

H 3.84586614165786 9.50197361208200 5.09771426523133

H 5.19524071738681 8.54538373191163 5.81187269310642

Li 2.76352494076765 11.67537577403137 7.27156727715285

O 3.21932952260819 13.87581654073950 7.09499148317797

O 4.57912637837413 11.82690570027219 6.08003900949593

O 3.88494982565037 9.42156472159318 7.19969229880162

O 1.30312594792476 10.13546306391573 6.88924323402605

O 0.84698946842757 12.75632155100041 6.69944773309315

P 2.57545670774651 11.86006301772859 9.84374931780402

**2^Li^**

P 3.688899000000 3.708274000000 10.644564000000

Li 3.265980000000 3.907890000000 8.188878000000

O 1.560835000000 4.688843000000 7.485576000000

O 2.449818000000 2.057372000000 7.333918000000

O 4.607310000000 3.345600000000 6.647683000000

O 4.071307000000 5.765836000000 7.651736000000

C 3.673259000000 6.376545000000 11.773623000000

H 4.699970000000 6.198177000000 12.127086000000

C 3.003036000000 5.364796000000 11.031202000000

C 5.478247000000 3.974414000000 10.380317000000

C 3.048469000000 7.592287000000 12.088670000000

H 3.604994000000 8.352381000000 12.661325000000

C 6.126974000000 5.208044000000 10.084697000000

H 5.534329000000 6.132431000000 10.049609000000

C 1.022665000000 6.841195000000 10.995965000000

C 3.546746000000 1.257569000000 6.867984000000

C 0.741352000000 3.659511000000 6.919934000000

C 1.597199000000 2.565332000000 6.303790000000

H 0.938926000000 1.762516000000 5.895459000000

H 2.205520000000 2.973042000000 5.464558000000

C 1.718636000000 7.841844000000 11.700161000000

H 1.230503000000 8.796344000000 11.950819000000

C 6.300636000000 2.807898000000 10.360070000000

H 5.843543000000 1.834627000000 10.608539000000

C 4.856728000000 2.011967000000 7.088211000000

C 5.689080000000 4.272527000000 6.750908000000

H 6.435922000000 4.115063000000 5.938874000000

H 6.204695000000 4.167092000000 7.729848000000

C 7.664401000000 2.864524000000 10.040944000000

H 8.257947000000 1.935566000000 10.033737000000

C 7.496124000000 5.265762000000 9.787019000000

H 7.956265000000 6.242512000000 9.562593000000

C 5.057215000000 5.646357000000 6.621958000000

H 5.830762000000 6.440321000000 6.726220000000

H 4.587720000000 5.745911000000 5.616881000000

C 8.282277000000 4.097276000000 9.753785000000

H 9.356668000000 4.147477000000 9.519400000000

C 1.859036000000 5.811201000000 6.649760000000

C 2.948118000000 6.602261000000 7.363697000000

H 2.569835000000 6.951027000000 8.345987000000

H 3.240003000000 7.491994000000 6.763834000000

H 0.037223000000 4.071319000000 6.164983000000

H 0.144229000000 3.242653000000 7.757065000000

H 5.151881000000 2.020746000000 8.163810000000

H 5.681767000000 1.532270000000 6.512804000000

C 1.653715000000 5.633093000000 10.665682000000

H -0.023037000000 7.008222000000 10.689130000000

H 1.098284000000 4.876222000000 10.089593000000

H 3.561824000000 0.300582000000 7.430695000000

H 3.410974000000 1.019282000000 5.791335000000

H 2.200249000000 5.476261000000 5.642419000000

H 0.954914000000 6.447943000000 6.511875000000

**1^Na^**

P 2.657083000000 3.872976000000 10.670788000000

Na 3.250408000000 4.189935000000 7.869543000000

O 1.185333000000 3.078072000000 6.996467000000

O 3.655594000000 1.904264000000 7.231410000000

O 4.986972000000 4.056845000000 6.196086000000

O 1.556158000000 5.758624000000 7.403580000000

O 4.222689000000 6.419712000000 7.490803000000

C 1.188719000000 6.227522000000 10.518797000000

H 0.400422000000 5.546880000000 10.158940000000

C 3.441912000000 6.604074000000 11.316681000000

H 4.428984000000 6.235265000000 11.630285000000

C 2.472020000000 5.684468000000 10.823408000000

C 4.432643000000 3.555121000000 10.380748000000

C 3.163142000000 7.974142000000 11.439208000000

H 3.946799000000 8.650485000000 11.818436000000

C 5.437047000000 4.480810000000 9.961622000000

H 5.174034000000 5.534487000000 9.795302000000

C 0.904580000000 7.591211000000 10.656325000000

H -0.101999000000 7.964077000000 10.404949000000

C 4.722733000000 1.693476000000 6.318472000000

H 4.323078000000 1.645398000000 5.277187000000

H 5.256283000000 0.735357000000 6.525271000000

C 1.304926000000 1.800782000000 7.619351000000

C 2.500562000000 1.106835000000 6.998598000000

H 2.616278000000 0.087849000000 7.437457000000

H 2.337173000000 0.991137000000 5.900993000000

C 0.296208000000 5.283262000000 6.960826000000

H 0.249227000000 5.281495000000 5.844517000000

H -0.531873000000 5.929669000000 7.336792000000

C 0.120395000000 3.875997000000 7.499688000000

H 0.150652000000 3.891751000000 8.614351000000

H -0.871730000000 3.474662000000 7.181567000000

C 1.897266000000 8.488246000000 11.100277000000

H 1.682751000000 9.563783000000 11.195069000000

C 4.850234000000 2.191745000000 10.493775000000

H 4.105358000000 1.438114000000 10.800776000000

C 5.700479000000 2.843795000000 6.441668000000

C 5.804572000000 5.222386000000 6.184580000000

H 6.428967000000 5.261792000000 5.260208000000

H 6.488821000000 5.224415000000 7.063786000000

C 6.165473000000 1.790370000000 10.242493000000

H 6.436841000000 0.727842000000 10.354683000000

C 6.760064000000 4.076519000000 9.723523000000

H 7.502903000000 4.831817000000 9.417200000000

C 4.897634000000 6.434892000000 6.238099000000

H 5.508271000000 7.362290000000 6.128240000000

H 4.177365000000 6.398947000000 5.386996000000

C 7.147307000000 2.731382000000 9.865884000000

H 8.188234000000 2.420853000000 9.688513000000

C 1.905704000000 7.067683000000 6.988391000000

H 1.980909000000 7.112099000000 5.874043000000

H 1.134179000000 7.812028000000 7.298824000000

C 3.222004000000 7.422758000000 7.670244000000

H 3.064264000000 7.487460000000 8.764094000000

H 3.577879000000 8.414932000000 7.309039000000

H 0.391869000000 1.180764000000 7.453513000000

H 1.450585000000 1.929816000000 8.719245000000

H 6.160296000000 2.866989000000 7.456379000000

H 6.517630000000 2.702345000000 5.695411000000

**1^K^**

K -2.508807000000 8.984545000000 27.855497000000

O -3.758857000000 9.191514000000 30.514823000000

O -0.958309000000 8.962230000000 30.154395000000

O 0.191932000000 7.854697000000 27.840587000000

O -1.468415000000 7.576852000000 25.566043000000

C -3.677764000000 7.507960000000 24.651309000000

O -4.286801000000 7.706701000000 25.920318000000

O -5.296987000000 9.197323000000 28.109619000000

P -2.207145000000 12.297205000000 28.355220000000

C -2.819656000000 9.803397000000 31.389694000000

C -4.906836000000 10.009983000000 30.325580000000

C -1.553394000000 8.978932000000 31.440389000000

C 0.367992000000 8.462205000000 30.142502000000

C 0.946304000000 8.642311000000 28.758370000000

C 0.698235000000 7.907650000000 26.517793000000

C -0.160920000000 7.030142000000 25.635687000000

C -2.370695000000 6.769916000000 24.825267000000

H -3.494782000000 8.487546000000 24.152914000000

H -4.344100000000 6.905054000000 23.986450000000

C -5.465834000000 8.499520000000 25.829267000000

C -6.143770000000 8.543842000000 27.178551000000

C -5.874575000000 9.308734000000 29.402091000000

C -2.534567000000 11.886280000000 26.613294000000

C -0.406980000000 12.149296000000 28.603484000000

H -3.239301000000 9.886344000000 32.422659000000

H -2.582715000000 10.830767000000 31.026607000000

H -4.606822000000 10.994655000000 29.892419000000

H -5.419401000000 10.202160000000 31.299905000000

H -0.863847000000 9.441153000000 32.186894000000

H -1.775201000000 7.939446000000 31.787213000000

H 0.384019000000 7.382117000000 30.430250000000

H 1.005704000000 9.019771000000 30.868374000000

H 0.916235000000 9.717154000000 28.469040000000

H 2.015359000000 8.319993000000 28.767742000000

H 1.752133000000 7.538070000000 26.478917000000

H 0.700516000000 8.956220000000 26.133125000000

H 0.297686000000 6.969888000000 24.618690000000

H -0.189403000000 5.994683000000 26.054230000000

H -2.541403000000 5.794381000000 25.343760000000

H -1.957967000000 6.541918000000 23.812597000000

H -6.178722000000 8.060988000000 25.089455000000

H -5.213971000000 9.531756000000 25.489612000000

H -7.113227000000 9.089294000000 27.074662000000

H -6.377237000000 7.506623000000 27.523460000000

H -6.122875000000 8.296771000000 29.806053000000

H -6.823444000000 9.896446000000 29.359471000000

C -3.828102000000 12.245496000000 26.114331000000

C -1.705990000000 11.152768000000 25.707747000000

C 0.617343000000 12.274180000000 27.618296000000

C 0.039796000000 12.001156000000 29.951730000000

H -4.510847000000 12.801889000000 26.778733000000

C -4.241392000000 11.929993000000 24.816706000000

C -2.120823000000 10.841495000000 24.404592000000

H -0.715542000000 10.804676000000 26.035309000000

H 0.342706000000 12.465600000000 26.571239000000

C 1.978482000000 12.200661000000 27.952790000000

C 1.396883000000 11.942562000000 30.286941000000

H -0.714150000000 11.933087000000 30.752369000000

H -5.242464000000 12.244282000000 24.478167000000

C -3.387157000000 11.232690000000 23.933901000000

H -1.439980000000 10.272162000000 23.751526000000

H 2.733277000000 12.300104000000 27.155120000000

C 2.388346000000 12.019681000000 29.286818000000

H 1.689767000000 11.829211000000 31.343829000000

H -3.705255000000 11.002890000000 22.905543000000

H 3.456510000000 11.959610000000 29.545837000000

**1^Rb^**

Rb 6.592246000000 9.793522000000 13.992006000000

P 8.450880000000 10.537404000000 11.185398000000

O 7.542102000000 12.281318000000 15.359461000000

O 4.829110000000 7.503228000000 13.919601000000

O 7.057848000000 7.348509000000 15.700747000000

O 8.579519000000 9.713821000000 16.061636000000

O 3.609469000000 10.048702000000 13.488262000000

O 5.303619000000 12.356542000000 13.552868000000

C 6.669653000000 6.277973000000 14.846496000000

H 6.929398000000 5.292428000000 15.303513000000

H 7.198034000000 6.343533000000 13.864519000000

C 5.172637000000 6.322871000000 14.627563000000

H 4.871984000000 5.415740000000 14.049128000000

H 4.643777000000 6.288045000000 15.611223000000

C 3.173171000000 8.861572000000 12.838887000000

H 3.702881000000 8.735685000000 11.865494000000

H 2.077929000000 8.909919000000 12.622717000000

C 11.699132000000 12.040078000000 13.179524000000

H 12.244556000000 12.996754000000 13.129420000000

C 3.895005000000 12.437049000000 13.422602000000

H 3.600498000000 13.338613000000 12.832069000000

H 3.407818000000 12.520740000000 14.425201000000

C 5.893513000000 13.512422000000 14.131168000000

H 5.403985000000 13.757372000000 15.105044000000

H 5.767891000000 14.395063000000 13.458229000000

C 9.881758000000 10.594219000000 12.314105000000

C 8.914237000000 12.053150000000 15.671210000000

H 9.369656000000 12.967016000000 16.125201000000

H 9.490254000000 11.813706000000 14.749866000000

C 12.089184000000 11.055001000000 14.111750000000

H 12.938905000000 11.225084000000 14.790434000000

C 9.032952000000 10.916606000000 16.662993000000

H 10.105474000000 10.826261000000 16.958833000000

H 8.444496000000 11.140461000000 17.586820000000

C 8.442998000000 7.329317000000 16.017269000000

H 9.056708000000 7.315718000000 15.084506000000

H 8.703775000000 6.413906000000 16.602700000000

C 7.371553000000 13.268757000000 14.348867000000

H 7.848161000000 12.938944000000 13.393226000000

H 7.850069000000 14.231157000000 14.652641000000

C 8.795717000000 8.552112000000 16.840035000000

H 8.183073000000 8.576211000000 17.774891000000

H 9.867230000000 8.472377000000 17.145913000000

C 6.507087000000 8.597354000000 10.773843000000

H 5.875798000000 9.481395000000 10.574390000000

C 3.434493000000 7.656831000000 13.717967000000

H 2.905014000000 7.769608000000 14.695844000000

H 3.007848000000 6.758862000000 13.208188000000

C 3.385777000000 11.209563000000 12.697436000000

H 2.295412000000 11.342448000000 12.493026000000

H 3.901768000000 11.116520000000 11.710941000000

C 11.366886000000 9.848301000000 14.145767000000

H 11.639864000000 9.065435000000 14.872642000000

C 5.950271000000 7.318923000000 10.658017000000

H 4.892252000000 7.213823000000 10.366851000000

C 8.076618000000 6.343611000000 11.269602000000

H 8.713414000000 5.461148000000 11.448136000000

C 10.630334000000 11.813795000000 12.305789000000

H 10.350872000000 12.597932000000 11.581246000000

C 10.290503000000 9.624249000000 13.275946000000

H 9.737489000000 8.679168000000 13.361929000000

C 8.641538000000 7.623648000000 11.372541000000

H 9.715360000000 7.708793000000 11.591717000000

C 7.872694000000 8.806562000000 11.147535000000

C 6.723626000000 6.170653000000 10.919623000000

H 6.285326000000 5.163970000000 10.841989000000

**1^Cs^**

Cs 14.224515000000 9.900000000000 12.939990000000

P 13.894255000000 12.539224000000 15.345355000000

O 12.148111000000 10.725401000000 10.875174000000

O 16.505942000000 8.360869000000 11.595721000000

O 11.090746000000 9.538713000000 13.277464000000

O 15.266249000000 7.069255000000 13.907150000000

O 12.922950000000 8.249587000000 15.093500000000

O 14.625894000000 9.675985000000 9.804803000000

C 16.901463000000 7.108033000000 12.138781000000

C 10.511539000000 10.659404000000 12.625974000000

C 16.660948000000 7.086738000000 13.633625000000

C 12.484547000000 10.727459000000 9.498495000000

C 13.493944000000 7.030894000000 15.538761000000

C 11.524371000000 8.342723000000 15.314177000000

C 10.761707000000 10.577115000000 11.133960000000

C 15.748592000000 13.413235000000 13.242526000000

C 13.369192000000 13.518278000000 12.814244000000

C 16.426197000000 11.022600000000 15.355030000000

C 10.996474000000 9.620694000000 14.698583000000

C 14.988939000000 7.052724000000 15.305971000000

C 13.985221000000 10.861027000000 9.342724000000

C 14.422899000000 13.178039000000 13.718495000000

C 13.612811000000 14.003782000000 11.525960000000

C 16.039247000000 9.722555000000 9.668483000000

C 14.924981000000 10.846870000000 17.244264000000

C 16.989291000000 9.540447000000 17.221227000000

C 16.644729000000 8.433080000000 10.183992000000

C 15.994056000000 13.880316000000 11.940731000000

C 15.785014000000 9.928661000000 17.850648000000

C 15.206085000000 11.442188000000 15.971443000000

C 14.934477000000 14.170434000000 11.061445000000

C 17.289146000000 10.097182000000 15.966596000000

H 16.717241000000 11.426855000000 14.376013000000

H 18.222797000000 9.820013000000 15.449386000000

H 17.673934000000 8.825411000000 17.701916000000

H 15.518216000000 9.508226000000 18.834149000000

H 13.989718000000 11.128315000000 17.758326000000

H 12.328203000000 13.367208000000 13.146178000000

H 12.761359000000 14.234025000000 10.865201000000

H 15.132324000000 14.535876000000 10.042070000000

H 17.035649000000 14.040536000000 11.616406000000

H 16.604227000000 13.267276000000 13.915903000000

H 17.987911000000 6.925650000000 11.952966000000

H 16.334261000000 6.276832000000 11.654102000000

H 17.143496000000 7.973201000000 14.110558000000

H 17.153763000000 6.176460000000 14.054667000000

H 12.133380000000 9.789499000000 9.003094000000

H 11.997681000000 11.586239000000 8.974949000000

H 13.032380000000 6.164082000000 15.004705000000

H 13.315214000000 6.886611000000 16.631998000000

H 11.298415000000 8.353110000000 16.408202000000

H 10.998913000000 7.462263000000 14.869690000000

H 10.388264000000 9.601353000000 10.737358000000

H 10.178330000000 11.387917000000 10.633553000000

H 15.437777000000 7.941657000000 15.808412000000

H 15.431822000000 6.142664000000 15.779417000000

H 16.327821000000 9.845808000000 8.596174000000

H 16.465090000000 10.594965000000 10.223756000000

H 14.342750000000 11.751047000000 9.915512000000

H 14.217862000000 11.037380000000 8.264646000000

H 9.407799000000 10.690549000000 12.799724000000

H 10.935578000000 11.611531000000 13.026047000000

H 11.581815000000 10.498022000000 15.075045000000

H 9.933450000000 9.753736000000 15.013781000000

H 17.722634000000 8.399789000000 9.891805000000

H 16.138798000000 7.565278000000 9.696299000000

**4^Cs^**

C 4.31687370874961 4.59775778730645 3.90030333142770

C 5.24056114014020 3.49688529568445 3.93914021214135

H 4.96520679688310 2.53630610007281 3.48188250258073

C 6.50192272184317 3.59699685518050 4.55042626421816

H 7.16406664405575 2.71415781197685 4.55200091399050

C 6.93272724035120 4.79474485233498 5.15267299197263

H 7.92546806274056 4.87123503649635 5.62182671017655

C 6.05836401622095 5.90882275428658 5.10390780997933

H 6.37171829915740 6.87238620951975 5.53979897426730

C 4.80427955255872 5.81332163691748 4.49935145136314

H 4.14964856402424 6.70179875916707 4.48515190537005

C 2.22944408403675 2.92378470626417 2.51572464389964

C 2.25862029187544 1.76556436997879 3.53180637400495

H 3.26692299595805 1.60474179559001 3.96659058886874

H 1.96628010964270 0.80229322759898 3.05029734433845

H 1.54642660105754 1.95139210261711 4.36138491114467

C 3.13931368621732 2.61774124998626 1.31220332560018

H 3.07650854756551 3.42705181894389 0.55594975400303

H 2.84276870673601 1.66318328392858 0.81623919057445

H 4.20535670061505 2.52095185001715 1.60017946695644

C 0.77997818653327 3.06792338824447 2.00909209916099

H 0.07807135884029 3.28356817875162 2.84329029624576

H 0.43796346474202 2.13131734904953 1.51330870960112

H 0.68722826074708 3.89524446553330 1.27433117113327

C 5.74278795524346 2.07656728350290 9.47159197068859

H 6.66963834135609 1.46364034748811 9.58761962687228

H 5.24040885662091 2.11551534330466 10.46822190186639

C 6.13593416036539 3.47729821250334 9.05151299630255

H 6.94576096014374 3.83152557255715 9.73481398259775

H 6.55756094372129 3.46838015642864 8.01606325154359

C 5.32895043080352 5.69363790329766 8.83497251279250

H 5.80281789677890 5.77137020074079 7.82795131237047

H 6.05773995147195 6.09146991951548 9.58286768180312

C 4.07826817616434 6.54631048965083 8.88652126098283

H 3.57615137520579 6.42756309985139 9.87818570375208

H 4.38224489048987 7.61739786884989 8.79180568509034

C 2.01514607268980 6.95669585977802 7.77359521026707

H 2.25805913353393 8.02206670458063 7.54145707467101

H 1.48351015917958 6.93833426064159 8.75615105263821

C 1.10607193464184 6.41923905760371 6.68853501214147

H 0.27124467160718 7.14623215422892 6.53616453362235

H 1.65906505288301 6.33353678206901 5.72096098656552

C -0.27665029742015 4.59380282753962 6.09545724501594

H 0.25278111362690 4.52164416153409 5.11271209666277

H -1.17237813415714 5.24759532651664 5.95480215485938

C -0.75328729765564 3.22988479019012 6.54495911414701

H -1.18698917132135 3.29778824616248 7.57216631459271

H -1.56530663964900 2.88979949609396 5.85762521403955

C -0.01223628483531 1.02179409724414 7.02233052704779

H -0.82649075674476 0.56486552607180 6.40852540635392

H -0.38427164519916 1.09097222280026 8.07355351997497

C 1.19646513738097 0.11042958848458 6.96517680295853

H 0.87090201817321 -0.92404851692975 7.23380604821350

H 1.59612123298869 0.07241208289501 5.92299479019445

C 3.34709073093345 -0.25924548175593 7.89011113406953

H 3.83013437960552 -0.29527676800872 6.88219586661543

H 3.07005354876912 -1.30768648414675 8.15846151699520

C 4.33317079399727 0.25169340063891 8.91982420234939

H 3.82016197754899 0.36022032429999 9.90602381304460

H 5.14055308346897 -0.51054272530599 9.04550085361623

Cs 3.18040163369589 3.37186238718013 6.73053160947928

O 0.59433089108462 5.14772695622911 7.07808111700130

O 0.32837216500300 2.30623749462349 6.52920888015613

O 2.19348074362552 0.57016491804565 7.86894790160023

O 4.87726429878921 1.49570178342512 8.50504970160847

O 5.00685535906532 4.33760656598633 9.12818363554344

O 3.20151668002444 6.17856410291140 7.83592563438741

P 2.61607373500831 4.64418157523382 3.29489219988448

**5^Cs^**

Cs 4.30469630729134 2.84429235854096 16.88608628851023

P 2.07039094310311 2.33435751577674 14.34511302542434

O 6.76965655710915 2.18731919408379 15.24473206408920

O 5.59683436734732 4.83498742775806 14.80226808899824

O 4.79554422622800 2.14924260375992 19.87961228307135

O 3.65423389747372 4.76952315745785 19.34164996596176

O 6.87461406797784 1.34581410816008 18.00767629664075

O 3.56248961208440 5.76278850517666 16.66029457803534

C 5.95742978245104 3.91715534607188 13.77309772195064

H 6.24211749412813 4.46765507221451 12.84244079686111

H 5.08390729650050 3.26327374708044 13.52451954229276

C 0.70265361704687 4.53368113775557 13.01544780942464

H 1.61925405275625 5.13821093422509 13.17645337419943

H -0.16473200720880 5.23701153169308 12.97895450593623

H 0.78756099621649 4.06694228665676 12.01457344568635

C 0.52629403488715 3.48237047642610 14.13141293565135

C 2.10140858589860 1.02355125215257 12.92537680181550

C 7.86387216894424 1.49263262598937 15.81804285448819

H 8.61497412574367 2.21020934377747 16.23032711575390

H 8.38582881025630 0.86945625761918 15.05110473970472

C 5.97292860454533 1.43865584458743 20.23556275089388

H 5.79046439224055 0.79754446311975 21.13239204120165

H 6.79448088111088 2.14935668139764 20.49538251465111

C 6.41915050257451 0.54989969981665 19.09255815687613

H 7.23781328036321 -0.11309459984996 19.46485875455746

H 5.57835294906713 -0.11505909101542 18.77341340533188

C 7.14688375024831 3.08889934067893 14.20978059322941

H 7.53411069790772 2.52629208677755 13.32586827717065

H 7.96539796969428 3.76226688965370 14.56312815982884

C 3.21564088835600 3.90182782885114 20.37658743759864

H 2.39259783183305 3.23467404771774 20.01758073094379

H 2.80843804360606 4.48747977716055 21.23658856184977

C -0.79730225260236 2.73214933425041 13.90652075402148

H -0.82625012584420 2.23258868681233 12.91633113942581

H -1.66679421653114 3.43246006435928 13.93852392825795

H -0.95803872141059 1.95444520782301 14.68150651949314

C 0.44585826074290 4.23422723764218 15.47773726582481

H 0.22916586819858 3.53753108385583 16.31508373038307

H -0.36001482367203 5.00489626582191 15.45591050298326

H 1.40159496263883 4.75212968873841 15.70946181489363

C 3.18312218810275 6.53894463577574 17.78263197995817

H 2.39273645729352 7.27841091642921 17.50521370348221

H 4.05551817704544 7.11388535923125 18.17876659880988

C 4.37898102046416 3.07060038280447 20.87802443544729

H 5.22163915888231 3.74645370193132 21.16162880876445

H 4.05825171548630 2.53142817122483 21.80278587332733

C 2.62881896549575 5.63211376156971 18.86280233349908

H 2.23141500850006 6.26784215744891 19.69113926805430

H 1.77107123934270 5.04133312209199 18.45833285235767

C 7.37501466783876 0.57601158789825 16.92121444154669

H 6.58607357315922 -0.10882640884260 16.52304868872902

H 8.22830405231707 -0.06322472684332 17.25485793921847

C 4.45303208009889 5.60586739774636 14.43441603414971

H 3.60853305698600 4.91608575354629 14.17799394048631

H 4.67761341318664 6.23126494646923 13.53534747359096

C 4.05886049611925 6.52687899573398 15.56763543834300

H 4.93349307216304 7.14240700767310 15.89156399278578

H 3.27304770677265 7.22827208526894 15.19588523159903

C 1.95409206897975 1.62530422658954 11.51721365193478

H 0.93977947763386 2.04269972373583 11.35038444533544

H 2.11811305071303 0.85361247047916 10.72694362791210

H 2.68594333009546 2.44337162284822 11.35298987625528

C 1.06218771634038 -0.10101516289167 13.11136836838529

H 1.16333239550087 -0.57180903301797 14.11110324490613

H 1.18545240656605 -0.90062574510223 12.34045581435628

H 0.02353100801839 0.27432832720971 13.02774995390040

C 3.50504548460407 0.39307304574012 13.04413996551541

H 4.30507431224575 1.13033127270309 12.82353203629683

H 3.62163776291737 -0.45906172023352 12.33508596452015

H 3.68527725782782 -0.00050329579340 14.06898274261870

1. a) R. Neufeld, D. Stalke, *Chem. Sci.* **2015**, *6*, 3354-3364; b) S. Bachmann, B. Gernert, D. Stalke, *Chem. Commun.* **2016**, *52*, 12861-12864; c) S. Bachmann, R. Neufeld, M. Dzemski, D. Stalke, *Chem. Eur. J.* **2016**, *22*, 8462-8465. [↑](#footnote-ref-1)
